# Ovulatory signal-triggered chromatin remodeling in ovarian granulosa cells by HDAC2 phosphorylation activation-mediated histone deacetylation

**DOI:** 10.1186/s13072-023-00485-8

**Published:** 2023-04-19

**Authors:** Jiamin Jin, Peipei Ren, Xiang Li, Yinyi Zhang, Weijie Yang, Yerong Ma, Mengru Lai, Chao Yu, Songying Zhang, Yin-Li Zhang

**Affiliations:** 1 Assisted Reproduction Unit, Department of Obstetrics and Gynecology, School of Medicine, Sir Run Run Shaw Hospital, Zhejiang University, Hangzhou, 310016 China; 2 Key Laboratory of Reproductive Dysfunction Management of Zhejiang Province, Hangzhou, 310016 China; 3 College of Life Science, Zhejiang University, Hangzhou, 310058 China

**Keywords:** Luteinizing hormone, Granulosa cells, HDAC2, H3K27Ac, Ovulation, Chromatin remodeling

## Abstract

**Background:**

Epigenetic reprogramming is involved in luteinizing hormone (LH)-induced ovulation; however, the underlying mechanisms are largely unknown.

**Results:**

We here observed a rapid histone deacetylation process between two waves of active transcription mediated by the follicle-stimulating hormone (FSH) and the LH congener human chorionic gonadotropin (hCG), respectively. Analysis of the genome-wide H3K27Ac distribution in hCG-treated granulosa cells revealed that a rapid wave of genome-wide histone deacetylation remodels the chromatin, followed by the establishment of specific histone acetylation for ovulation. HDAC2 phosphorylation activation coincides with histone deacetylation in mouse preovulatory follicles. When HDAC2 was silenced or inhibited, histone acetylation was retained, leading to reduced gene transcription, retarded cumulus expansion, and ovulation defect. HDAC2 phosphorylation was associated with CK2α nuclear translocation, and inhibition of CK2α attenuated HDAC2 phosphorylation, retarded H3K27 deacetylation, and inactivated the ERK1/2 signaling cascade.

**Conclusions:**

This study demonstrates that the ovulatory signal erases histone acetylation through activation of CK2α-mediated HDAC2 phosphorylation in granulosa cells, which is an essential prerequisite for subsequent successful ovulation.

**Supplementary Information:**

The online version contains supplementary material available at 10.1186/s13072-023-00485-8.

## Background

Ovarian follicle growth and ovulation depend on two gonadotropins secreted from the pituitary gland: follicle-stimulating hormone (FSH) and luteinizing hormone (LH) [1]. Granulosa cells (GCs), as the primary ovarian cells, are essential for the body’s response to FSH and LH to support oocyte development by producing sex steroids and various growth factors [2]. FSH initiates follicle recruitment and their development to ovulatory follicles. A mid-cycle surge of LH or induction with exogenous human chorionic gonadotropin (hCG) initiates ovulation, inducing oocyte meiosis resumption, cumulus expansion, and follicle rupture [3].

Several signal pathways are involved in ovulatory signal (LH or hCG)-induced ovulation. When LH or hCG binds to the same receptor, that is, LH receptor (encoded by *LHCGR*) [4], it activates the cAMP/protein kinase A (PKA) signaling pathway in mural GCs, thereby upregulating the expression of epidermal growth factor (EGF)-like factors, such as amphiregulin (AREG), epiregulin (EREG), and betacellulin (BTC) [5–7]. These factors bind the EGF receptor and activate the extracellular signal-regulated kinase 1/2 (ERK1/2) signal cascade in mural and cumulus GCs [8]. This signal cascade plays central roles in GCs for LH/hCG-triggered ovulation by initiating oocyte meiosis resumption, inducing cumulus cell expansion, and triggering 100-fold increases in the mRNA expression of many ovulatory genes, such as *Areg*, *Ereg*, *Sult1e1,* and *Star* [8]. We previously observed that epigenetic remodeling exists in LH/hCG-induced ovulation to facilitate the active transcription of substantial genes. LH/hCG induction upregulates the expression of CCAAT/enhancer-binding protein α/β (C/EBPα/β) and enhances its binding to CBP/P300 (encoded by *KAT3A*/*B*), two lysine acetyltransferases (KATs) [9, 10]. Then, CBP/P300 catalyzes the acetylation of various histones by docking the promoters of ovulatory genes [8–10]. LH/hCG induces extensive chromatin remodeling and transcriptome switching at the early ovulation stage, leading to GC reprogramming and follicular state switching [11]. Despite intensive studies on the physiology of LH-mediated ovulation, the mechanism of epigenetic regulation involved in ovulation is not well understood.

Histone acetylation is a dynamic epigenetic modification regulating chromatin conformation and transcription. Of the multiple histone acetylation modifications, histone H3 acetylation at lysine 27 (H3K27Ac) is used as a marker for the active promoter and distal enhancer that facilitate active transcription [12, 13]. H3K27Ac is catalyzed mainly by CBP/P300 [14, 15] and is deacetylated by the histone deacetylase (HDAC) complex, for example, nucleosome remodeling and deacetylase (NuRD), a multiprotein complex containing HDAC1 and HDAC2 [16, 17]. HDAC1 and HDAC2 remove acetyl groups added by KATs, which are recruited by elongating RNA polymerase II, to “reset” chromatin [18]. Although KATs and HDACs are both located at the transcribed regions of active genes, HDACs are believed to remove acetyl groups at active genes and repress gene expression [18, 19]. In addition to the expression level, HDAC2 activity is regulated by Ser394 phosphorylation, which is mediated mainly by *Csnk2a1*-encoded CK2α, a protein kinase [20, 21]. Phosphorylated HDAC2, but not unmodified HDAC2, can form the NuRD complex [22]. CK2α overexpression in GCs is involved in the occurrence of the polycystic ovarian syndrome (PCOS), a disease exhibiting hyperandrogenism and abnormal ovulation [23]. However, the physiological roles of CK2α and HDAC2 in follicle development and ovulation have not been investigated.

We here demonstrated a genome-wide histone deacetylation process between two active transcription waves separately mediated by FSH-mediated follicle growth and LH-mediated ovulation. LH/hCG promotes activation of HDAC2 phosphorylation to non-selectively deacetylate histones, which is essential for the subsequent establishment of histone acetylation and gene transcription for ovulation. HDAC2 phosphorylation was found to be dependent on CK2α. When CK2α was inhibited or HDAC2 was depleted, H3K27 deacetylation was blocked and ovulation-induced gene expression was attenuated. This study offers evidence that CK2α/HDAC2-mediated histone deacetylation is a key step in chromatin remodeling for subsequent transcriptional switching during LH/hCG-induced ovulation.

## Results

### The ovulatory signal induces H3K27Ac reprogramming during ovarian GCs luteinization

**Fig. 1:**
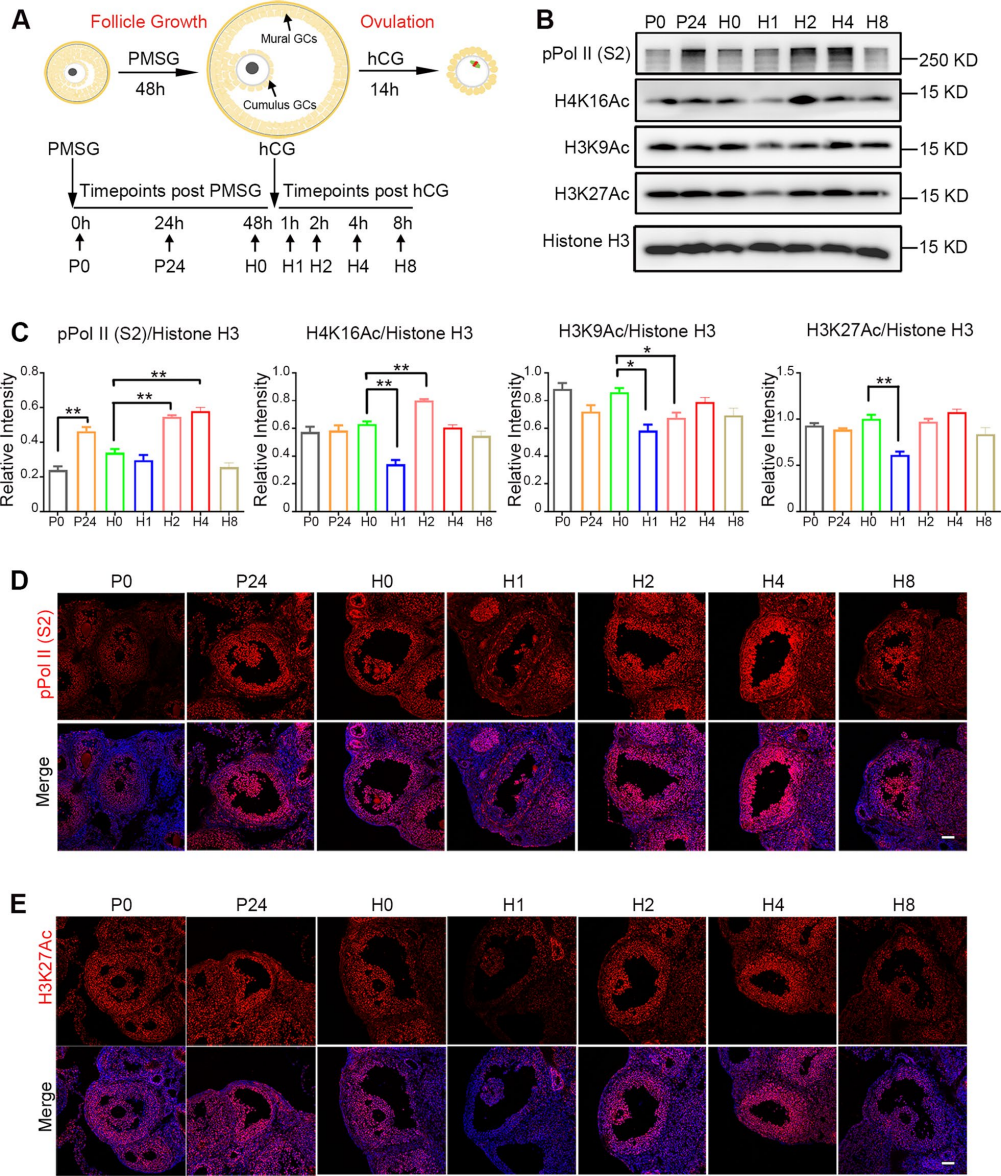
Transcription and Histone Acetylation Dynamics during the Process of Follicle Growth and Ovulation. **A** Depiction of the controlled ovarian hyperstimulation protocol in a mouse. Immature female mice were primed with pregnant mare chorionic gonadotropin (PMSG) to stimulate follicular growth, followed by injection with human chorionic gonadotropin (hCG) 48 h later to induce ovulation. At the indicated timepoints, the ovaries were collected for analysis. P0, P24, H0: 0 h, 24 h, and 48 h after PMSG treatment. H1-8: 1 h, 2 h, 4 h, and 8 h after hCG treatment, equal to LH surge. The arrowheads indicate two types of granulosa cells (GCs) in the antral follicle. **B** The levels of transcription and histone acetylation were dramatically downregulated between the two surges mediated by PMSG and hCG. The ovaries at the indicated timepoints were collected and lysed for Western blotting with antibodies against phosphorylated RNA polymerase CTD S2 (pPol II(S2)), H3K27Ac, H4K16Ac, H3K9Ac, and histone H3. **C** Quantitative analysis of pPol II(S2), H3K27Ac, H4K16Ac, and H3K9Ac levels with histone H3 normalization in panel B using the ImageJ software. The data were expressed as the mean ± SD. *P* value was determined by two-way ANOVA, followed by Tukey’s post-test. *P < 0.05, **P < 0.01. **D**, **E** The immunofluorescence results exhibiting the dynamics of pPol II (S2) (D, red) and H3K27Ac (E, red) in ovarian antral follicles at the indicated timepoints post-PMSG or/and hCG treatment. The nuclei were stained with DAPI (blue). Scale bar = 100 μm. N = 3 biologically independent experiments

To investigate the mechanism underlying LH/hCG-triggered ovulation, a standard ovarian stimulating protocol was used to induce ovulation in a mouse model. The mice were injected with pregnant mare serum gonadotrophin (PMSG to stimulate ovarian follicle growth. After 48 h, the mice were injected with hCG to induce ovulation (Fig. 1A). With this protocol, along with terminal differentiation and morphological changes in GCs, ovulation occurs 12–14 h after hCG administration. To identify the transcriptional state of antral follicles following PMSG and hCG treatment, we detected the levels of RNA polymerase II phosphorylated at Ser2 at the C-terminal repeat domain (pPol II(S2)), a transcription indicator. Two waves of active transcription were observed under the influence of PMSG and hCG, with the summit at 24 h after PMSG injection (named P24) and at 4 h after hCG injection (named H2 and H4), respectively (Fig. 1B, C and D). However, the transcriptional activity decreased at 48 h after PMSG injection (also named H0) and at 8 h after hCG injection (also named H8). Because histone acetylation is closely associated with active transcription, we examined histone acetylation dynamics in mouse ovaries at different time points. Histone acetylation modifications, including H3K27Ac, H3K9Ac, and H4K16Ac, were strong at 24–48 h after PMSG injection and at 2–8 h after hCG injection (Fig. 1B, C, E and Additional file 1: Fig. S1). However, histone acetylation dramatically decreased at 1 h after hCG injection (Fig. 1B, C, E, and Additional file 1: Fig. S1), indicating that histone deacetylation occurs rapidly after ovulatory signal induction.

**Fig. 2:**
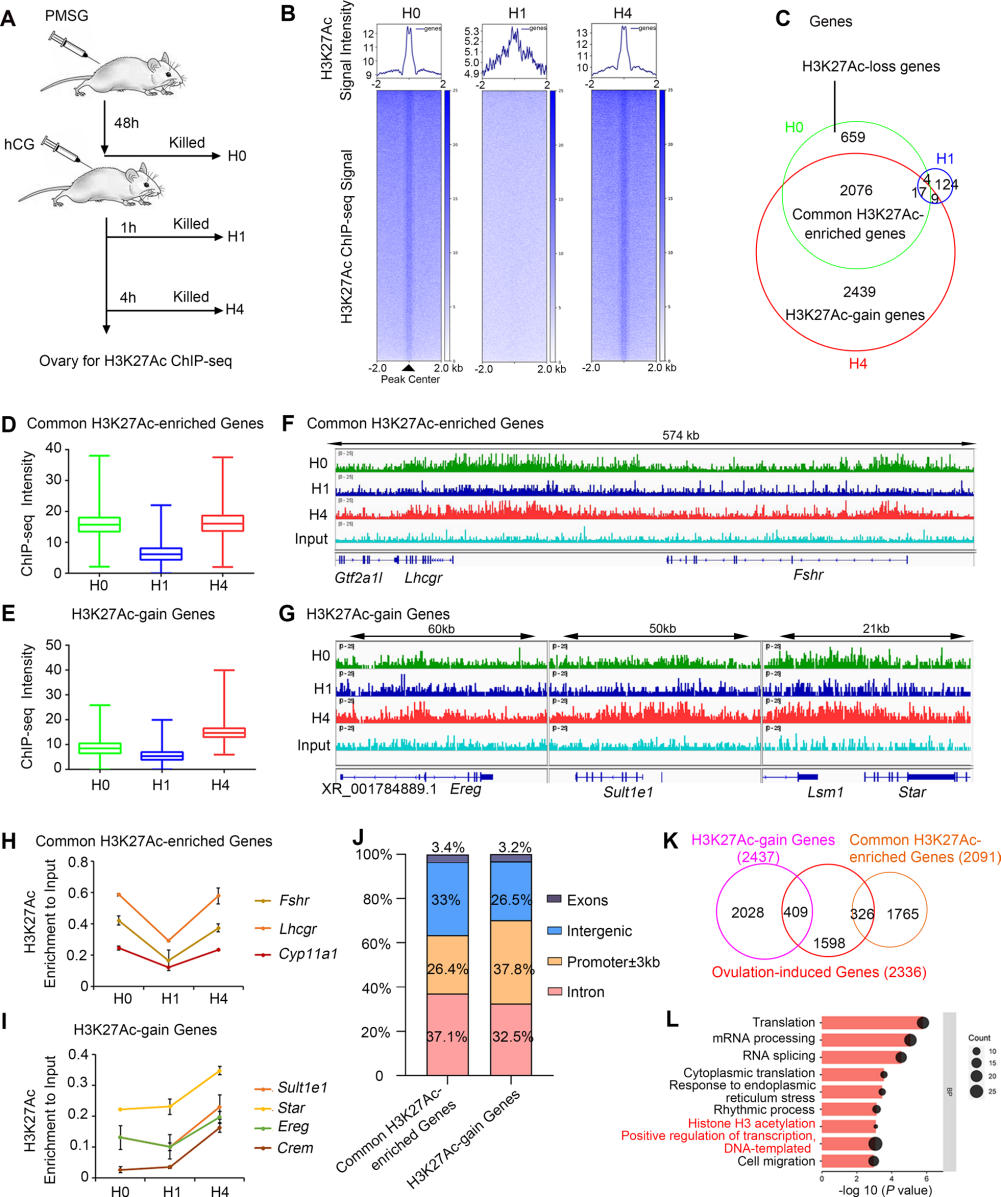
The Ovulatory Hormone Signal Induces Genome-wide H3K27Ac Deacetylation and Subsequent Histone Acetylation. **A** A diagram exhibiting the sample collection for H3K27Ac ChIP-seq. H0, H1, H4: 0 h, 1 h and 4 h after hCG treatment. **B** H3K27Ac enrichment around peak center for ovaries at 0 h, 1 h, and 4 h post-hCG treatment. The upper panels show the average signal profile around detected peak centers (± 2 kb). The lower heatmap shows H3K27Ac read density for a total of 9,868 shared peaks of H0 and H4 around the peak centers. Two independent ChIP-seq experiments were sequenced, as were two matched input controls. **C** A Venn diagram of H3K27Ac ChIP-seq genes. H3K27Ac ChIP-seq was performed using the ovaries at 0 h, 1 h, and 4 h post-hCG treatment. The numbers indicate the total genes identified in each timepoint. **D**, **E** The box blot represents the average RPKM (reads per kilobase per million) of common H3K27Ac-enriched genes (**D**) and H3K27Ac-gain genes (**E**). The data are presented as the mean ± SD. **F**, **G** The UCSC Genome Browser tracks demonstrating the occupancy of H3K27Ac on *Fshr* and *Lhcgr* genes (representing the common H3K27Ac-enriched genes before and after ovulatory signal trigger) and *Ereg*, *Sult1e1*, and *Star* genes (belonging to the H3K27Ac-gain genes). **H**, **I** The ChIP-qPCR validation of common H3K27Ac-enriched genes and H3K27Ac-gain at 0, 1, and 4 h post-hCG injection. **J** The stacked bar chart showing the genomic distribution of H3K27Ac occupancy of common H3K27Ac-enriched genes and H3K27Ac-gain genes. **K** Venn diagram depicting the overlapped gene number of significantly upregulated genes after hCG induction (extracted from GSE119508) with H3K27Ac-gain or common H3K27Ac-enriched genes. **L** Gene Ontology (GO) analysis demonstrates the biological process (BP) of upregulated genes with H3K27Ac-gain post-hCG induction

Since H3K27Ac is a key epigenetic mark for active transcription [24, 25], we performed chromatin immunoprecipitation coupled with high-throughput sequencing (ChIP-seq) of mouse ovaries at 0, 1, and 4 h after hCG injection to identify the genome-wide chromatin occupancy of H3K27Ac (Fig. 2A). A total of peaks were identified as follow: 26528 peaks in H0 group, 6518 peaks in H1 group and 29735 peaks in H4 group. Differential peak analysis showed that upon hCG stimulation, the majority of H3K27Ac modification was erased at 1 h and re-established at 4 h as shown by the heatmap (Fig. 2B). This suggested that H3K27Ac reprogramming occurs in ovaries upon hCG administration. Furthermore, we annotated these H3K27Ac peaks using the input as a background control and identified 2735 and 4515 H3K27Ac-enriched genes at 0 and 4 h after hCG injection, respectively. By contrast, only 154 H3K27Ac-enriched genes were identified at 1 h after hCG injection (Fig. 2C). Of the 4515 genes identified at 4 h after hCG injection, 2026 genes were also found at 0 h after hCG treatment, which exhibited a comparable H3K27Ac intensity between these two groups. In this study, these genes were defined as “common H3K27-enriched genes” before and after the ovulatory signal (Fig. 2C–E). Through an analysis of differentially H3K27Ac-enriched genes, 659 and 2439 genes were identified to be “H3K27Ac-loss genes” and “H3K27Ac-gain genes” after ovulation induction, respectively (Fig. 2C–E).

H3K27Ac dynamics in these two groups were analyzed: common H3K27Ac-enriched genes (Fig. 2D) and H3K27Ac-gain genes (Fig. 2E). H3K27 deacetylation occurred at common H3K27Ac-enriched genes, such as *Fshr* and *Lhcgr*, which are two well-known FSH target genes (Fig. 2F). Surprisingly, deacetylation also occurred in most H3K27Ac-gain genes at 1 h after hCG injection, but 4 h later, H3K27Ac was re-established at these genes with higher intensity or broader length, such as the LH-induced genes *Ereg*, *Sult1e1,* and *Star* (Fig. 2G). Consistent with ChIP-seq results, ChIP-qPCR results further confirmed the occurrence of H3K27 deacetylation at 1 h after hCG treatment (Fig. 2H and I). Of the H3K27Ac-gain peaks, 83% were associated with increased H3K27Ac intensity and 17% were de novo H3K27Ac-deposited peaks at 4 h after hCG injection compared with 0 h (Additional file 1: Fig. S2A and S2B). These results indicate that H3K27Ac was poised on many ovulation-induced genes before ovulatory signal induction. Gene Ontology (GO) analysis was performed for genes of the de novo H3K27Ac-deposited peaks. These genes were enriched in biological pathways linked to DNA repair, RNA polymerase II transcriptional preinitiation complex assembly, and histone acetylation (Additional file 1: Fig. S2C).

We then analyzed the H3K27Ac occupancy distribution of common H3K27Ac-enriched genes and H3K27Ac-gain genes. Approximately 26.4% (548 genes) of the common H3K27-enriched peaks were overlapped with promoters, and 37.8% of the peaks with an increased H3K27ac signal are found in the promoter region (Fig. 2J). To identify the biological networks of these H3K27Ac-gain genes, we performed the Kyoto Encyclopedia of Genes and Genomes (KEGG) pathway analysis. The H3K27Ac-gain genes were linked to cumulus expansion- and follicular rupture-related pathways, such as the neurotrophin, MAPK1/3, and VEGF signaling pathways (Additional file 1: Fig. S2D). Because H3K27Ac re-establishment requires many sequence-specific transcription factors and co-factors, we performed motif enrichment analysis on H3K27Ac-gain peaks using the Homer software (Heinz et al., 2010). This analysis revealed several known motifs and de novo motifs for transcription factors predicted to bind H3K27Ac-gain regions. Several transcription factors with known enriched motifs have been directly or indirectly validated in other studies, such as PR, FOXO1, and SMAD3 (Additional file 1: Fig. S2E). Notably, the de novo enriched motifs showed TATA-binding protein (TBP) and GRHL2. These two transcriptional factors, whose functions in ovulation are unclear, were possibly involved in H3K27Ac establishment after hCG induction (Additional file 1: Fig. S2E).

Analysis of previously reported RNA-seq data on ovarian GCs from mice at 0 and 4 h after hCG induction revealed 2336 upregulated protein-coding genes in GCs at 4 h compared with 0 h, which were ovulation-induced genes [11]. Notably, these ovulation-induced genes exhibited mRNA expression at a certain level at 0 h. Then, we overlapped of our common H3K27Ac-enriched genes and H3K27Ac-gain genes with ovulation-induced genes, and 326 and 459 genes were identified, respectively (Fig. 2K). We performed the GO analysis for 459 upregulated genes with H3K27Ac-gain. These genes were related to translation, mRNA processing, histone H3 acetylation, and positive regulation of transcription (Fig. 2L). According to these results, H3K27Ac appears to poise a subset of genes for ovulation signal-stimulated expression, and the ovulatory signal induces transient H3K27 deacetylation before establishing a more intense and wider H3K27Ac landscape.

### HDAC2 is phosphorylated to catalyze histone deacetylation after hCG induction

**Fig. 3:**
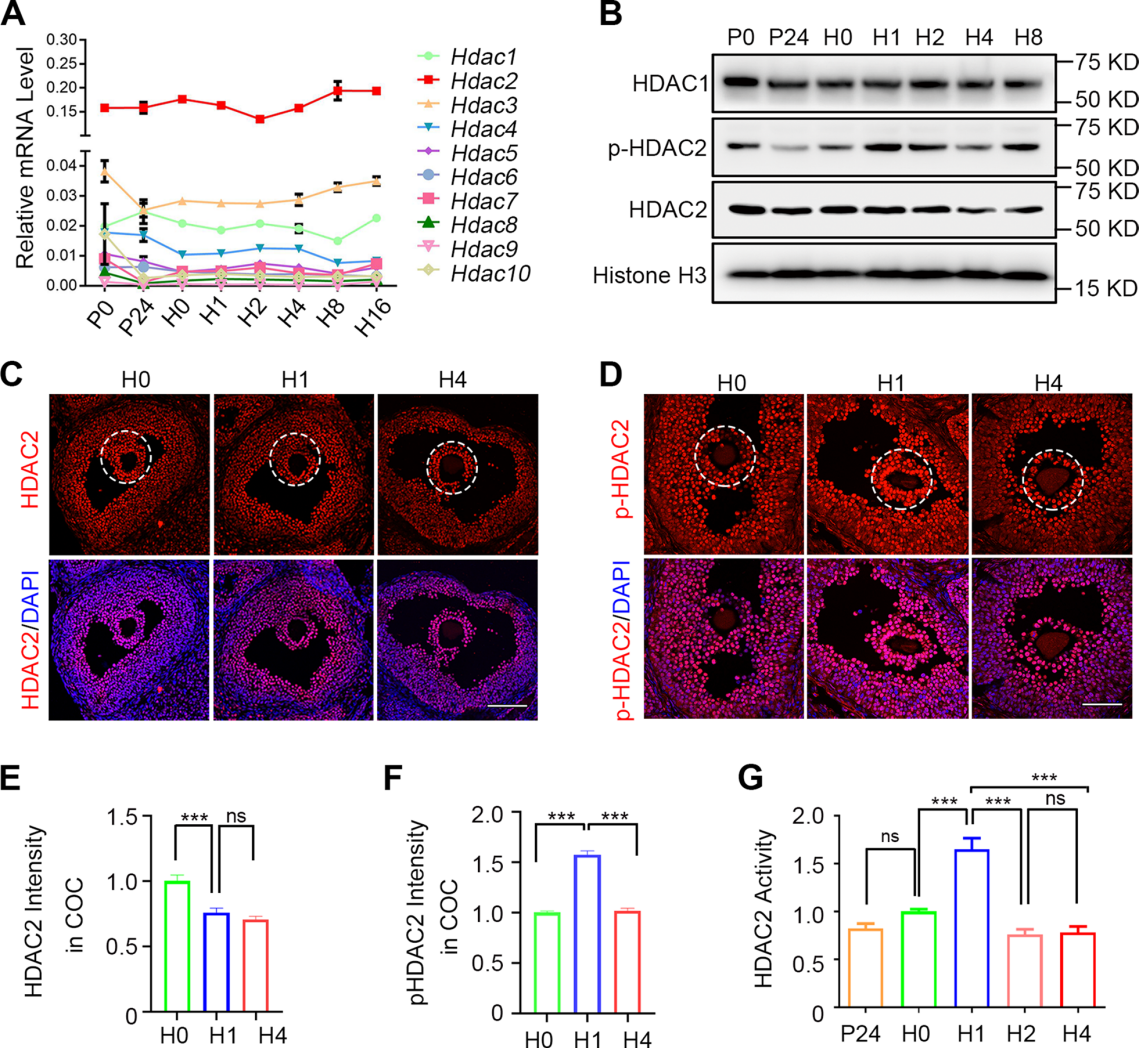
The Expression and Activity of HDAC2 at the Initial Stage of Ovulation. **A** RT-qPCR results demonstrate the mRNA expression levels of HDACs (*Hdac1-10*) in ovaries at different timepoints. **B** The protein levels of HDAC1, HDAC2, pHDAC2, and Histone H3 were determined by Western blotting in ovaries at different timepoints. P0, P24: 0 h and 24 h after PMSG treatment. H0–H8: 0, 1, 2, 4, and 8 h after hCG treatment followed by PMSG treatment for 48 h. **C**, **D** Representative immunofluorescence for HDAC2 (red in C) and pHDAC2 (red in D) in large antral follicles at different timepoints after PMSG and hCG treatment. Scale Bar = 100 μm. H0, H1, and H4: 0 h, 1 h, and 4 h after hCG treatment followed by PMSG treatment for 48 h. White dotted circles indicate cumulus cells and oocyte complex (COC). **E**, **F** Quantities of HDAC2 and pHDAC2 signal intensity in the COC in panels C and D. Data are presented as the mean ± SD (two-tailed unpaired t-test). **G** The levels of HDAC2 activity in GCs at different timepoints were determined using an in vitro assay kit. P24: 24 h after PMSG treatment. H0–H4: 0, 1, 2, and 4 h after hCG treatment, followed by PMSG treatment for 48 h. All data are presented as the mean ± SD. Two-tailed unpaired t-tests were performed for experiments depicted in panels E–F. One-way ANOVA was performed, followed by Turkey’s multiple comparisons test for experiments depicted in panel G. NS, no significance, *P < 0.05, **P < 0.01, ***P < 0.001

Next, we determined which HDACs mediate rapid histone deacetylation after hCG stimulation. Quantitative real-time PCR (RT-qPCR) was performed to examine the mRNA levels of all HDACs (HDAC1–HDAC10) and identified that HDAC2 mRNA levels were the highest in mouse ovaries at different stages (Fig. 3A). The protein levels of both HDAC1 and HDAC2 remained unchanged after PMSG or hCG treatment (Fig. 3B, C, and Additional file 1: Fig. S3).

Studies have demonstrated that HDAC2 phosphorylation at Ser394 (pHDAC2(S394)) is responsible for corepressor cooperation and histone deacetylase activity [21]. However, pHDAC2(S394) levels markedly increased at 1 h after hCG treatment (Fig. 3B). Immunofluorescence revealed increased pHDAC2(S394) levels in ovarian follicles at 1 h after hCG injection compared with 0 and 4 h after hCG injection (Fig. 3C and D). Interestingly, the HDCA2 signal decreased in cumulus cells from 0 to 1 h after hCG treatment (Fig. 3C and E), whereas a more intense pHDAC2 (S394) signal was observed in the cumulus cells than in mural GCs (Fig. 3D and F). Meanwhile, the HDAC2 activity was analyzed using mural GCs at different timepoints after PMSG or hCG injection. The in vitro activity assay revealed the highest activity of HDAC2 at 1 h after hCG injection (Fig. 3G), which is consistent with the immunofluorescence result in Fig. 3D. These results thus indicate that rapid activation of HDAC2 phosphorylation activation is induced by LH/hCG stimulations.

### HDAC2 activity is required for histone deacetylation, active transcription, and cumulus cell expansion

**Fig. 4:**
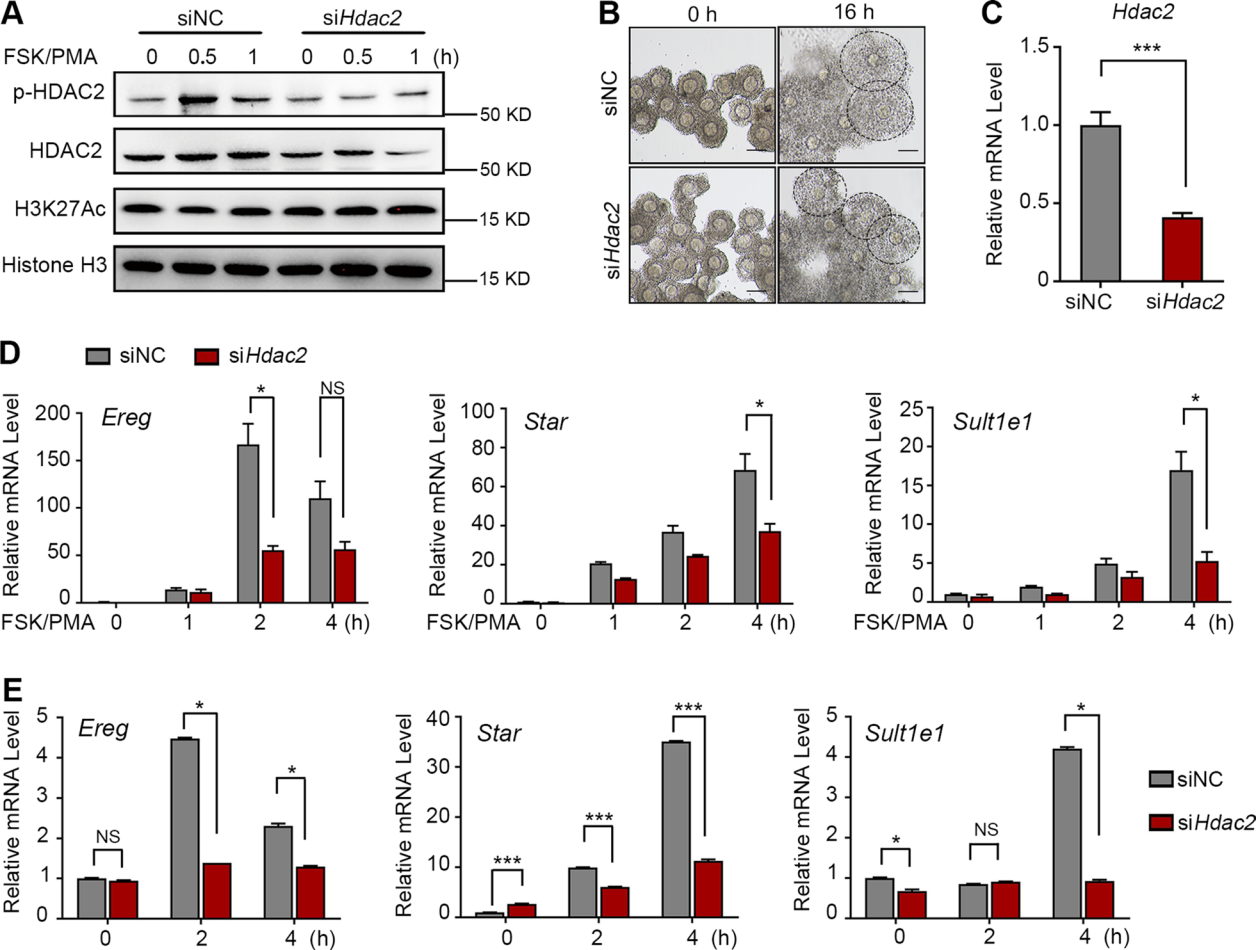
HDAC2 is Essential for Histone Deacetylation, Ovulation-specific Genes’ Transcription, and Cumulus Expansion. **A** Western blotting results demonstrating the dynamics of pHDAC2 and the increased H3K27Ac level with the transfection of *Hdca2* siRNAs in mouse primary GCs. Primary GCs were transfected with siRNAs against negative control (siNC) or *Hdac2* (si*Hdac2*) for 24 h, followed by the addition of 10 uM Forskolin (FSK) and 20 nM PMA to activate the ovulatory signal. **B** The representative images show the cumulus cell expansion capacity in the si-NC and si-*Hdac2* groups. **C** The *Hdac2* mRNA level was determined by RT-qPCR in cumulus cells derived from COCs of the si-NC and si-*Hdac2* groups. The data are presented as the mean ± SD with a *t*-test on log-transformed values. ***P < 0.001. **D** Quantification of the indicated ovulatory specific genes expression in primary GCs by RT-qPCR following ovulatory signal in the si-NC and si-*Hdac2* groups. **E** The RT-qPCR results for ovulatory specific genes’ mRNA levels in COCs from the si-NC and si-*Hdac2* groups. The data are presented as the mean ± SD. One-way ANOVA followed by Turkey’s multiple comparisons test on log-transformed values. NS, no significance, *P < 0.05, ***P < 0.001, ANOVA, analysis of variance. P0, P24, H0: 0 h, 24 h, and 48 h after PMSG treatment. H1–H8: 1, 2, 4, and 8 h post-hCG treatment

To further determine the influence of HDAC2 on histone deacetylation and subsequent transcription during ovulation, we transfected a negative control (NC) small-interfering RNA (siRNA) sequence or a siRNA targeting mouse *Hdac2* into primary cultured GCs and cumulus–oocyte complexes (COCs). We used forskolin (FSK) and phorbol 12-myristate 13-acetate (PMA) to mimic LH- or hCG-induced activation of the cAMP/PKA and PKC signaling pathway in primary cultured ovarian GCs [26, 27]. At 0.5 h following FSK/PMA treatment, HDAC2 phosphorylation, and histone deacetylation were observed in control GCs (Fig. 4A and Additional file 1: Fig. S4A). Following *Hdac2* siRNA transfection, HDAC2 protein levels decreased, and consequently, H3K27Ac could not be efficiently deacetylated (Fig. 4A; Additional file 1: Fig. S4A and B).

HDAC2 was highly expressed in cumulus cells and was phosphorylated after hCG induction (Fig. 3D). Because cumulus cell expansion of COCs is vital for successful ovulation, the role of HDAC2 in COC expansion was assessed. COCs were transfected with the NC and *Hdac2* siRNAs and subjected to in vitro maturation. *Hdac2* mRNA levels were significantly decreased and the cumulus cell expansion capacity was impaired compared with that in the control group (Fig. 4B, C; Additional file 1: Fig. S4C). In primary GCs and COCs of the control group, remarkably higher transcription levels of LH-induced ovulation-specific genes, such as *Ereg*, *Sult1e1,* and *Star*, were observed at 2–4 h after FSK/PMA treatment or after in vitro maturation (Fig. 4D and E). By contrast, the transcription of these genes was significantly compromised after HDAC2 depletion (Fig. 4D and E).Together, these findings indicate that HDAC2 depletion in GCs affects H3K27 deacetylation, subsequent ovulation-specific gene transcription, and cumulus cell expansion.

**Fig. 5:**
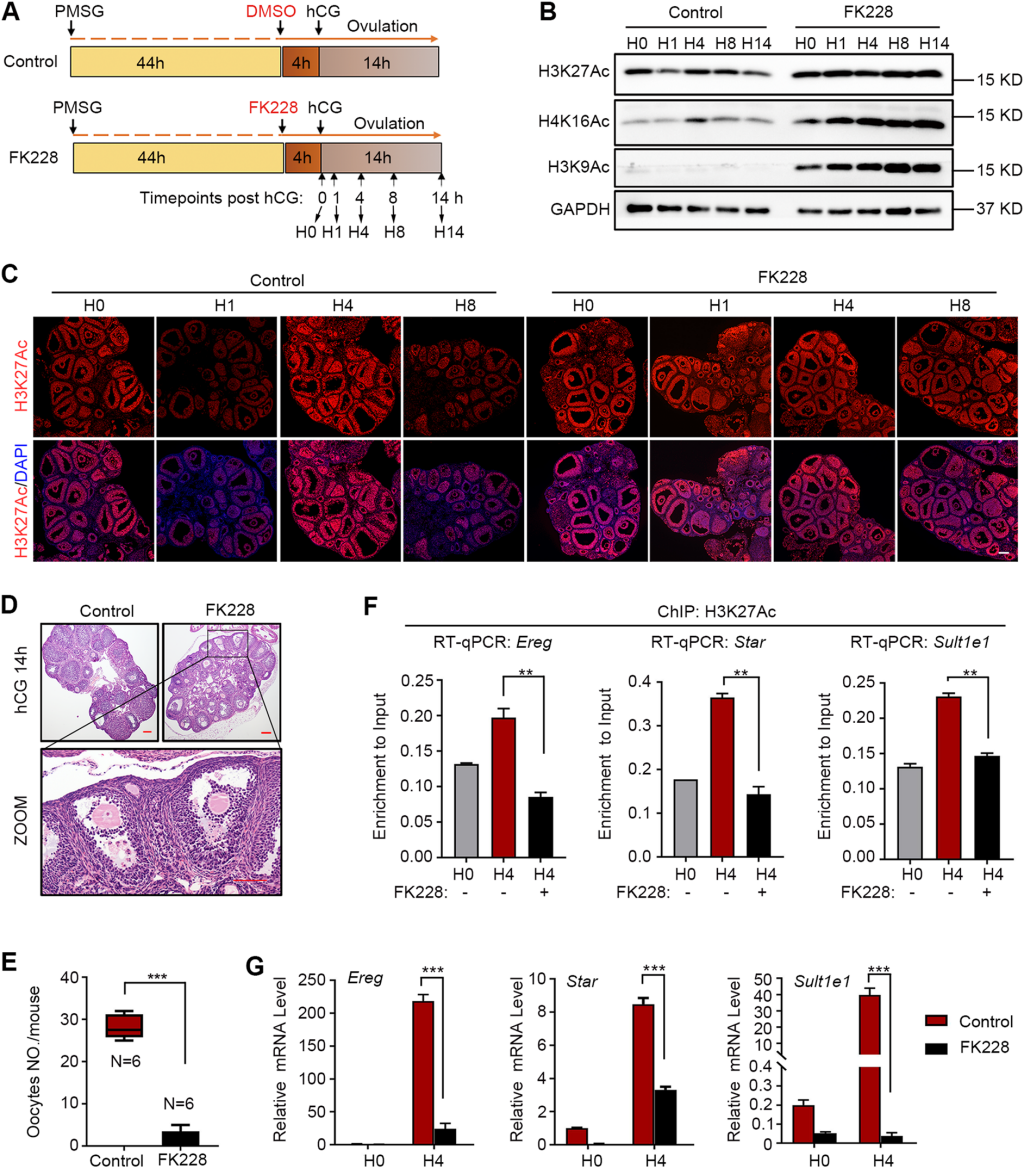
HDAC2-mediated Deacetylation is Essential for Transcription Switching and Successful Ovulation. **A** A diagram depicting the experimental design. FK228, the inhibitor of HDAC1/2, was pretreated for 4 h before hCG injection to induce ovulation. Black arrows indicate the time points when the ovaries were collected. H0–14: 0 h, 1 h, 4 h, 8 h, and 14 h after hCG treatment. **B** Western blotting of the main acetylated histones at different timepoints post-hCG in the control and FK228 groups. **C** Immunofluorescence of H3K27Ac at different timepoints (0, 1, 4, and 8 h) after hCG treatment in the control and FK228 groups. Scale bar = 100 μm. **D** The ovary morphology with hematoxylin and eosin staining from the control and FK228 groups at 14 h post-hCG. **E** The average oocyte numbers ovulated per mouse. The results are presented as the mean ± SD. ***P < 0.001, calculated by *t*-test. **F** ChIP-qPCR analysis of H3K27Ac on *Ereg*, *Star*, and *Sult1e1* at 0 h and 4 h post-hCG pre-treated with or without FK228. **G** The relative mRNA levels by RT-qPCR of *Ereg*, *Star*, and *Sult1e1* at 0, 4, and 8 h post-hCG in the control and FK228 groups. The fold change was represented by setting the relative level of 0 h in the control group as 1. The results (panels **F** and **G**) are presented as the mean ± SD. *P < 0.05, **P < 0.01, as calculated by one-way ANOVA, followed by Turkey’s multiple comparisons test on log-transformed values

To determine whether HDAC2 is essential for ovulation in vivo, mice were pretreated with DMSO or FK228 (a specific inhibitor of HDAC1 and HDAC2) for 4 h before hCG injection. We then analyzed H3K27Ac, transcription, and ovulation at different timepoints (Fig. 5A). H3K27Ac was deacetylated at 1 and 8 h after hCG treatment in the control group. However, the H3K27Ac level remained high after FK228 treatment at 1 h to 8 h after hCG treatment (Fig. 5B and C). In addition to H3K27Ac, H3K9Ac, and H4K16Ac were also retained at 1 h after hCG treatment in the FK228 group (Fig. 5B, C; Additional file 1: Fig. S4D). Ovaries from the control mice had many ruptured follicles, whereas many oocytes in the FK228 group were entrapped in the large antral follicles with their surrounding cumulus cells unexpanded and germinal vesicles intact (Fig. 5D and Additional file 1: Fig. S5). Consistent with these findings, the FK228-treated mice ovulated much fewer oocytes than the control mice at 14 h after hCG treatment (Fig. 5E). Using ChIP-qPCR, we found that H3K27Ac was not efficiently established at the ovulation-specific genes *Ereg*, *Star,* and *Sult1e1* at 4 h after hCG treatment in the FK228 group (Fig. 5F). Transcription of these genes was high at 4 h after hCG injection in the control group and significantly decreased after FK228 treatment (Fig. 5G). These results show that HDAC1/2 is required for hCG-induced ovulation, especially for cumulus cell expansion.

### CK2α is required for LH/hCG-induced HDAC2 phosphorylation, transcription, and ovulation

**Fig. 6:**
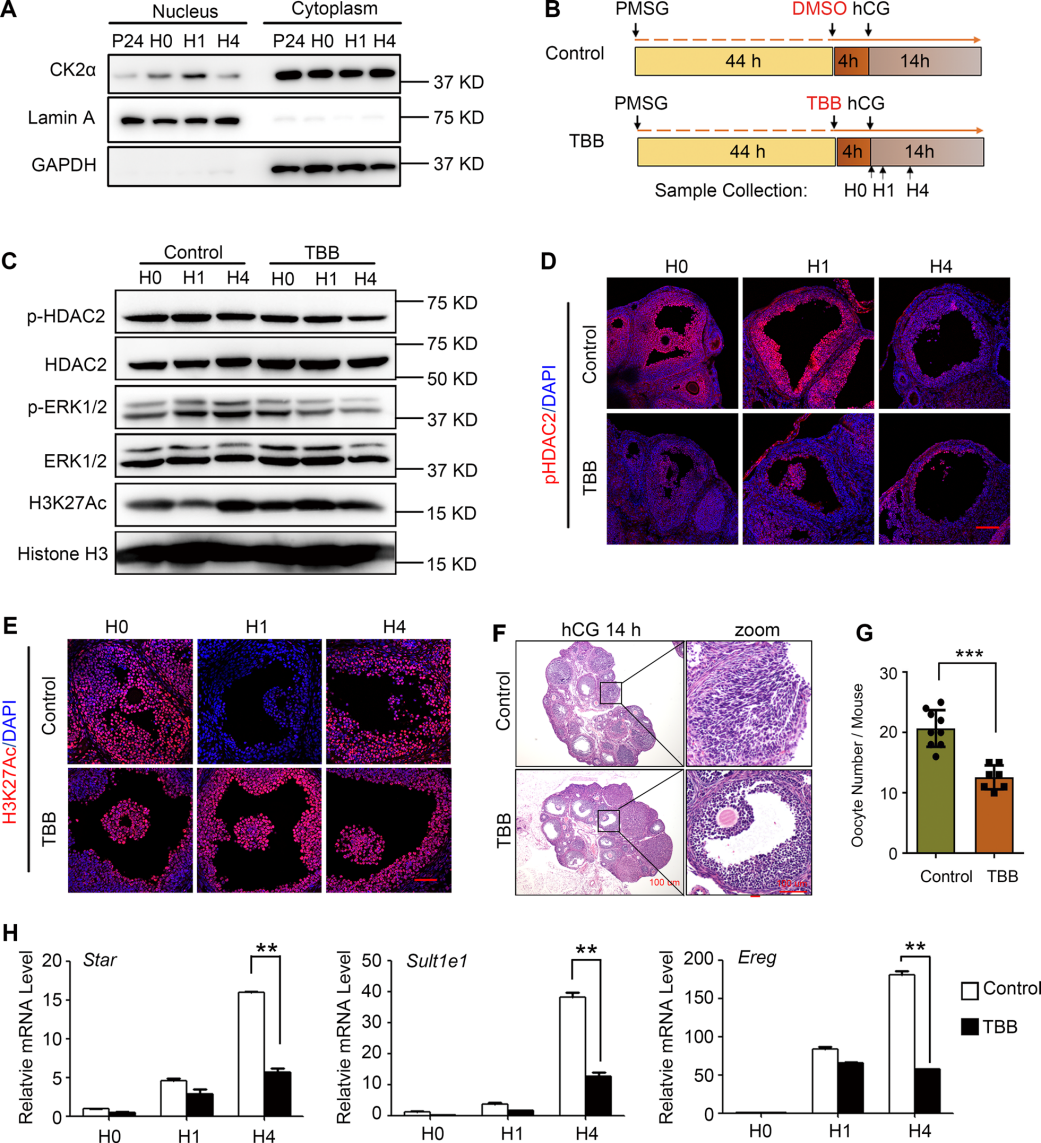
CK2α Nuclear Translocation is Required for HDAC2 Phosphorylation, H3K27Ac Deacetylation, and Ovulation. **A** Western blotting of CK2α in nuclear and cytoplasm fragmentation of ovaries at the indicated timepoints of PMSG or hCG. **B** A diagram depicting the experimental design. TBB is an inhibitor of CK2α. H0–H4: 0, 1, and 4 h after the hCG treatment. **C** Western blotting analysis for H3K27Ac, HDAC2, pHDAC2, ERK1/2, and pERK1/2 at different timepoints (0, 1, and 4 h) post-hCG in the control and TBB groups. **D**, **E** Immunofluorescence of pHDAC2 (D, red) and H3K27Ac (E, red) at different timepoints (0, 1, and 4 h) after hCG treatment in the control and TBB groups. Nuclei were co-stained with DAPI (n = 3). Scale bar = 100 μm. **F** Hematoxylin and eosin staining showed that the TTB treatment inhibits cumulus expansion and ovulation. The mice ovaries were collected at 14-h post-hCG in mice of the control and TBB groups (n = 9). Scale bar = 100 um. **G** The bar graph depicting the ovulated oocyte numbers from mice in the control and TBB groups (n = 9). Data are presented by the mean ± SD. *P* value was determined by* t*-test. ***P < 0.001. **H** RT-qPCR analysis of the mRNA levels of ovulatory specific genes, including *Ereg*, *Star*, and *Sult1e1*. The fold change was represented by setting the relative level of 0 h in the control group as 1. The results are represented as mean ± SD. *P < 0.05, **P < 0.01, calculated by one-way ANOVA followed by Turkey’s multiple comparisons tests

We previously demonstrated that ERK1/2 phosphorylation activation is involved in active histone acetylation at 4 h after hCG treatment by enhancing CBP/P300 activity [10]. However, the other signal pathway involving ovulation and histone remodeling is less investigated. Because the protein kinase CK2α phosphorylates HDAC2 [20], we determined CK2α translocation after hCG treatment. Specifically, CK2α was clearly and transiently translocated to the nucleus at 1 h after hCG treatment (Fig. 6A and Fig. S6). At 44 h after PMSG treatment, the mice were pre-treated with TBB (a CK2α inhibitor) for 4 h and then injected with hCG to induce ovulation (Fig. 6B). Interestingly, ERK1/2 phosphorylation was reduced from 1 to 4 h after hCG injection in the TBB group (Fig. 6C and Fig. S6). CK2α inhibition significantly attenuated HDAC2 phosphorylation levels (Fig. 6C, D and Fig. S6) and blocked the decrease in H3K27Ac at 1 h after hCG treatment (Fig. 6E). At 14 h after hCG injection, cumulus cell expansion was inhibited and fewer mature oocytes were ovulated in the TBB group than in the control group (Fig. 6F and G). Transcription of ovulation-specific genes, such as *Star*, *Sult1e1,* and *Ereg*, was also reduced (Fig. 6H). Together, these results indicate that LH/hCG-induced CK2α nuclear translocation promotes HDAC2 phosphorylation, ovulatory gene transcription, and ovulation.

## Discussion

**Fig. 7:**
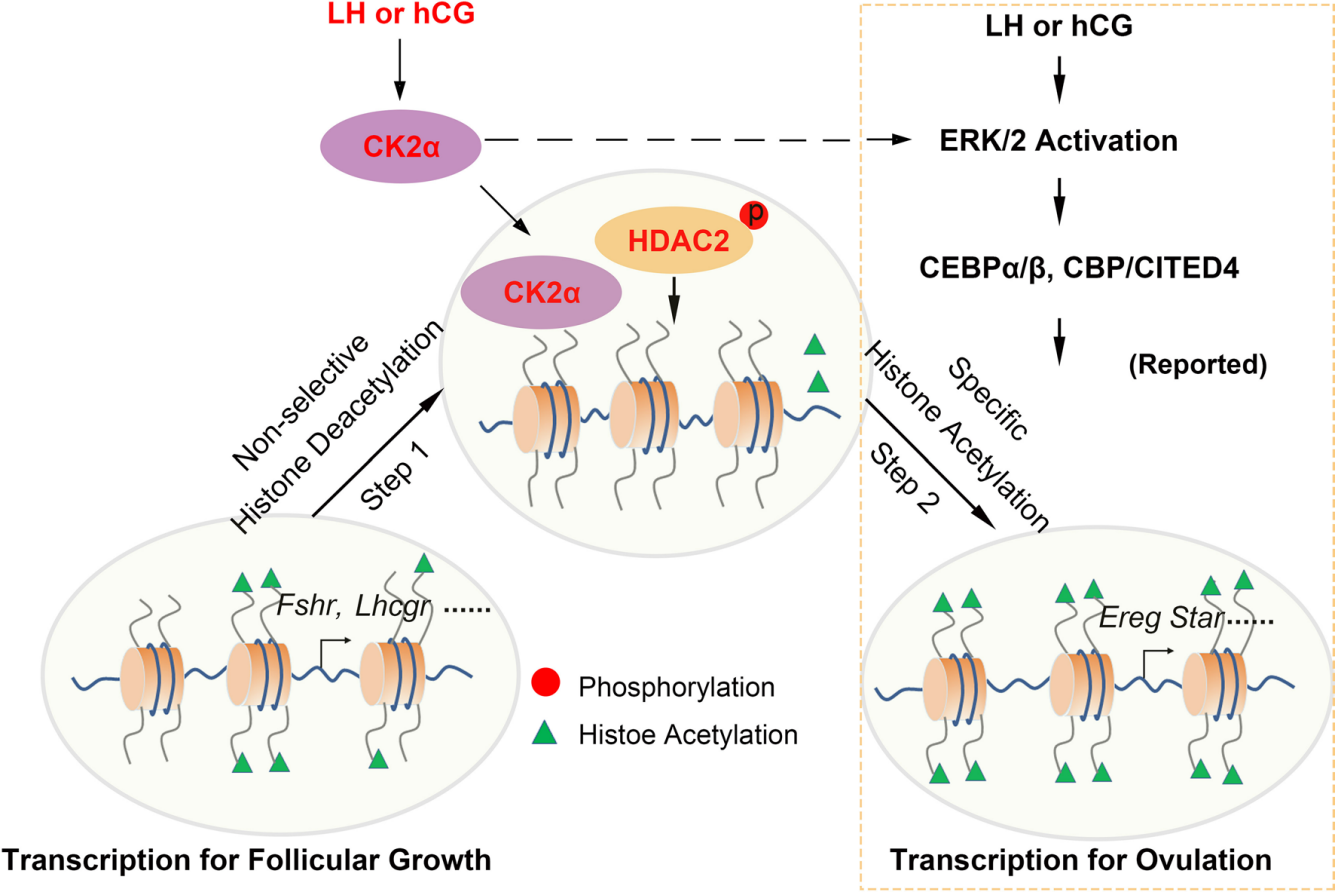
Proposed Chromatin Remodeling Model for LH/hCG-induced Ovulation. During the antral follicle growth, the GCs display high levels of histone acetylation catalyzed by histone acetylation transferases (HATs) as well as active transcription in response to FSH. Once LH surge or hCG trigger, the nuclear translocation CK2α enhances HDAC2 phosphorylation, thereby deacetylating histones non-selectively. Meanwhile, LH/hCG induces ERK1/2 phosphorylation activation to induce histone acetylation by HATs at ovulation-specific genes

Physiologically, ovarian GCs mediate the action of FSH on follicular growth and that of LH or hCG on ovulation in preovulatory follicles. LH/hCG-mediated genetic reprogramming drives the cell fate transition from proliferating follicular GCs to terminally differentiated lutein cells, thereby leading to distinct gene expression patterns [11]. We here defined a new state of chromatin remodeling required for HDAC2-mediated histone deacetylation. LH/hCG induction promotes CK2α nuclear translocation to phosphorylate HDAC2 for histone deacetylation. Meanwhile, CK2α also facilitates activation of the ERK1/2 signal cascade, which is essential for specific histone acetylation of ovulation (Fig. 7).

H3K27Ac is a well-defined marker of the active promoter and enhancer [28], and convincing evidence has confirmed that H3K27Ac precedes active transcription [12, 13]. Most studies on GCs have focused on histone modifications on specific genes, such as total histone H3 acetylation on *Star* [29] and H3K27Ac on *Amh* [30]. A recent ChIP-seq study on the progesterone receptor (PGR) and H3K27Ac demonstrated that PGR-binding sites in GCs are highly enriched in proximal promoter regions close to the H3K27Ac-modified active chromatin during ovulation [31]. Because CBP/P300 acetylates H3K27Ac [32], CBP/P300 inhibition in GCs was found to block LH-induced histone acetylation [10], which indicated a low level of histone acetylation in preovulatory follicles at the initial stage of LH induction. Therefore, we speculate that histone deacetylation occurs between the follicle state transition. Expectedly, we found that FSH and LH simultaneously stimulated active transcription and strong histone acetylation in GCs (Fig. 1B and D), and several histone acetylation modifications (H3K27Ac, H3K9Ac, and H4K16Ac) were rapidly erased after hCG treatment. Our finding is consistent with the study conducted using quantitative acetylomics analyses. That study demonstrated a subset of CBP/p300-regulated sites (including histones) with very rapid (< 30 min) acetylation and deacetylation kinetics [32]. In contrast to the prior study, which used GCs at 6 h after hCG treatment, we collected ovaries at 0, 1, and 4 h, during the initial ovulation stage. Our H3K27Ac ChIP-seq results not only revealed a new style of histone dynamics in preovulatory follicles after hCG induction but also characterized H3K27Ac binding profiles on the genome. This study further demonstrated that H3K27Ac poises a subset of genes with low expression in preovulatory follicles, and the ovulatory signal induces these gene expression dramatically through a transient H3K27 deacetylation and a subsequent stronger H3K27Ac landscape establishment.

LH or hCG mediates the transcription of many ovulation-induced genes, such as EGF-like factors (*Areg*, *Ereg,* and *Btc*), *Star*, *Sultl1e1,* and *Ptgs2* [8]. The success of ovulation depends on the follicle state before the LH surge and subsequent activation of the LH-induced PKA and ERK1/2 signal pathway for ovulatory gene transcription. This activation is induced by promoting C/EBPα and C/EBPβ expression for ovulation-specific gene expression [8, 9]. Upregulation of these genes at 4 h after hCG treatment compared with 0 h is consistent with the reported microarray and RT-qPCR results [9, 33, 34]. We here found that H3K27Ac poised to ovulation-induced genes for their active transcription after hCG induction. Many of these genes were related to MAPK1/3 (or ERK1/2) and VEGF signaling pathways (Fig. 2L), which play vital roles during ovulation and luteinization [35, 36]. SMAD3 was predicted as a transcription factor for H3K27Ac establishment. SMAD3 cooperates with SMAD2 to initiate transcription, thereby recruiting and binding with CBP/P300 in GCs [30]. Moreover, our study using a mouse model demonstrated that GC-specific deletion of *Smad4*, encoding a binding partner of SMAD2/3, led to defects in ovulation [37]. Other transcription factors, such as TBP and GRHL2, were predicted based on the motif analysis. TBP is a critical transcription factor required for promoter recognition and assembly of transcription pre-initiation complexes [38]. GRHL2 acts as an essential transcription factor for epithelial-to-mesenchymal transitions and estrogen-induced transcription in breast cancer [39, 40]. A recent study showed that H3K27Ac-enriched genes harbor a putative GRHL transcription factor-binding site in psoriasis [41]. However, the roles of TBP and GRHL2 in ovaries remain unknown. Further studies need to be conducted to investigate the GC specificity of H3K27Ac establishment and active transcription initiation during ovulation.

HDACs are divided into four classes [42], and class I HDACs (HDAC1, 2, 3, and 8) have attracted interest as therapeutic targets for cancer and some other diseases [43, 44]. They are known to play key roles in maintaining ovarian function. HDAC1 and HDAC2 are highly homologous class I HDACs and are found in large multimeric complexes (Sin3, NuRD, and CoREST). They can catalyze the removal of acetyl groups from histone tails [45]. NuRD-mediated removal of H3K27Ac facilitates H3K27me3 in stem cells [46]. Only one study has reported that the HDAC1/HDAC2/Sin3A complex has crucial roles in the repression of *LHCGR* transcription in ovaries [47]. The Xia group recently found that the expression of some ovulatory genes (such as *Areg*) cannot be initiated before the ovulatory signal because HDAC3 blocks the accessibility of the *Areg* promoter [48]. The completely different expression patterns of HDAC1/2 and HDAC3 may contribute to their distinct functions during follicular development and ovulation.

CK2α is a kinase phosphorylating HDAC1 and HDAC2 and thus facilitating their functions [22]. In PCOS patients with ovulation dysfunction, CK2α protein levels were increased in luteinized GCs in a previous study [23]. CK2α binds with the androgen receptor (AR) to phosphorylate and stabilize AR activation, thereby forming a vicious cycle of constant androgen-AR-CK2α activation [23]. After treatment with TBB, a CK2α inhibitor, the number of oocytes decreased in the TBB group compared with the control group (Fig. 6F and G). This study demonstrated nuclear translocation of CK2α to phosphorylate HDAC2 is essential for ovulation, which may explain the mechanism of androgen-AR-CK2α axis-induced anovulation in PCOS patients.

Interestingly, after hCG induction, both HDAC2 and pHDAC2 preferentially localized in cumulus cells and mural GCs adjacent to the follicular fluid (Fig. 3C). This possibly resulted from high EGF-like factors in these areas. *Hdac2* depletion or inhibition leads to inactive transcription of ovulation-specific genes and abnormal cumulus expansion. These results indicate that HDAC2 has a more crucial role in cumulus GC expansion. HDAC2 is a gene repressor, but many studies have shown that the HDAC2 level is associated with the transcription level [18, 49]. We suggest that active transcription is required for the rapid histone acetylation and deacetylation balance. Meanwhile, HDAC1/2 plays roles in cell proliferation, migration, and development by deacetylating non-histone proteins, such as MyoD [50], E2F1 [51], STAT3 [52], and SMAD7 [53]. These proteins may cooperate with histones to facilitate gene transcription during hCG-induced ovulation. Further investigations about the mechanism of HDAC2 and active transcription in ovulation are warranted.

## Conclusions

This study demonstrates that, after ovulatory signal induction, non-selective histone deacetylation occurs to reset the chromatin, which is followed by specific histone acetylation for ovulation. HDAC2 phosphorylation activation-mediated histone deacetylation is essential for ovulation. The study provides a better understanding of the physiology of LH/hCG-induced ovulation.

## Materials and methods

### Mice

Female ICR mice were obtained from the Experimental Animals Center of Zhejiang Academy of Medical Sciences. Mice were housed in specific pathogen free conditions with regular and normal environment. All animal experiments were conducted according to the guidelines of the Animal Care Committee of Zhejiang University.

Ovarian stimulation was performed in 23- to 25-day-old female mice via intraperitoneal injection of 5 IU of PMSG (Ningbo Sansheng, China) to stimulate follicle growth followed primed with 5 IU of hCG (Ningbo Sansheng, China) 44–48 h later to induce ovulation.

For inhibitor administration in vivo, 23-day-old female mice were injected with saline, 2.16 mg/kg body weight FK228 (HY-15149, MCE, USA) or 50 mg/kg body weight TBB (HY-14394, MCE, USA) dissolved in saline at 44 h post PMSG injection and then with 5 IU of hCG 4 h later. Ovaries were collected 0 h, 1 h, 4 h, 8 h, and 14 h post hCG injection for frozen sectioning, mRNA extraction and western blotting. Meanwhile, MII oocytes were collected for counting at 14 h after hCG.

### Primary cell cultures and siRNA transfection

To culture primary COCs and GCs, mice at 3 weeks of age were sacrificed 48 h after injection of 5 IU of PMSG, as described previously [10]. COCs and GCs were acquired by puncturing ovarian antral follicles with a needle. Primary GCs were cultured in DMEM/F12 (Gibco) with 5% (v/v) fetal bovine serum (FBS; Gibco) and 1% (v/v) penicillin and streptomycin at 37℃ with 5%CO_2_. To knock down HDAC2 expression, GCs were transfected with *Hdac2* siRNAs (si-*Hdac2*) or negative control siRNA (si-NC) with Lipofectamine™ 3000 Transfection Reagent (L3000015; Thermo Scientific, USA) in accordance with the manufacturer’s instructions. The sequences of si-*Hdac2* are summarized in Additional file 2: Table S2, and the siRNAs were synthesized by RiboBio Co., Ltd. (China). The COCs were transfected with negative control or *Hdac2* siRNAs for 24 h in α-MEM (GIBCO, USA) medium, supplemented with 26 mM sodium bicarbonate, 0.23 mM pyruvate, 3 mg/ml BSA, 2.5 uM milrinone and 1% (v/v) penicillin and streptomycin (Meilunbio, China), and then were cultured in a commercial in vitro maturation medium (EasyCheck Company, China) for 14 h at 37 ℃ with 5% CO_2_.

To mimic the effects of LH, GCs were treated with FSK (10 μM; MCE, USA) and PMA (20 nM; MCE, USA), as described previously [34], for 30 min, 1 h, 2 h and 4 h. FK228 (100 nM) or U0126 (10 µM; #9903, CST, USA) was added, and the GCs were incubated with these reagents for 12 h before FSK/PMA treatments. For analysis the effect of hyperandrogenism, 10 uM testosterone was added in mouse primary GCs for different times combined with 5uM or 10 uM FK228 or transfection of siRNAs.

### Western blot (WB)

Proteins were extracted from tissues and cells dissolved in 1 × Laemmli sample buffer (1610747, Bio-Rad), and then resolved by SDS-PAGE after boiling at 95℃ for 10 min. After the proteins were transferred onto polyvinylidene difluoride membranes (Millipore Corp., USA), the membranes were blocked in 5% defat milk for 1 h at room temperature, and then incubated with primary antibodies overnight at 4 °C. After 3 times washing in TBS/0.1%Tween-20, the membranes were then incubated with horseradish peroxidase-conjugated anti-rabbit or anti-mouse IgG secondary antibodies (111-035-003 or 115-035-003; Jackson Immuno Research, USA) for 1 h at room temperature. The bound proteins were visualized by incubating enhanced chemiluminescence (WBKLS0500; Millipore Corp., USA). The primary antibodies are listed in Additional file 2: Table S3.

### RNA isolation and RT-qPCR

Total RNA was isolated from mouse ovaries and GCs with TRIzol Reagent (Invitrogen, USA). RNA was extracted from COCs using RNeasy Mini Kit (74104; Qiagen, Germany) in accordance with the manufacturer’s protocol. Reverse transcription was conducted with HiScript II Reverse Transcriptase (R201-1; Vazyme, China). RT-qPCR was performed with SYBR qPCR Master Mix (Q511-02; Vazyme, China) on an CFX96 Real-time System (Bio-Rad, USA). The relative mRNA expression levels were normalized to the endogenous *Gapdh* mRNA levels and compared with those in the controls, and all RT-qPCR assays were performed in triplicate. The primers are shown in Additional file 2: Table S2.

### Immunofluorescence

Ovaries were collected, fixed in 4% (w/v) paraformaldehyde and embedded in paraffin under routine procedures. For histology, the ovaries were then sectioned at 5 μm thickness and stained with haematoxylin and eosin (H&E). To perform IF, the tissues were embedded in optimal cutting temperature (OCT) compound and sectioned at 5 μm thickness. The sections were incubated for 1 h with blocking buffer (PBS containing 5% bovine serum albumin (w/v) and 0.3% Triton X-100 (v/v)). After being probed with primary antibodies at 4 °C overnight, the sections were incubated with Alexa Fluor 568-conjugated goat anti-rabbit IgG (H + L) secondary antibodies (dilution: 1:500; A-11036, Thermo Scientific, USA) for 1 h and counterstained with 4′,6-diamidino-2-phenylindole (DAPI; 1 µg/ml; Roche, Switzerland) for 10 min. The primary antibodies are summarized in Additional file 2: Table S3. Finally, images were obtained under a confocal microscope (LSM800, Carl Zeiss, Germany). We used the same experimental conditions (e.g., fixation time and antibody dilution) and picture capture parameters for every group in each independent experiment.

### HDAC2 activity assay

HDAC2 activity was measured with HDAC-Glo™ I/II Assays (G6420; Promega, USA) according to the manufacturer’s protocol. Briefly, 100,000 cells were mixed with HDAC-Glo™ I/II Reagent at room temperature for 30 min, and HDAC2 activity was determined by luminescence.

### ChIP-seq

As H3K27Ac is mainly deposited in GCs of the ovaries, ChIP was performed using whole ovary samples with the SimpleChIP Plus Enzymatic Chromatin IP Kit (#9005, CST, USA) in accordance with the manufacturer’s instructions. Briefly, the ovaries were collected 0, 1, and 4 h post-hCG treatment (approximately 25 mg of the ovarian tissues per immunoprecipitation (IP) experiment) and sliced into small pieces in cold PBS containing a protease inhibitor cocktail and cross-linked with a final concentration of 1.5% formaldehyde in PBS at room temperature. After the addition of glycine to stop cross-linking and two washes in PBS, the tissues were disaggregated into single-cell suspensions by using a high-speed tissue homogenizer. The nuclei were prepared using the provided buffer A (ice-cold) with a protease inhibitor cocktail. Chromatin was digested in 0.5 µL of Micrococcal Nuclease (#10,011, CST) and then sonicated using a Bioruptor apparatus (Diagenode, Belgium) at a high intensity for 40 cycles of 10-s ON and 10-s OFF on an ice bath. Then, 1/10 dilutions of the lysates were purified for DNA and subjected to electrophoresis and concentration determination. For each sample, 2% of the remaining volume was kept aside as the input. ChIP was performed on approximately 10 µg of the digested and cross-linked chromatin. The chromatin for each timepoint sample was incubated with 1 µg of histone H3 antibody (as a positive control; #4620, CST), 1 µg of normal rabbit IgG (as a negative control; #2729, CST), or 1 µg of H3K27Ac antibody (ab177178, Abcam, USA) overnight at 4 °C and then with 40 µL of protein G magnetic beads per IP incubation for 2 h at 4 °C with rotation. After washing, elution, and reversal of cross-linking, DNA was purified in the provided columns. The libraries were prepared for the ChIP DNA experiments by using the TruSeq DNA LT/HT Sample Prep Kit (Illumina) and subjected to quality control on the Bioanalyzer (Agilent 2200). Illumina sequencing was performed on the Illumina HiSeq 2000 Sequencing system at RiboBio Co., Ltd.

### ChIP-seq data analysis

The ChIP-seq reads were aligned to the mouse genome mm10 using Bowtie (Version 2.0) under default parameters. For peak calling, we used MACS2.0 by applying default parameters and significance threshold (q value cutoff = 0.05), as described previously [54]. To visualize the ChIP-seq data, deepTools2 was used [55]. Briefly, BAM coverage was used to generate the bigwig files. Then, a computed matrix and heatmap were employed to calculate and visualize the H3K27Ac heatmap and density profiles among the samples. The genome tracks were visualized in the software program IGV [56]. Peak annotation was performed using R and the ChIPseeker package [57]. Bedtools was employed to analyze the overlapping regions in different samples [58]. To identify motifs enriched in H3K27Ac ChIP peaks, Homer’s motif analysis (findMotifsGenome.pl) was used with the inclusion of known motifs and de novo motifs [59]. The GO analysis and KEGG pathway enrichment analyses were conducted with the Database for Annotation, Visualization, and Integrated Discovery (DAVID) (http://david.abcc.ncifcrf.gov/home.jsp) using a cut-off *P* < 0.05. The GO results were visualized using the bioinformatics online website (http://www.bioinformatics.com.cn/).

### ChIP-qPCR

For ChIP-qPCR, 25 mg ovaries were used per reaction. ChIP was performed as described above. Then, qPCR was carried out the to determine the enrichment of antibodies of IgG or H3K27Ac using DNA of input and ChIP eluents.

The RNA-seq data for mouse granulosa cells at 0 h and 4 h after hCG treatment were retrieved from the GEO (accession number: GSE119508), and the upregulated genes after hCG induction were extracted from the supplementary data in their article. The intensities of the WB bands and fluorescence images were analysed with ImageJ software (National Institutes of Health, USA). The RT-qPCR data, WB and IF intensities, HDAC2 activity and oocyte superovulation numbers are presented as the means ± SDs. The results were analysed with GraphPad Prism (Version 7.0, GraphPad Software, USA). Multiple groups were compared using one-way ANOVA without matching or pairing followed by Tukey’s post-test. Two groups were compared by two tailed unpaired *t*-test. A *P*-value < 0.05 was considered statistically significant.

## Supplementary Information

**Additional file 1.** **Figure S1. **The Dynamics of H3K27Ac Levels during Follicular Growth and Ovulation. Mice were treated with pregnant mare serum gonadotrophin (PMSG) to stimulate follicle growth or human chorionic gonadotropin (hCG) to trigger ovulation. Ovaries were collected at the indicated timepoints for immunofluorescence. The representative images showing the H3K27Ac (red) levels with DAPI (blue) co-stained for visualization of nucleus (n=3). Scale bar=100um. **Figure S2.** The H3K27Ac ChIP-seq Analysis for H3K27Ac-gain Genes after Ovulation Signal Induction. **(A)** Pie charts represent the ratio of stable H3K27Ac-enriched peaks, H3K27-loss peaks and H3K27-gain peakss (*de novo* H3K27Ac-deposited peaks and H3K27Ac-increased peaks). **(B) **Genome browser snapshot shows *Prss56* is one of *de novo* H3K27Ac-gain genes after hCG induction. **(C) **Gene Ontology (GO) analysis shows the biological process (BP) of *de novo* H3K27Ac-gain genes after hCG induction.** (D)** Bar charts representing the top enriched KEGG pathway of the ovulatory specific genes 4 h post hCG. **(E) **The HOMER known and *de novo* motif analysis for the H3K27Ac-gain peaks to predict transcriptional factors. **Figure S3.** Semiquantitative analysis of protein levels in Fig. 3. The western blot band intensities of Fig. 3B were measured with ImageJ software. HDAC1 and p-HDAC2 protein levels were normalized to Histone H3 levels. Data were expressed as mean±SD. *P* value was determined by two-way ANOVA followed by Tukey’s post-test. ** *P*<0.01.**Figure S4.** Depletion or inhibition of HDAC2 Causes Increased H3K27Ac Levels and Affects COC expansion. **(A)** Western blot analysis and quantification of HDAC2 and H3K27Ac levels in siNC and si*Hdac2 *group. Primary granulosa cells were transfected of negative control (siNC) or *Hdac2 *siRNAs (si*Hdac2*) for 36 h followed by treatment of forskolin (FSK, 10 μM) and phorbol 12-myristate 13-acetate (PMA, 20 nM) for indicated time length. All the protein levels were normalized to Histone H3 levels. The HDAC2 bands’ intensities were compared with siNC and si*Hdac2* groups in C. The bands’ intensities of H3K27Ac were compared among FSK/PMA non-treated group (0h) and FSK/PMA treated groups (0.5h, 1h, 2h and 4h). **(B)** Semiquantitative analysis of p-HDAC2 and H3K27Ac levels in Fig. 4A. The western blot band intensities of Fig. 4A were measured with ImageJ software. All protein levels were normalized to Histone H3 levels. The si*HDAC2* bands’ intensities were compared with siNC bands’ intensities.** (C)** Comparation of COCs expansion areas in Fig. 4B. The areas of COCs expansion in siNC and si*HDAC2* groups were measured with ImageJ software. **(D)** Quantitative analysis of H3K27Ac, H3K9Ac and H4K16Ac levels in Fig. 5B. All the protein levels were normalized to histone H3 levels. The FK228 bands’ intensities were compared with control bands’ intensities. All data in A-D were expressed as mean±SD. *P* value was determined by two-way ANOVA followed by Tukey’s post-test. ns. means no significance. * *P*<0.05, ** *P*<0.01. **Figure S5. **Inhibition of HDAC1/2 Hinders Cumulus Expansion and Ovulation. Hematoxylin and eosin (HE) staining results showing FK228, a HDAC1/2 inhibitor, blocks cumulus expansion and cause follicle atresia. Mice pretreated PMSG for 44 h were injected with DMSO or FK228, and 4 h later followed by hCG injection to induce ovulation (n=12). Ovaries were collected at 0 h, 4 h and 8 h post-hCG in mice of control and FK228 group. Scale bar, 50 um. **Figure S6. **Semiquantitative analysis of CK2α, p-HDAC2, H3K27Ac and p-ERK1/2 levels in Fig. 6A and C. The western blot band intensities were measured with ImageJ software. CK2α, p-HDAC2 and H3K27Ac protein levels were normalized to Histone H3 levels and p-ERK1/2 levels were normalized to ERK1/2. The TBB bands’ intensities were compared with control bands’ intensities. Data were expressed as mean±SD. *P* value was determined by two-way ANOVA followed by Tukey’s post-test. ** *P*<0.01

**Additional file 2:** **Table S1.** Oligonucleotides used for qPCR or siRNA. **Table S2.** Reagents or commercial kits used in this study. **Table S3.** The DEG list of RNA-seq data (H4 vs H0).

## Data Availability

The H3K27Ac ChIP-seq data was deposited in NCBI Gene Expression Omnibus with the accession number GSE165809. The RNA-seq data of mouse granulosa cells at 0 h and 4 h after hCG were obtained from the previously published datasets (GSE119508). The differential expression genes (DEG) were directly obtained from the processing RNA-seq data as previously reported [11]. We also provided this DEG list in Table S3. All the data supporting the findings within this article is available from the corresponding author upon reasonable request.

## Abbreviations

LH: Luteinizing hormone
FSH: Follicle-stimulating hormone
hCG: Human chorionic gonadotropin
PMSG: Pregnant mare serum gonadotrophin
GCs: Granulosa cells
COC: Cumulus-oocyte complex
PCOS: Polycystic ovarian syndrome
AREG: Amphiregulin
EREG: Epiregulin
BTC: Betacellulin
C/EBPα/β: CCAAT/enhancer-binding protein α/β
NuRD: Nucleosome remodeling and deacetylase
HAT: Histone acetyltransferase
FSK: Forskolin
PMA: Phorbol 12-myristate 13-acetate
