# Supplementary material for: Ovulatory signal-triggered chromatin remodeling in ovarian granulosa cells by HDAC2 phosphorylation activation-mediated histone deacetylation
Source: Epigenetics Chromatin. 2023 Apr 19;16:11. doi: 10.1186/s13072-023-00485-8 (PMC10116676; doi:10.1186/s13072-023-00485-8)
Supplement: Supplementary file 1 — Additional file 1. Figure S1. The Dynamics of H3K27Ac Levels during Follicular Growth and Ovulation. Mice were treated with pregnant mare serum gonadotrophin (PMSG) to stimulate follicle growth or human chorionic gonadotropin (hCG) to trigger ovulation. Ovaries were collected at the indicated timepoints for immunofluorescence. The representative images showing the H3K27Ac (red) levels with DAPI (blue) co-stained for visualization of nucleus (n=3). Scale bar=100um. Figure S2. The H3K27Ac ChIP-seq Analysis for H3K27Ac-gain Genes after Ovulation Signal Induction. (A) Pie charts represent the ratio of stable H3K27Ac-enriched peaks, H3K27-loss peaks and H3K27-gain peakss (de novo H3K27Ac-deposited peaks and H3K27Ac-increased peaks). (B) Genome browser snapshot shows Prss56 is one of de novo H3K27Ac-gain genes after hCG induction. (C) Gene Ontology (GO) analysis shows the biological process (BP) of de novo H3K27Ac-gain genes after hCG induction. (D) Bar charts representing the top enriched KEGG pathway of the ovulatory specific genes 4 h post hCG. (E) The HOMER known and de novo motif analysis for the H3K27Ac-gain peaks to predict transcriptional factors. Figure S3. Semiquantitative analysis of protein levels in Fig. 3. The western blot band intensities of Fig. 3B were measured with ImageJ software. HDAC1 and p-HDAC2 protein levels were normalized to Histone H3 levels. Data were expressed as mean±SD. P value was determined by two-way ANOVA followed by Tukey’s post-test. ** P<0.01.Figure S4. Depletion or inhibition of HDAC2 Causes Increased H3K27Ac Levels and Affects COC expansion. (A) Western blot analysis and quantification of HDAC2 and H3K27Ac levels in siNC and siHdac2 group. Primary granulosa cells were transfected of negative control (siNC) or Hdac2 siRNAs (siHdac2) for 36 h followed by treatment of forskolin (FSK, 10 μM) and phorbol 12-myristate 13-acetate (PMA, 20 nM) for indicated time length. All the protein levels were normalized to Histone H3 levels. The HDAC2 [file 13072_2023_485_MOESM1_ESM.docx]

**Supplementary Figures and Legends**


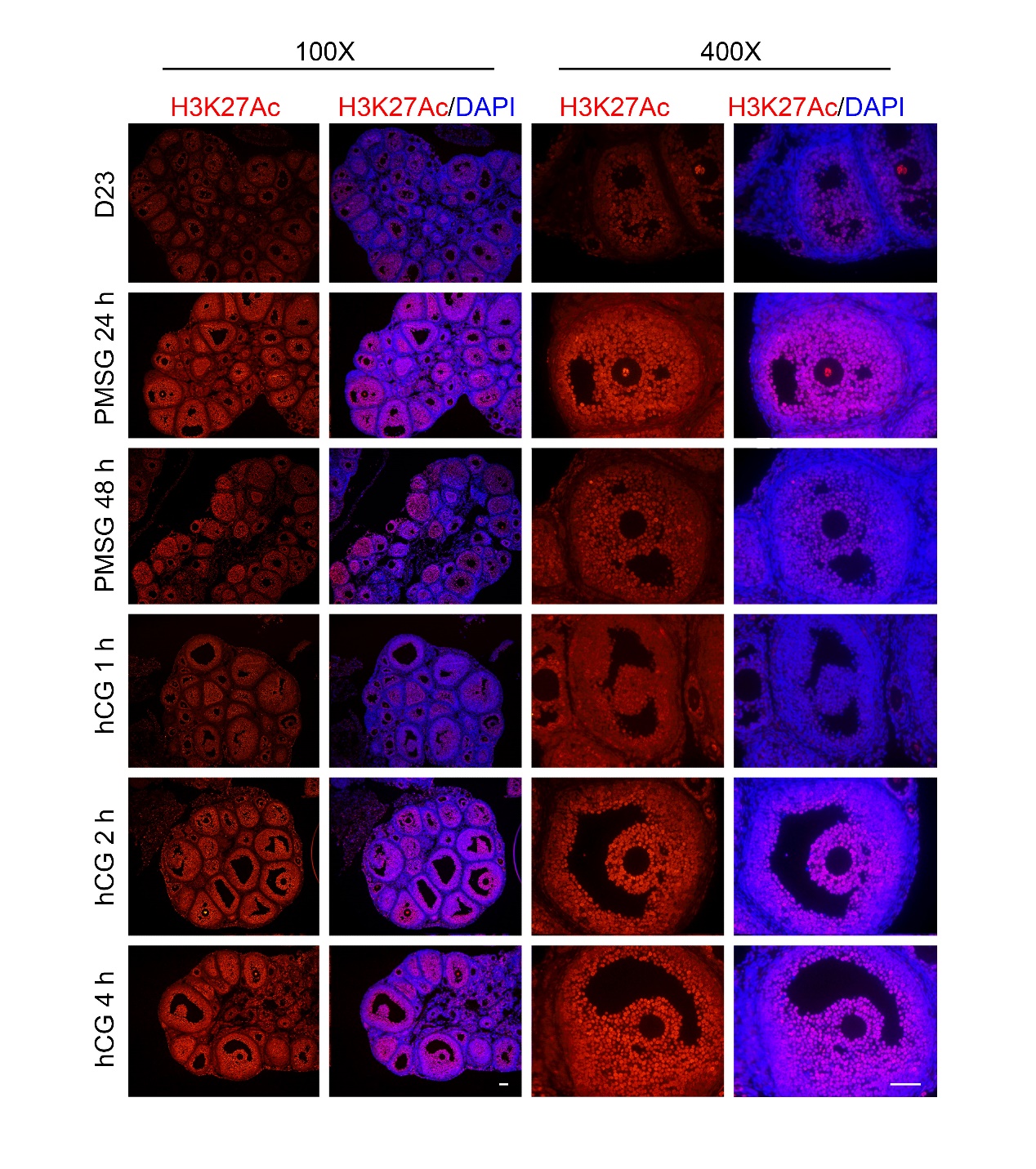


**Figure S1. The Dynamics of H3K27Ac Levels during Follicular Growth and Ovulation.** Mice were treated with pregnant mare serum gonadotrophin (PMSG) to stimulate follicle growth or human chorionic gonadotropin (hCG) to trigger ovulation. Ovaries were collected at the indicated timepoints for immunofluorescence. The representative images showing the H3K27Ac (red) levels with DAPI (blue) co-stained for visualization of nucleus (n=3). Scale bar=100um.


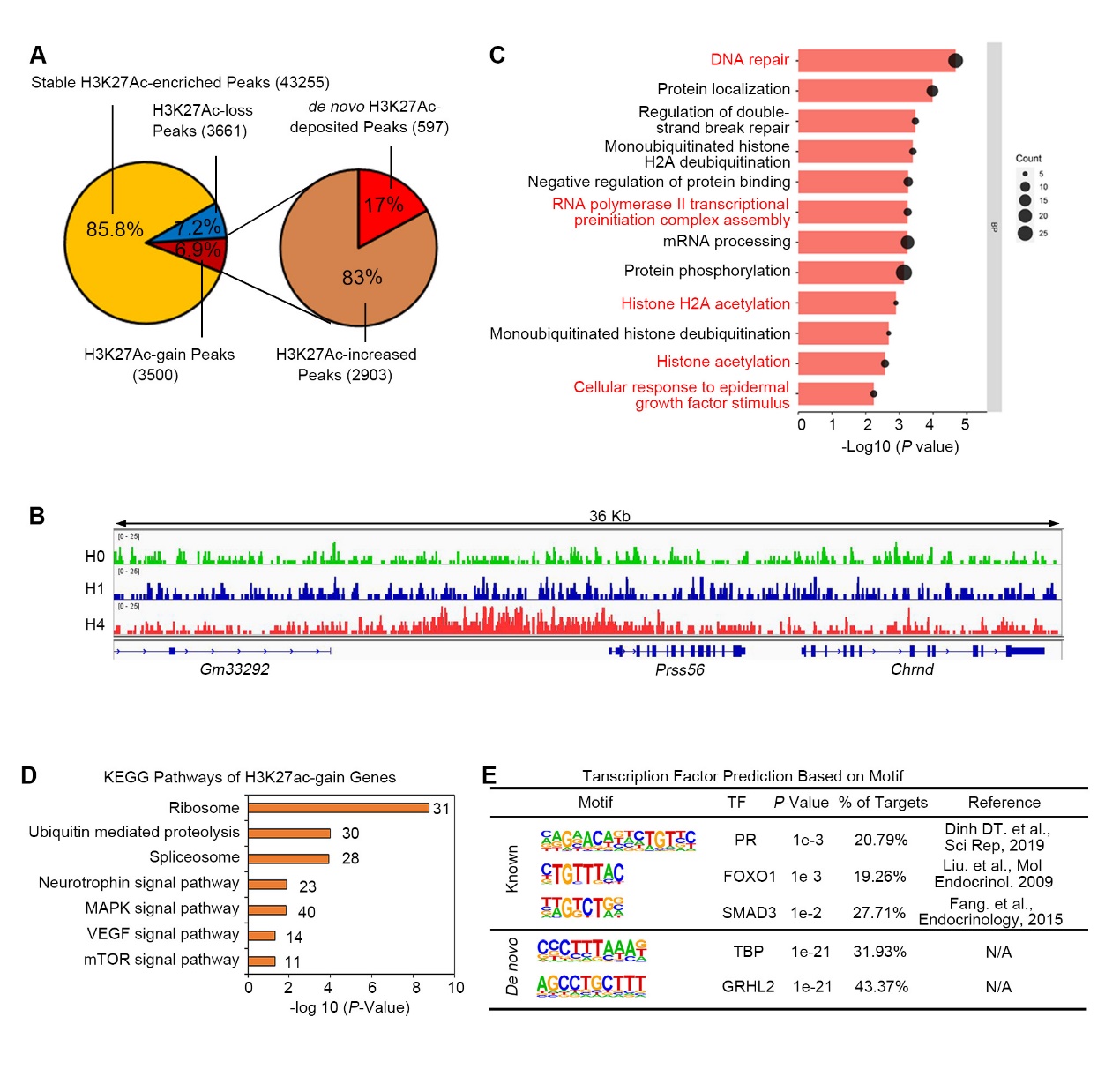


**Figure S2. The H3K27Ac ChIP-seq Analysis for H3K27Ac-gain Genes after Ovulation Signal Induction. (A)** Pie charts represent the ratio of stable H3K27Ac-enriched peaks, H3K27-loss peaks and H3K27-gain peakss (*de novo* H3K27Ac-deposited peaks and H3K27Ac-increased peaks). **(B)** Genome browser snapshot shows *Prss56* is one of *de novo* H3K27Ac-gain genes after hCG induction. **(C)** Gene Ontology (GO) analysis shows the biological process (BP) of *de novo* H3K27Ac-gain genes after hCG induction. **(D)** Bar charts representing the top enriched KEGG pathway of the ovulatory specific genes 4 h post hCG. **(E)** The HOMER known and *de novo* motif analysis for the H3K27Ac-gain peaks to predict transcriptional factors.


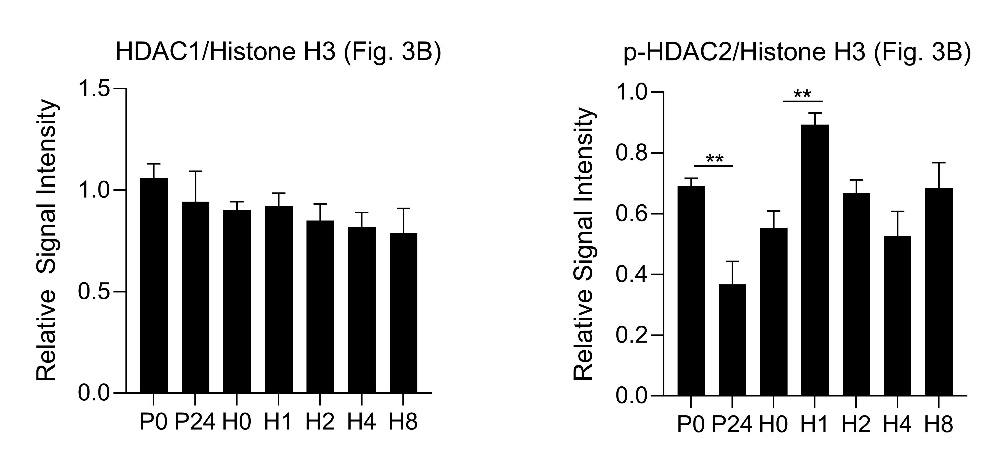


**Figure S3. Semiquantitative analysis of protein levels in Fig. 3.** The western blot band intensities of Fig. 3B were measured with ImageJ software. HDAC1 and p-HDAC2 protein levels were normalized to Histone H3 levels. Data were expressed as mean±SD. *P* value was determined by two-way ANOVA followed by Tukey’s post-test. ** *P*<0.01.


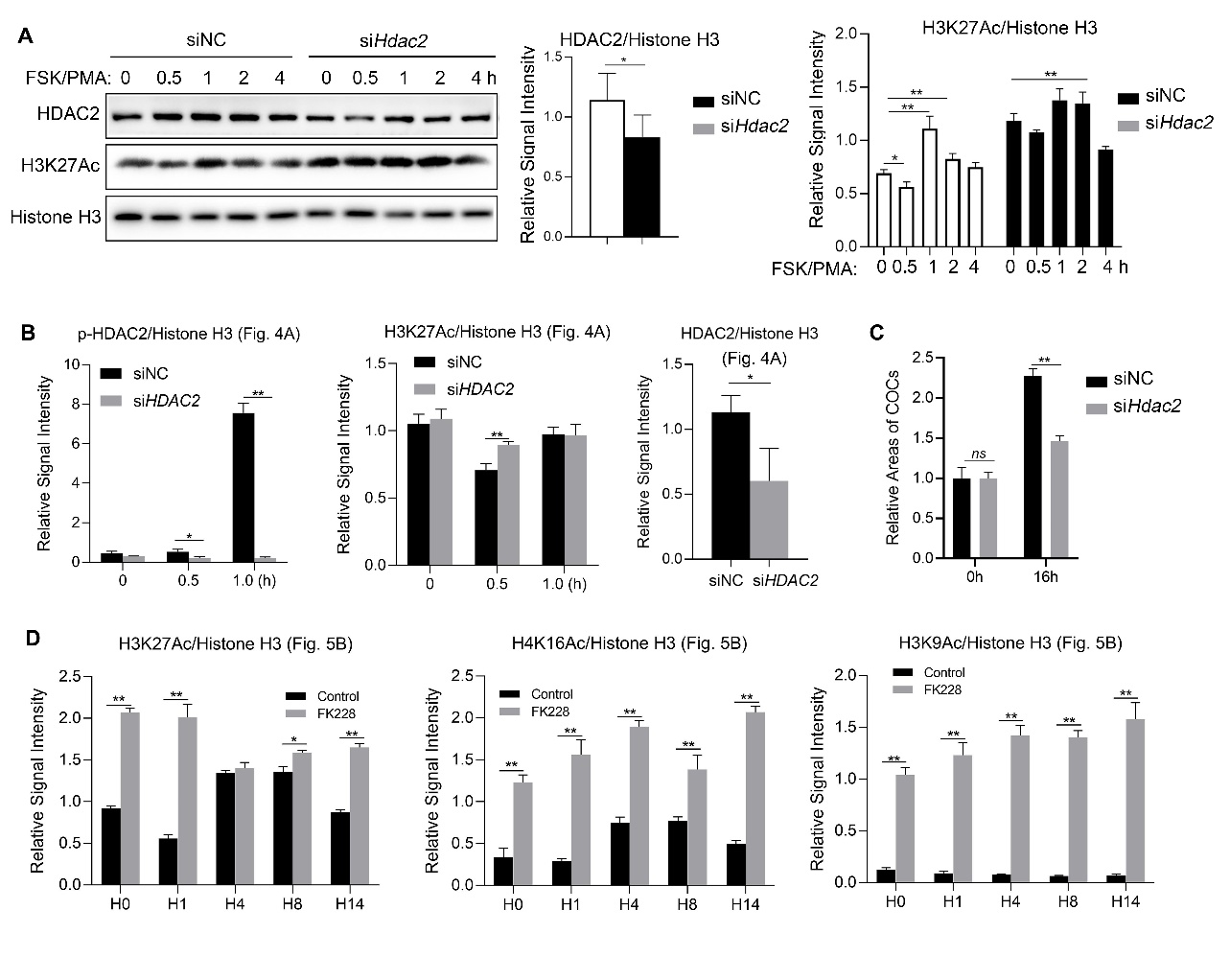


**Figure S4. Depletion or inhibition of HDAC2 Causes Increased H3K27Ac Levels and Affects COC expansion.** **(A)** Western blot analysis and quantification of HDAC2 and H3K27Ac levels in siNC and si*Hdac2* group. Primary granulosa cells were transfected of negative control (siNC) or *Hdac2* siRNAs (si*Hdac2*) for 36 h followed by treatment of forskolin (FSK, 10 μM) and phorbol 12-myristate 13-acetate (PMA, 20 nM) for indicated time length. All the protein levels were normalized to Histone H3 levels. The HDAC2 bands’ intensities were compared with siNC and si*Hdac2* groups in C. The bands’ intensities of H3K27Ac were compared among FSK/PMA non-treated group (0h) and FSK/PMA treated groups (0.5h, 1h, 2h and 4h). **(B)** Semiquantitative analysis of p-HDAC2 and H3K27Ac levels in Fig. 4A. The western blot band intensities of Fig. 4A were measured with ImageJ software. All protein levels were normalized to Histone H3 levels. The si*HDAC2* bands’ intensities were compared with siNC bands’ intensities. **(C)** Comparation of COCs expansion areas in Fig. 4B. The areas of COCs expansion in siNC and si*HDAC2* groups were measured with ImageJ software. **(D)** Quantitative analysis of H3K27Ac, H3K9Ac and H4K16Ac levels in Fig. 5B. All the protein levels were normalized to histone H3 levels. The FK228 bands’ intensities were compared with control bands’ intensities. All data in A-D were expressed as mean±SD. *P* value was determined by two-way ANOVA followed by Tukey’s post-test. ns. means no significance. * *P*<0.05, ** *P*<0.01.


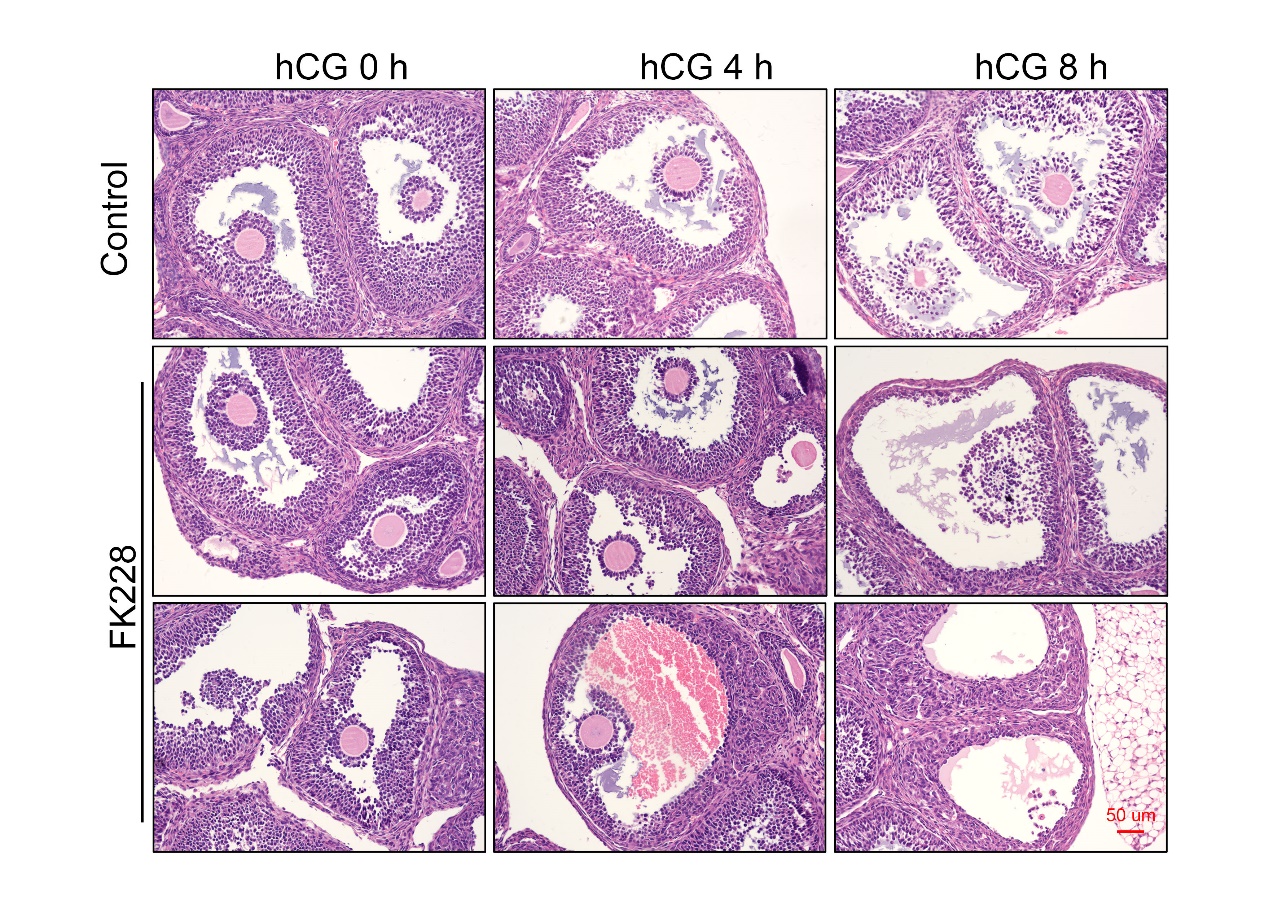


**Figure S5. Inhibition of HDAC1/2 Hinders Cumulus Expansion and Ovulation.** Hematoxylin and eosin (HE) staining results showing FK228, a HDAC1/2 inhibitor, blocks cumulus expansion and cause follicle atresia. Mice pretreated PMSG for 44 h were injected with DMSO or FK228, and 4 h later followed by hCG injection to induce ovulation (n=12). Ovaries were collected at 0 h, 4 h and 8 h post-hCG in mice of control and FK228 group. Scale bar, 50 um.


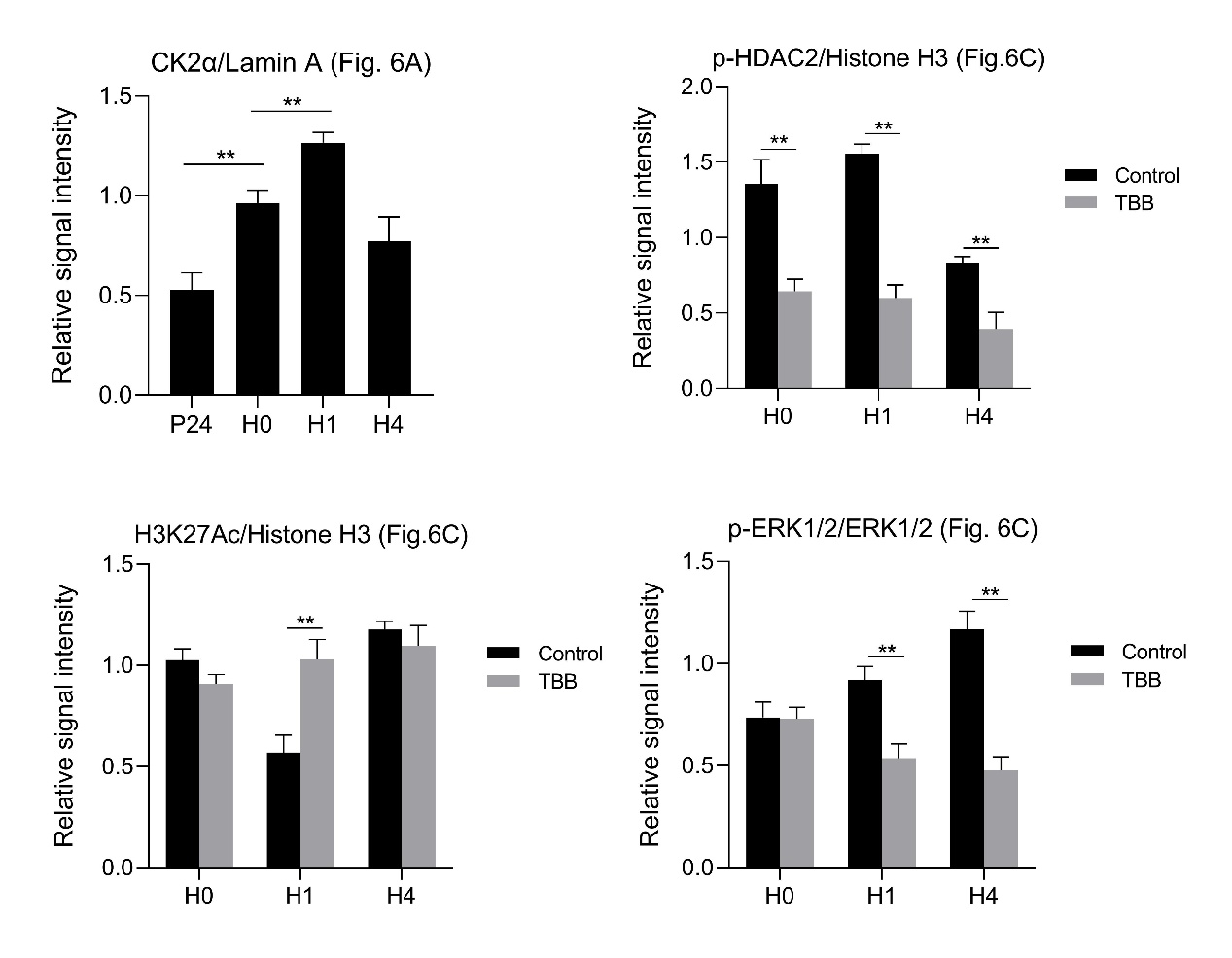


**Figure S6. Semiquantitative analysis of CK2α, p-HDAC2, H3K27Ac and p-ERK1/2 levels in Fig. 6A and C.** The western blot band intensities were measured with ImageJ software. CK2α, p-HDAC2 and H3K27Ac protein levels were normalized to Histone H3 levels and p-ERK1/2 levels were normalized to ERK1/2. The TBB bands’ intensities were compared with control bands’ intensities. Data were expressed as mean±SD. *P* value was determined by two-way ANOVA followed by Tukey’s post-test. ** *P*<0.01.
