# Supplementary material for: Ovulatory signal-triggered chromatin remodeling in ovarian granulosa cells by HDAC2 phosphorylation activation-mediated histone deacetylation
Source: Epigenetics Chromatin. 2023 Apr 19;16:11. doi: 10.1186/s13072-023-00485-8 (PMC10116676; doi:10.1186/s13072-023-00485-8)
Supplement: Supplementary file 2 — Additional file 2: Table S1. Oligonucleotides used for qPCR or siRNA. Table S2. Reagents or commercial kits used in this study. Table S3. The DEG list of RNA-seq data (H4 vs H0). [file 13072_2023_485_MOESM2_ESM.docx]

**Supplementary Tables**

**Table S1.** **Oligonucleotides used for qPCR or siRNA.**

| **Gene** | **Species** | **Application** | **RT-PCR primers (5’-3’)** |
| --- | --- | --- | --- |
| *Gapdh* | mouse | RT-qPCR | Forward: AGGTCGGTGTGAACGGATTTG  Reverse: GGGGTCGTTGATGGCAACA |
| *Areg* | mouse | RT-qPCR | Forward: GGTCTTAGGCTCAGGCCATTA  Reverse: CGCTTATGGTGGAAACCTCTC |
| *Ereg* | mouse | RT-qPCR | Forward: TTGGGTCTTGACGCTGCTTT  Reverse: TGCATGATGGGATCACGGTTG |
| *Sult1e1* | mouse | RT-qPCR | Forward: ATGGAGACTTCTATGCCTGAGT  Reverse: ACACAACTTCACTAATCCAGGTG |
| *Star* | mouse | RT-qPCR | Forward: CGGGTGGATGGGTCAAGTTC  Reverse: GCACTTCGTCCCCGTTCTC |
| *Cyp11a1* | mouse | RT-qPCR | Forward: AGGTCCTTCAATGAGATCCCTT  Reverse: TCCCTGTAAATGGGGCCATAC |
| *Fshr* | mouse | RT-qPCR | Forward: ATGTGTTCTCCAACCTACCCA  Reverse: GCTGGCAAGTGTTTAATGCCTG |
| *Lhcgr* | mouse | RT-qPCR | Forward: CTCGCCCGACTATCTCTCAC  Reverse: ACGACCTCATTAAGTCCCCTG |
| *Hdac1* | mouse | RT-qPCR | Forward: TGAAGCCTCACCGAATCCG  Reverse: GGGCGAATAGAACGCAGGA |
| *Hdac2* | mouse | RT-qPCR | Forward: GCTTGCCATCCTCGAATTACT  Reverse: GTCATCACGCGATCTGTTGTAT |
| *Hdac3* | mouse | RT-qPCR | Forward: CATCGCCTGGCATTGACTCAT  Reverse: AAGGCATTAAGGCTCTTGGTG |
| *Hdac4* | mouse | RT-qPCR | Forward: CTGCAAGTGGCCCCTACAG  Reverse: CTGCTCATGTTGACGCTGGA |
| *Hdac5* | mouse | RT-qPCR | Forward: TGCAGCACGTTTTGCTCCT  Reverse: GACAGCTCCCCAGTTTTGGT |
| *Hdac6* | mouse | RT-qPCR | Forward: TCCACCGGCCAAGATTCTTC  Reverse: GCCTTTCTTCTTTACCTCCGCT |
| *Hdac7* | mouse | RT-qPCR | Forward: TTCCTGGCAGGCTTACACC  Reverse: ATGGACTGTTCTCTCAAGGGC |
| *Hdac8* | mouse | RT-qPCR | Forward: ACTATTGCCGGAGATCCAATGT  Reverse: CCTCCTAAAATCAGAGTTGCCAG |
| *Hdac9* | mouse | RT-qPCR | Forward: CAGAAGCAGCACGAGAATTTGA  Reverse: CTCTCTGCGATGCCTCTCTAC |
| *Hdac10* | mouse | RT-qPCR | Forward: ACAGCCACTCGACTGCTCT  Reverse: GATGCCTCACAAGCTGACAAA |
| *Ereg* | mouse | ChIP-qPCR | Forward: CTTTGTCTAGGTAAGCCCTCGA  Reverse: CAACGTCAACCGACCAAGTC |
| *Star* | mouse | ChIP-qPCR | Forward: TTGGTTCTCAGCACCCATGT  Reverse: GAAAGAAAGAAAGGAGAGAACGC |
| *Sult1e1* | mouse | ChIP-qPCR | Forward: ACACTATTAGCCTTATGGAAACTGG  Reverse: GACGACATTTTCTAACCCTTAACAC |
| *Crem* | mouse | ChIP-qPCR | Forward: ATGGAAACAGTTGAATCACAGC  Reverse: ACTTTCATTGTACCTGCCTATGG |
| *Fshr* | mouse | ChIP-qPCR | Forward: CTATTGGCTGGAATGCATGAC  Reverse: TTTACAAGCCAAGGGCCTC |
| *Lhcgr* | mouse | ChIP-qPCR | Forward: GTTTCAGAGCATGTGTGTGTTGA  Reverse: CATCACTAAAACCCTTCTGACATAA |
| *Cyp11a1* | mouse | ChIP-qPCR | Forward: TACAGATGGTTGTGAGCCACC  Reverse: CCACAAGGTATGGGTTTCACA |
| *si-nc* | NA | siRNAs | UUCUCCGAACGUGUCACGUTT |
| si-*Hdac2* | mouse | siRNAs | GGAAUGUUGCUGAUCAUAA |
| si-*Hdac2* | mouse | siRNAs | GAUCAAUAAGACCAGAUAA |

**Table S2. Reagents or commercial kits used in this study.**

| Reagents or Assay Kits | Source | Identifier |
| --- | --- | --- |
| Antibodies | | |
| Normal Rabbit IgG | CST | Cat#2729 |
| Rabbit anti-pPoll II (S2) | Abcam | Cat#ab5095 |
| Rabbit anti-H3K27Ac | Abcam | Cat#ab177178 |
| Rabbit anti-H3K9Ac | Abcam | Cat#ab177177 |
| Rabbit anti-H4K16Ac | Abcam | Cat#ab109463 |
| Rabbit anti-Histone H3 | CST | Cat#4620 |
| Rabbit anti-HDAC1 | Proteintech | Cat#10197-1-AP |
| Rabbit anti-HDAC2 | Abcam | Cat#ab124974 |
| Rabbit anti-pHDAC2(S394) | Abcam | Cat#ab75602 |
| Rabbit anti-pERK1/2 | CST | Cat#4370 |
| Rabbit anti-ERK1/2 | CST | Cat#9102 |
| Rabbit anti-CK2α | Abcam | Cat#2656s |
| Rabbit anti-Lamin A | Abcam | Cat#133256 |
| Rabbit anti-GAPDH | Proteintech | Cat# 10494-1-AP |
| Chemicals | | |
| PMSG | San Sheng Bio. Tech |  |
| hCG | San Sheng Bio. Tech |  |
| Forskolin (cAMP inducer) | MCE | Cat#HY-15371 |
| PMA (PKC activator) | MCE | Cat#HY-18739 |
| FK228 (HDAC1/2 inhibitor) | MCE | Cat#HY-15149 |
| TBB (CK2α inhibitor) | MCE | Cat#HY-14394 |
| Critical Commercial Assays | | |
| HDAC2 activity: HDAC-Glo^TM^ I/II Assays | Promega | Cat#G6420 |
| NE-PER^®^ Nuclear and Cytoplasmic Extraction Reagents | Thermo Fisher | Cat#78833 |
| SimpleChIP^®^ Plus Enzymatic Chromatin IP Kit | CST | Cat#9005 |
| Software and Algorithms | | |
| Deeptools2 | Ramirez et al., 2016 | https://deeptools.readthedocs.io/en/develop/ |
| MACS2.0 | Zhang et al., 2008 | https://github.com/taoliu/MACS/ |
| Bedtools | Quinlan, 2014 | https://bedtools.readthedocs.io/en/latest/ |
| HOMER v4.7 | Heinz et al., 2010 | http://homer.ucsd.edu/homer/ |
| Integrative Genomics Viewer | Robinson et al., 2011 | http://www.igv.org/ |
| GraphPad Prism | N/A | https://www.graphpad.com/ |
| Image J | N/A | https://imagej.en.softonic.com/ |

**Table S3. The upregulated DEG list of RNA-seq data (H4 vs H0).**

| **Ensembl_gene** | **Gene symbol** | **baseMean** | **log2FoldChange** | **p-value** |
| --- | --- | --- | --- | --- |
| ENSMUSG00000034936 | *Arl4d* | 1021.474 | 11.03582 | 2.28E-12 |
| ENSMUSG00000029819 | *Npy* | 139.0123 | 8.066389 | 3.06E-12 |
| ENSMUSG00000057722 | *Lepr* | 426.1345 | 8.461465 | 9.64E-12 |
| ENSMUSG00000027832 | *Ptx3* | 509.5072 | 9.872189 | 1.30E-11 |
| ENSMUSG00000031937 | *Vstm5* | 93.47529 | 7.380391 | 4.57E-11 |
| ENSMUSG00000031574 | *Star* | 29160.36 | 7.808624 | 4.59E-11 |
| ENSMUSG00000082361 | *Btc* | 1139.988 | 11.22901 | 8.20E-11 |
| ENSMUSG00000067017 | *Gm3608* | 753.5293 | 11.16263 | 9.28E-11 |
| ENSMUSG00000024256 | *Adcyap1* | 97.35778 | 8.246148 | 1.12E-10 |
| ENSMUSG00000001300 | *Efnb2* | 956.6665 | 9.844836 | 2.11E-10 |
| ENSMUSG00000028307 | *Aldob* | 157.4366 | 8.678355 | 2.15E-10 |
| ENSMUSG00000090639 | *Gm20425* | 174.3789 | 8.718329 | 3.04E-10 |
| ENSMUSG00000031932 | *Gpr83* | 269.3507 | 9.060844 | 4.51E-10 |
| ENSMUSG00000029377 | *Ereg* | 2812.504 | 10.54042 | 5.17E-10 |
| ENSMUSG00000027350 | *Chgb* | 2155.334 | 10.10047 | 8.42E-10 |
| ENSMUSG00000026247 | *Ecel1* | 125.8871 | 8.94185 | 9.44E-10 |
| ENSMUSG00000030208 | *Emp1* | 463.8192 | 8.935591 | 1.00E-09 |
| ENSMUSG00000024912 | *Fosl1* | 551.3549 | 10.6029 | 1.02E-09 |
| ENSMUSG00000022357 | *Klhl38* | 697.6412 | 9.44074 | 1.22E-09 |
| ENSMUSG00000048807 | *Slc35e4* | 787.4929 | 11.27709 | 1.60E-09 |
| ENSMUSG00000049001 | *Ndnf* | 171.3516 | 8.726064 | 2.33E-09 |
| ENSMUSG00000042622 | *Maff* | 1160.153 | 10.52211 | 2.66E-09 |
| ENSMUSG00000032487 | *Ptgs2* | 2247.166 | 10.45391 | 2.90E-09 |
| ENSMUSG00000031870 | *Pgr* | 4072.436 | 8.842309 | 3.06E-09 |
| ENSMUSG00000032554 | *Trf* | 11328.61 | 6.580893 | 3.55E-09 |
| ENSMUSG00000051499 | *Zfp786* | 98.3509 | 7.674311 | 5.00E-09 |
| ENSMUSG00000020262 | *Adarb1* | 1181.501 | 7.891261 | 5.60E-09 |
| ENSMUSG00000028211 | *Trp53inp1* | 2659.866 | 8.744913 | 7.27E-09 |
| ENSMUSG00000030303 | *Far2* | 148.3694 | 8.324379 | 7.28E-09 |
| ENSMUSG00000022533 | *Atp13a3* | 8229.956 | 6.694258 | 8.25E-09 |
| ENSMUSG00000003484 | *Cyp4f18* | 87.15668 | 8.05509 | 9.81E-09 |
| ENSMUSG00000035566 | *Pcdh17* | 1618.756 | 9.39521 | 1.22E-08 |
| ENSMUSG00000028128 | *F3* | 1275.083 | 8.506984 | 1.29E-08 |
| ENSMUSG00000046722 | *Cdc42se1* | 1814.981 | 7.182579 | 1.39E-08 |
| ENSMUSG00000045573 | *Penk* | 608.624 | 8.720484 | 1.42E-08 |
| ENSMUSG00000027273 | *Snap25* | 566.1129 | 10.27395 | 1.53E-08 |
| ENSMUSG00000021091 | *Serpina3n* | 176.5777 | 7.820848 | 1.65E-08 |
| ENSMUSG00000113667 | *AC122840* | 333.9044 | 8.493604 | 1.74E-08 |
| ENSMUSG00000013846 | *St3gal1* | 8029.538 | 8.562751 | 1.74E-08 |
| ENSMUSG00000078719 | *Msmp* | 221.981 | 9.018159 | 2.44E-08 |
| ENSMUSG00000070687 | *Htr1d* | 98.15534 | 8.205812 | 2.82E-08 |
| ENSMUSG00000057278 | *Snrpg* | 976.1703 | 8.073935 | 2.85E-08 |
| ENSMUSG00000115499 | *CT025525* | 3286.201 | 9.436736 | 2.87E-08 |
| ENSMUSG00000017764 | *Zswim1* | 1102.421 | 9.388381 | 3.02E-08 |
| ENSMUSG00000029771 | *Irf5* | 49.15789 | 6.741244 | 3.42E-08 |
| ENSMUSG00000054079 | *Utp18* | 2960.531 | 9.526751 | 3.65E-08 |
| ENSMUSG00000024889 | *Rce1* | 939.5123 | 8.089018 | 3.84E-08 |
| ENSMUSG00000022383 | *Ppara* | 136.6904 | 8.23981 | 4.41E-08 |
| ENSMUSG00000031971 | *Ccsap* | 495.3818 | 8.13008 | 4.77E-08 |
| ENSMUSG00000025089 | *Gfra1* | 847.3876 | 9.919131 | 5.32E-08 |
| ENSMUSG00000062393 | *Dgkk* | 308.8948 | 8.833968 | 6.21E-08 |
| ENSMUSG00000109645 | *Gm45246* | 76.87379 | 7.283992 | 7.51E-08 |
| ENSMUSG00000046711 | *Hmga1* | 5350.509 | 6.753887 | 7.53E-08 |
| ENSMUSG00000024897 | *Apba1* | 67.72708 | 7.684065 | 8.28E-08 |
| ENSMUSG00000038370 | *Pcp4l1* | 247.3213 | 8.286289 | 8.91E-08 |
| ENSMUSG00000027577 | *Chrna4* | 100.8234 | 8.862081 | 1.06E-07 |
| ENSMUSG00000047250 | *Ptgs1* | 46.45644 | 7.354569 | 1.12E-07 |
| ENSMUSG00000025582 | *Nptx1* | 116.3484 | 6.89659 | 1.48E-07 |
| ENSMUSG00000055763 | *Rybp* | 122.1134 | 8.721076 | 1.57E-07 |
| ENSMUSG00000039942 | *Ptger4* | 1351.311 | 9.659944 | 1.69E-07 |
| ENSMUSG00000045827 | *Serpinb9* | 425.6837 | 8.43699 | 1.91E-07 |
| ENSMUSG00000000627 | *Sema4f* | 155.7893 | 8.231552 | 2.00E-07 |
| ENSMUSG00000050613 | *Olfr125* | 65.71399 | 7.234219 | 2.54E-07 |
| ENSMUSG00000058239 | *Usf2* | 2771.339 | 6.551809 | 2.55E-07 |
| ENSMUSG00000022952 | *Runx1* | 18848.71 | 5.31759 | 2.75E-07 |
| ENSMUSG00000056752 | *Dnah9* | 80.50508 | 8.354733 | 2.75E-07 |
| ENSMUSG00000029161 | *Cgref1* | 75.16734 | 7.475354 | 2.76E-07 |
| ENSMUSG00000029378 | *Areg* | 164.569 | 7.745179 | 2.83E-07 |
| ENSMUSG00000030022 | *Adamts9* | 9931.741 | 6.260582 | 2.88E-07 |
| ENSMUSG00000020561 | *Twistnb* | 181.6146 | 7.317201 | 2.90E-07 |
| ENSMUSG00000090035 | *Galnt4* | 400.7111 | 8.736905 | 3.28E-07 |
| ENSMUSG00000109916 | *Gm45551* | 1390.408 | 7.231015 | 3.38E-07 |
| ENSMUSG00000028581 | *Laptm5* | 1001.668 | 8.614559 | 3.60E-07 |
| ENSMUSG00000025060 | *Slk* | 2262.827 | 6.493369 | 3.71E-07 |
| ENSMUSG00000031370 | *Zrsr2* | 15147.26 | 7.569044 | 3.73E-07 |
| ENSMUSG00000020176 | *Grb10* | 1020.969 | 8.26668 | 3.76E-07 |
| ENSMUSG00000030830 | *Itgal* | 33.45518 | 6.588466 | 4.02E-07 |
| ENSMUSG00000046223 | *Plaur* | 292.6402 | 8.408608 | 4.03E-07 |
| ENSMUSG00000026798 | *Coq4* | 562.0109 | 8.249394 | 4.18E-07 |
| ENSMUSG00000074305 | *Peak1* | 3528.435 | 4.939761 | 4.22E-07 |
| ENSMUSG00000046203 | *Sprr2g* | 49.13421 | 7.223375 | 4.23E-07 |
| ENSMUSG00000037428 | *Vgf* | 39.99678 | 6.755465 | 4.80E-07 |
| ENSMUSG00000038518 | *Jarid2* | 6578.599 | 4.315173 | 5.29E-07 |
| ENSMUSG00000048776 | *Pthlh* | 321.6035 | 8.824157 | 5.42E-07 |
| ENSMUSG00000026204 | *Ptprn* | 2109.712 | 8.726669 | 5.46E-07 |
| ENSMUSG00000027340 | *Slc23a2* | 31899.04 | 5.258226 | 6.02E-07 |
| ENSMUSG00000030787 | *Lyve1* | 26.44421 | 6.224513 | 6.30E-07 |
| ENSMUSG00000020183 | *Cpm* | 54.56376 | 7.236773 | 6.44E-07 |
| ENSMUSG00000042821 | *Snai1* | 58.21964 | 6.42631 | 6.44E-07 |
| ENSMUSG00000052997 | *Uba2* | 3140.433 | 7.265533 | 6.49E-07 |
| ENSMUSG00000020592 | *Sdc1* | 17926.71 | 4.604541 | 7.15E-07 |
| ENSMUSG00000045382 | *Cxcr4* | 1726.813 | 8.62 | 7.16E-07 |
| ENSMUSG00000053475 | *Tnfaip6* | 451.1812 | 8.166184 | 7.53E-07 |
| ENSMUSG00000031217 | *Efnb1* | 3593.751 | 6.487522 | 8.01E-07 |
| ENSMUSG00000029992 | *Gfpt1* | 4425.799 | 6.916927 | 8.12E-07 |
| ENSMUSG00000011179 | *Odc1* | 28144.73 | 5.257882 | 8.63E-07 |
| ENSMUSG00000033847 | *Pla2g4c* | 508.9827 | 9.107957 | 8.77E-07 |
| ENSMUSG00000029272 | *Sult1e1* | 1222.818 | 8.915432 | 9.08E-07 |
| ENSMUSG00000027996 | *Sfrp2* | 2034.012 | 7.057561 | 9.61E-07 |
| ENSMUSG00000033214 | *Slitrk5* | 56.43672 | 7.234736 | 1.02E-06 |
| ENSMUSG00000018849 | *Wwc1* | 100.622 | 7.303614 | 1.04E-06 |
| ENSMUSG00000078117 | *Gm16485* | 162.3432 | 8.026326 | 1.11E-06 |
| ENSMUSG00000019890 | *Nts* | 160.7601 | 7.156837 | 1.14E-06 |
| ENSMUSG00000026837 | *Col5a1* | 6343.574 | 6.271661 | 1.32E-06 |
| ENSMUSG00000023236 | *Scg5* | 35.49165 | 6.183201 | 1.34E-06 |
| ENSMUSG00000020248 | *Nfyb* | 1220.73 | 7.891953 | 1.46E-06 |
| ENSMUSG00000029304 | *Spp1* | 7412.579 | 5.86806 | 1.50E-06 |
| ENSMUSG00000043091 | *Tuba1c* | 6310.481 | 6.556692 | 1.51E-06 |
| ENSMUSG00000022425 | *Enpp2* | 39.26527 | 6.165353 | 1.58E-06 |
| ENSMUSG00000024395 | *Lims2* | 49.90323 | 6.81113 | 1.59E-06 |
| ENSMUSG00000107383 | *Gm4366* | 559.6927 | 8.374584 | 1.69E-06 |
| ENSMUSG00000043635 | *Adamts3* | 31.75134 | 6.740663 | 1.95E-06 |
| ENSMUSG00000035020 | *Epgn* | 33.44933 | 5.915512 | 1.95E-06 |
| ENSMUSG00000057933 | *Gsta2* | 59.44421 | 6.085204 | 1.99E-06 |
| ENSMUSG00000026341 | *Actr3* | 4324.864 | 6.393964 | 2.01E-06 |
| ENSMUSG00000028341 | *Nr4a3* | 509.5266 | 6.822727 | 2.12E-06 |
| ENSMUSG00000045538 | *Ddx28* | 456.1583 | 8.065316 | 2.29E-06 |
| ENSMUSG00000024427 | *Spry4* | 436.4727 | 8.281103 | 2.61E-06 |
| ENSMUSG00000051335 | *Gfod1* | 974.9567 | 7.943686 | 2.64E-06 |
| ENSMUSG00000102367 | *Kiss1* | 21.7573 | 5.95422 | 2.67E-06 |
| ENSMUSG00000034040 | *Galnt17* | 378.6159 | 8.587142 | 2.68E-06 |
| ENSMUSG00000031843 | *Mphosph6* | 636.7351 | 8.717744 | 3.03E-06 |
| ENSMUSG00000033730 | *Egr3* | 630.8738 | 8.066994 | 3.33E-06 |
| ENSMUSG00000029236 | *Nmu* | 18.05508 | 5.594819 | 3.35E-06 |
| ENSMUSG00000027230 | *Creb3l1* | 2590.45 | 7.581149 | 3.40E-06 |
| ENSMUSG00000109925 | *Gm45315* | 2831.768 | 8.022471 | 3.56E-06 |
| ENSMUSG00000027947 | *Il6ra* | 220.1818 | 7.524493 | 3.57E-06 |
| ENSMUSG00000094638 | *Gm21972* | 150.3685 | 7.471396 | 3.64E-06 |
| ENSMUSG00000031963 | *Bmper* | 1368.481 | 7.468308 | 3.75E-06 |
| ENSMUSG00000072582 | *Ptrh2* | 35.04526 | 6.08225 | 3.82E-06 |
| ENSMUSG00000022602 | *Arc* | 137.3696 | 6.211267 | 3.96E-06 |
| ENSMUSG00000028495 | *Rps6* | 13475.16 | 6.459121 | 4.04E-06 |
| ENSMUSG00000041153 | *Osgin2* | 3433.122 | 6.656931 | 4.10E-06 |
| ENSMUSG00000039956 | *Mrap* | 2858.123 | 5.996965 | 4.18E-06 |
| ENSMUSG00000100309 | *Gm6644* | 48.01104 | 6.98308 | 4.20E-06 |
| ENSMUSG00000044217 | *Aqp5* | 170.6181 | 8.348574 | 4.21E-06 |
| ENSMUSG00000032549 | *Rab6b* | 109.6348 | 6.531762 | 4.40E-06 |
| ENSMUSG00000094504 | *Gm5294* | 119.422 | 7.05013 | 4.92E-06 |
| ENSMUSG00000019851 | *Perp* | 527.5121 | 7.105238 | 5.04E-06 |
| ENSMUSG00000108793 | *Gm45213* | 50.08201 | 6.822698 | 5.08E-06 |
| ENSMUSG00000042359 | *Osbpl6* | 5699.945 | 6.332103 | 5.13E-06 |
| ENSMUSG00000086965 | *Rtl10* | 219.5927 | 7.736767 | 5.13E-06 |
| ENSMUSG00000035131 | *Brinp3* | 17.49765 | 5.595988 | 5.36E-06 |
| ENSMUSG00000037979 | *Ccdc92* | 120.5503 | 8.030275 | 5.56E-06 |
| ENSMUSG00000089996 | *Tmsb15b2* | 603.9517 | 7.303657 | 5.72E-06 |
| ENSMUSG00000038028 | *Tigar* | 422.8601 | 7.469236 | 5.80E-06 |
| ENSMUSG00000030411 | *Nova2* | 74.05951 | 6.883466 | 5.94E-06 |
| ENSMUSG00000056220 | *Pla2g4a* | 971.1432 | 6.279436 | 6.00E-06 |
| ENSMUSG00000115232 | *AC174678* | 577.8261 | 8.977234 | 6.03E-06 |
| ENSMUSG00000032849 | *Abcc4* | 3033.405 | 7.593407 | 6.24E-06 |
| ENSMUSG00000029131 | *Dnajb6* | 9734.177 | 6.21666 | 6.45E-06 |
| ENSMUSG00000043948 | *Olfr691* | 30.16671 | 6.171692 | 6.64E-06 |
| ENSMUSG00000096188 | *Cmtm4* | 3663.47 | 6.010545 | 6.68E-06 |
| ENSMUSG00000029918 | *Mrps33* | 2107.901 | 8.336445 | 7.03E-06 |
| ENSMUSG00000028197 | *Col24a1* | 635.6717 | 7.427303 | 7.07E-06 |
| ENSMUSG00000030351 | *Tspan11* | 201.1403 | 8.17649 | 7.75E-06 |
| ENSMUSG00000001305 | *Rrp15* | 3876.629 | 7.181606 | 7.78E-06 |
| ENSMUSG00000028645 | *Slc2a1* | 6228.313 | 4.87862 | 7.99E-06 |
| ENSMUSG00000006014 | *Prg4* | 634.1377 | 7.588679 | 8.40E-06 |
| ENSMUSG00000032333 | *Stoml1* | 2187.231 | 7.233848 | 8.69E-06 |
| ENSMUSG00000031107 | *Rbmx2* | 757.4179 | 7.662132 | 8.69E-06 |
| ENSMUSG00000030064 | *Frmd4b* | 518.1684 | 7.309222 | 8.73E-06 |
| ENSMUSG00000022180 | *Slc7a8* | 34140.85 | 4.068255 | 8.88E-06 |
| ENSMUSG00000059866 | *Tnip2* | 2609.507 | 7.381485 | 8.96E-06 |
| ENSMUSG00000021010 | *Npas3* | 79.05626 | 6.548544 | 9.07E-06 |
| ENSMUSG00000032548 | *Slco2a1* | 3188.061 | 6.4449 | 9.29E-06 |
| ENSMUSG00000038331 | *Satb2* | 960.4999 | 7.394743 | 9.45E-06 |
| ENSMUSG00000025608 | *Podxl* | 1036.693 | 6.690299 | 1.01E-05 |
| ENSMUSG00000023905 | *Tnfrsf12a* | 474.4017 | 7.222387 | 1.07E-05 |
| ENSMUSG00000045624 | *Esf1* | 27939.16 | 7.587259 | 1.09E-05 |
| ENSMUSG00000026317 | *Cln8* | 3211.92 | 5.218284 | 1.09E-05 |
| ENSMUSG00000114245 | *AC130711* | 627.6417 | 8.349425 | 1.11E-05 |
| ENSMUSG00000018238 | *Gdf9* | 4479.012 | 7.513587 | 1.12E-05 |
| ENSMUSG00000035355 | *Kcnh4* | 17.42979 | 5.383861 | 1.17E-05 |
| ENSMUSG00000036721 | *Zscan12* | 312.1394 | 6.906751 | 1.19E-05 |
| ENSMUSG00000022391 | *Rangap1* | 39533.88 | 6.259753 | 1.19E-05 |
| ENSMUSG00000091243 | *Vgll3* | 127.8607 | 7.14689 | 1.24E-05 |
| ENSMUSG00000005087 | *Cd44* | 2317.819 | 8.337039 | 1.27E-05 |
| ENSMUSG00000051339 | *2900026A02Rik* | 755.0964 | 5.821522 | 1.29E-05 |
| ENSMUSG00000066245 | *Gm10156* | 15.6071 | 5.407069 | 1.31E-05 |
| ENSMUSG00000037936 | *Scarb1* | 26846.76 | 3.666856 | 1.33E-05 |
| ENSMUSG00000049100 | *Pcdh10* | 4482.676 | 6.785531 | 1.34E-05 |
| ENSMUSG00000025364 | *Pa2g4* | 10895.94 | 5.939699 | 1.47E-05 |
| ENSMUSG00000050973 | *Gdpgp1* | 190.3601 | 7.211896 | 1.50E-05 |
| ENSMUSG00000002980 | *Bcam* | 2953.139 | 6.964329 | 1.51E-05 |
| ENSMUSG00000020911 | *Krt19* | 95.17234 | 7.889536 | 1.51E-05 |
| ENSMUSG00000030691 | *Fchsd2* | 4876.654 | 6.142377 | 1.53E-05 |
| ENSMUSG00000048997 | *Atxn7l2* | 614.0735 | 6.293458 | 1.56E-05 |
| ENSMUSG00000022120 | *Rnf219* | 719.4367 | 6.323118 | 1.59E-05 |
| ENSMUSG00000030041 | *M1ap* | 37.56759 | 6.46103 | 1.63E-05 |
| ENSMUSG00000041633 | *Kctd12b* | 1276.826 | 7.000403 | 1.67E-05 |
| ENSMUSG00000024953 | *Prdx5* | 3110.568 | 7.060256 | 1.71E-05 |
| ENSMUSG00000083282 | *Ctsf* | 1513.694 | 5.83519 | 1.72E-05 |
| ENSMUSG00000026344 | *Lypd1* | 73.56343 | 6.097849 | 1.75E-05 |
| ENSMUSG00000052131 | *Akr1b7* | 82.8329 | 7.228154 | 1.83E-05 |
| ENSMUSG00000034591 | *Slc41a2* | 52.02529 | 4.955693 | 1.88E-05 |
| ENSMUSG00000058743 | *Kcnj14* | 19.70864 | 5.63499 | 1.96E-05 |
| ENSMUSG00000031762 | *Mt2* | 1365.229 | 5.33552 | 1.99E-05 |
| ENSMUSG00000052363 | *Zdhhc19* | 20.6961 | 5.66062 | 2.01E-05 |
| ENSMUSG00000031444 | *F10* | 204.9313 | 7.376432 | 2.07E-05 |
| ENSMUSG00000020804 | *Aanat* | 42.97059 | 6.495504 | 2.08E-05 |
| ENSMUSG00000010803 | *Gabra1* | 54.05181 | 6.098994 | 2.14E-05 |
| ENSMUSG00000047604 | *Frat2* | 164.8055 | 7.145312 | 2.18E-05 |
| ENSMUSG00000044664 | *Prss42* | 169.9964 | 7.740385 | 2.19E-05 |
| ENSMUSG00000024383 | *Map3k2* | 1161.793 | 5.990119 | 2.21E-05 |
| ENSMUSG00000015533 | *Itga2* | 10106.31 | 5.0217 | 2.23E-05 |
| ENSMUSG00000022216 | *Psme1* | 5613.608 | 5.316879 | 2.25E-05 |
| ENSMUSG00000038305 | *Spats2l* | 60.73723 | 5.820429 | 2.25E-05 |
| ENSMUSG00000079428 | *Tceal7* | 17.09277 | 5.558905 | 2.26E-05 |
| ENSMUSG00000044499 | *Hs3st5* | 19.94835 | 5.177299 | 2.28E-05 |
| ENSMUSG00000039202 | *Abhd2* | 17580.57 | 4.100974 | 2.30E-05 |
| ENSMUSG00000019872 | *Smpdl3a* | 29.49651 | 5.646559 | 2.36E-05 |
| ENSMUSG00000033854 | *Kcnk10* | 22.46314 | 5.108049 | 2.37E-05 |
| ENSMUSG00000036087 | *Slain2* | 2432.584 | 6.10276 | 2.38E-05 |
| ENSMUSG00000058897 | *Col25a1* | 264.4489 | 6.66932 | 2.39E-05 |
| ENSMUSG00000004642 | *Slbp* | 897.5101 | 5.792965 | 2.47E-05 |
| ENSMUSG00000062991 | *Nrg1* | 87.20674 | 5.454847 | 2.49E-05 |
| ENSMUSG00000021025 | *Nfkbia* | 2171.007 | 5.913518 | 2.52E-05 |
| ENSMUSG00000029270 | *Fam69a* | 80.99754 | 6.967844 | 2.69E-05 |
| ENSMUSG00000031320 | *Rps4x* | 11238.22 | 6.115946 | 2.75E-05 |
| ENSMUSG00000006403 | *Adamts4* | 799.3276 | 7.788661 | 2.75E-05 |
| ENSMUSG00000070644 | *Etnk2* | 3195.992 | 6.491462 | 2.75E-05 |
| ENSMUSG00000056749 | *Nfil3* | 241.5234 | 6.448265 | 2.82E-05 |
| ENSMUSG00000071089 | *Trim75* | 183.7944 | 6.814669 | 2.83E-05 |
| ENSMUSG00000028656 | *Cap1* | 8855.503 | 5.884128 | 2.85E-05 |
| ENSMUSG00000027547 | *Sall4* | 105.1347 | 6.651715 | 2.85E-05 |
| ENSMUSG00000018848 | *Rars* | 1939.535 | 7.227086 | 2.87E-05 |
| ENSMUSG00000024646 | *Cyb5a* | 9342.084 | 5.281948 | 2.92E-05 |
| ENSMUSG00000039809 | *Gabbr2* | 24.09239 | 6.055079 | 2.94E-05 |
| ENSMUSG00000036639 | *Nudt1* | 139.578 | 6.192572 | 2.98E-05 |
| ENSMUSG00000032726 | *Bmp8a* | 11.45897 | 4.833272 | 3.02E-05 |
| ENSMUSG00000045392 | *Olfr1033* | 8782.107 | 4.802096 | 3.03E-05 |
| ENSMUSG00000109325 | *Krt8* | 38.1928 | 6.078509 | 3.08E-05 |
| ENSMUSG00000051557 | *Pusl1* | 1354.905 | 7.612082 | 3.13E-05 |
| ENSMUSG00000074971 | *Fibin* | 30.14461 | 6.207503 | 3.13E-05 |
| ENSMUSG00000031584 | *Gsr* | 1369.026 | 6.966151 | 3.18E-05 |
| ENSMUSG00000062380 | *Tubb3* | 28.27794 | 6.334876 | 3.20E-05 |
| ENSMUSG00000047126 | *Cltc* | 9629.985 | 3.921289 | 3.29E-05 |
| ENSMUSG00000081788 | *Gm5898* | 2364.681 | 9.56718 | 3.32E-05 |
| ENSMUSG00000063568 | *Jazf1* | 56.56498 | 7.07455 | 3.33E-05 |
| ENSMUSG00000018736 | *Ndel1* | 1947.781 | 5.645041 | 3.35E-05 |
| ENSMUSG00000008373 | *Prpf31* | 4210.469 | 6.995033 | 3.35E-05 |
| ENSMUSG00000035258 | *Abi3bp* | 545.4593 | 6.279918 | 3.45E-05 |
| ENSMUSG00000005615 | *Pcyt1a* | 8331.793 | 5.621469 | 3.47E-05 |
| ENSMUSG00000021319 | *Sfrp4* | 37884.74 | 5.237184 | 3.47E-05 |
| ENSMUSG00000032353 | *Tmed3* | 1026.409 | 6.632836 | 3.80E-05 |
| ENSMUSG00000020905 | *Usp43* | 166.6436 | 6.198322 | 3.84E-05 |
| ENSMUSG00000026078 | *Pdcl3* | 2641.739 | 7.606064 | 3.93E-05 |
| ENSMUSG00000081185 | *Gm4852* | 87.6134 | 7.515673 | 3.96E-05 |
| ENSMUSG00000003541 | *Ier3* | 1252.041 | 5.641301 | 4.18E-05 |
| ENSMUSG00000035783 | *Acta2* | 12566.61 | 5.671333 | 4.20E-05 |
| ENSMUSG00000021681 | *Aggf1* | 1775.611 | 6.607161 | 4.26E-05 |
| ENSMUSG00000026003 | *Acadl* | 404.9798 | 5.390325 | 4.32E-05 |
| ENSMUSG00000021395 | *Spin1* | 6568.439 | 3.99465 | 4.32E-05 |
| ENSMUSG00000024331 | *Dsc2* | 78.94143 | 6.055992 | 4.32E-05 |
| ENSMUSG00000040658 | *Dnph1* | 1674.852 | 7.677969 | 4.53E-05 |
| ENSMUSG00000021591 | *Glrx* | 75.18191 | 6.904741 | 4.94E-05 |
| ENSMUSG00000060121 | *Gemin2* | 69.32392 | 5.799474 | 5.08E-05 |
| ENSMUSG00000041536 | *Serpina3a* | 15.15841 | 4.464593 | 5.30E-05 |
| ENSMUSG00000036295 | *Lrrn3* | 66.01801 | 5.89573 | 5.33E-05 |
| ENSMUSG00000036167 | *Pphln1* | 809.6017 | 5.451956 | 5.39E-05 |
| ENSMUSG00000029146 | *Snx17* | 4661.696 | 5.554167 | 5.46E-05 |
| ENSMUSG00000059775 | *Rps26* | 773.8538 | 6.0149 | 5.53E-05 |
| ENSMUSG00000050359 | *Sprr1a* | 11.1794 | 4.411502 | 5.55E-05 |
| ENSMUSG00000024258 | *Polr2d* | 82.9381 | 6.167337 | 5.59E-05 |
| ENSMUSG00000026698 | *Pigc* | 288.1747 | 6.211286 | 5.60E-05 |
| ENSMUSG00000033107 | *Rnf125* | 174.3375 | 7.239899 | 5.70E-05 |
| ENSMUSG00000019558 | *Slc6a8* | 2938.039 | 4.601119 | 5.77E-05 |
| ENSMUSG00000020872 | *Tac4* | 32.6102 | 5.964383 | 5.82E-05 |
| ENSMUSG00000042279 | *H1foo* | 377.2958 | 6.139602 | 5.85E-05 |
| ENSMUSG00000078941 | *Ak6* | 352.6749 | 5.991589 | 5.96E-05 |
| ENSMUSG00000073294 | *AU022751* | 413.141 | 6.445686 | 6.02E-05 |
| ENSMUSG00000047260 | *Emc6* | 991.1841 | 7.1887 | 6.03E-05 |
| ENSMUSG00000063952 | *Brpf3* | 15653.72 | 5.111995 | 6.34E-05 |
| ENSMUSG00000008206 | *Cers4* | 1307.756 | 6.379543 | 6.59E-05 |
| ENSMUSG00000022024 | *Sugt1* | 1213.319 | 5.270588 | 6.72E-05 |
| ENSMUSG00000021061 | *Sptb* | 5807.843 | 5.557609 | 6.72E-05 |
| ENSMUSG00000029725 | *Ppp1r35* | 209.6907 | 5.813453 | 6.90E-05 |
| ENSMUSG00000035394 | *Cfap53* | 27.04824 | 5.024882 | 6.96E-05 |
| ENSMUSG00000045216 | *Hs6st1* | 6357.098 | 3.120928 | 7.04E-05 |
| ENSMUSG00000034729 | *Mrps10* | 2047.193 | 6.974238 | 7.06E-05 |
| ENSMUSG00000022508 | *Bcl6* | 831.968 | 5.287342 | 7.14E-05 |
| ENSMUSG00000030643 | *Rab30* | 184.4552 | 7.004299 | 7.26E-05 |
| ENSMUSG00000039917 | *Rhbdd2* | 1782.437 | 5.589787 | 7.26E-05 |
| ENSMUSG00000071984 | *Fndc1* | 74.88121 | 5.737576 | 7.26E-05 |
| ENSMUSG00000025912 | *Mybl1* | 30.94343 | 5.588885 | 7.30E-05 |
| ENSMUSG00000061119 | *Prcp* | 374.8813 | 5.839242 | 7.32E-05 |
| ENSMUSG00000105645 | *Gm6649* | 72.49767 | 6.67243 | 7.39E-05 |
| ENSMUSG00000032089 | *Il10ra* | 11.21215 | 4.816849 | 7.43E-05 |
| ENSMUSG00000038143 | *Stox2* | 1048.204 | 6.930675 | 7.45E-05 |
| ENSMUSG00000017734 | *Dbndd2* | 127.0617 | 7.42242 | 7.67E-05 |
| ENSMUSG00000036136 | *Fam110c* | 225.1301 | 6.314315 | 8.04E-05 |
| ENSMUSG00000029616 | *Erp29* | 7123.588 | 5.063031 | 8.22E-05 |
| ENSMUSG00000025873 | *Faf2* | 10337.6 | 5.387745 | 8.44E-05 |
| ENSMUSG00000075023 | *Accsl* | 28.61191 | 5.988922 | 8.52E-05 |
| ENSMUSG00000008384 | *Sertad1* | 38.75968 | 6.054994 | 8.58E-05 |
| ENSMUSG00000029334 | *Prkg2* | 78.25024 | 6.227343 | 8.68E-05 |
| ENSMUSG00000061353 | *Cxcl12* | 843.7165 | 6.623855 | 8.68E-05 |
| ENSMUSG00000020307 | *Cdc34* | 5127.167 | 6.231156 | 8.74E-05 |
| ENSMUSG00000054951 | *9130008F23Rik* | 80.60499 | 5.465338 | 8.79E-05 |
| ENSMUSG00000049313 | *Sorl1* | 4606.024 | 5.925259 | 8.83E-05 |
| ENSMUSG00000034981 | *Parm1* | 7722.346 | 4.875284 | 9.11E-05 |
| ENSMUSG00000093402 | *Gm18588* | 19.12017 | 5.496002 | 9.13E-05 |
| ENSMUSG00000025348 | *Itga7* | 255.5272 | 7.242556 | 9.13E-05 |
| ENSMUSG00000022206 | *Npr3* | 68.34692 | 5.336825 | 9.27E-05 |
| ENSMUSG00000044934 | *Zfp367* | 1249.391 | 7.070865 | 9.29E-05 |
| ENSMUSG00000086580 | *Gm15280* | 2111.485 | 5.955902 | 9.34E-05 |
| ENSMUSG00000005871 | *Apc* | 7623.372 | 4.696098 | 9.39E-05 |
| ENSMUSG00000030532 | *Hddc3* | 15495.35 | 4.233186 | 9.42E-05 |
| ENSMUSG00000038695 | *Josd2* | 3204.256 | 5.654521 | 9.43E-05 |
| ENSMUSG00000052364 | *B630019K06Rik* | 143.6935 | 5.714516 | 9.60E-05 |
| ENSMUSG00000029592 | *Usp30* | 4593.81 | 6.560557 | 9.61E-05 |
| ENSMUSG00000000531 | *Grasp* | 2658.403 | 5.823654 | 9.66E-05 |
| ENSMUSG00000034271 | *Jdp2* | 87.6128 | 6.514361 | 9.66E-05 |
| ENSMUSG00000035311 | *Gnptab* | 3418.43 | 6.145263 | 9.86E-05 |
| ENSMUSG00000004891 | *Nes* | 94.10601 | 7.196981 | 1.01E-04 |
| ENSMUSG00000085787 | *Gm13092* | 78.50563 | 5.284909 | 1.02E-04 |
| ENSMUSG00000078901 | *Gm14440* | 25.79061 | 5.953456 | 1.04E-04 |
| ENSMUSG00000028949 | *Smarcd3* | 3073.5 | 5.610275 | 1.05E-04 |
| ENSMUSG00000027254 | *Map1a* | 5267.601 | 5.860882 | 1.08E-04 |
| ENSMUSG00000022091 | *Sorbs3* | 6410.635 | 5.442632 | 1.09E-04 |
| ENSMUSG00000054770 | *Kctd18* | 631.6646 | 6.214774 | 1.09E-04 |
| ENSMUSG00000038535 | *Zfp280d* | 4830.514 | 6.314627 | 1.10E-04 |
| ENSMUSG00000054455 | *Vapb* | 4748.191 | 4.794934 | 1.11E-04 |
| ENSMUSG00000001380 | *Hars* | 1469.36 | 5.833123 | 1.12E-04 |
| ENSMUSG00000050503 | *Fbxl22* | 6332.129 | 5.947646 | 1.13E-04 |
| ENSMUSG00000024795 | *Kif20b* | 526.6021 | 5.728034 | 1.15E-04 |
| ENSMUSG00000041012 | *Cmtm8* | 24.94114 | 5.875616 | 1.15E-04 |
| ENSMUSG00000030748 | *Il4ra* | 3650.935 | 6.628992 | 1.15E-04 |
| ENSMUSG00000029610 | *Aimp2* | 1267.699 | 6.281632 | 1.15E-04 |
| ENSMUSG00000053519 | *Kcnip1* | 55.66474 | 5.03352 | 1.15E-04 |
| ENSMUSG00000107724 | *Gm16042* | 92.43718 | 6.848011 | 1.16E-04 |
| ENSMUSG00000049354 | *Dcaf7* | 3734.239 | 4.919615 | 1.17E-04 |
| ENSMUSG00000057551 | *Zfp317* | 555.6081 | 6.245246 | 1.17E-04 |
| ENSMUSG00000013707 | *Tnfaip8l2* | 10.99384 | 4.562175 | 1.18E-04 |
| ENSMUSG00000025372 | *Baiap2* | 2114.849 | 6.075086 | 1.18E-04 |
| ENSMUSG00000029310 | *Nudt9* | 1201.739 | 6.431307 | 1.20E-04 |
| ENSMUSG00000037805 | *Rpl10a* | 3577.006 | 5.803803 | 1.23E-04 |
| ENSMUSG00000022450 | *Ndufa6* | 985.1144 | 5.789527 | 1.23E-04 |
| ENSMUSG00000027931 | *Npr1* | 7105.211 | 5.62553 | 1.23E-04 |
| ENSMUSG00000036499 | *Eea1* | 1330.508 | 5.725108 | 1.24E-04 |
| ENSMUSG00000052397 | *Ezr* | 15141.95 | 3.471888 | 1.25E-04 |
| ENSMUSG00000047881 | *Rell1* | 536.404 | 7.280392 | 1.27E-04 |
| ENSMUSG00000059263 | *Usp47* | 2654.069 | 5.938782 | 1.30E-04 |
| ENSMUSG00000028328 | *Tmod1* | 127.5349 | 6.061345 | 1.30E-04 |
| ENSMUSG00000050150 | *Slc9b1* | 16.69138 | 5.038176 | 1.35E-04 |
| ENSMUSG00000038291 | *Snx25* | 835.283 | 5.839912 | 1.35E-04 |
| ENSMUSG00000061024 | *Rrs1* | 5649.228 | 5.530418 | 1.37E-04 |
| ENSMUSG00000001025 | *S100a6* | 324.766 | 6.253207 | 1.39E-04 |
| ENSMUSG00000001583 | *Tnk1* | 40.05109 | 5.602421 | 1.39E-04 |
| ENSMUSG00000027374 | *Mrps5* | 4414.392 | 5.657966 | 1.41E-04 |
| ENSMUSG00000020644 | *Id2* | 460.8191 | 5.96474 | 1.42E-04 |
| ENSMUSG00000026024 | *Als2* | 4945.787 | 7.284214 | 1.42E-04 |
| ENSMUSG00000014769 | *Psmb1* | 2584.848 | 5.693844 | 1.44E-04 |
| ENSMUSG00000020949 | *Fkbp3* | 266.6484 | 5.794576 | 1.46E-04 |
| ENSMUSG00000020894 | *Vamp2* | 3420.968 | 5.395129 | 1.49E-04 |
| ENSMUSG00000024664 | *Fads3* | 352.2905 | 5.387623 | 1.50E-04 |
| ENSMUSG00000085272 | *Sbk3* | 25.5893 | 5.070863 | 1.53E-04 |
| ENSMUSG00000098832 | *Kdm4dl* | 197.0185 | 6.132385 | 1.55E-04 |
| ENSMUSG00000043241 | *Upf2* | 8543.743 | 4.81962 | 1.56E-04 |
| ENSMUSG00000019970 | *Sgk1* | 10185.49 | 5.049496 | 1.60E-04 |
| ENSMUSG00000059776 | *Rpl13* | 251.7243 | 6.593303 | 1.62E-04 |
| ENSMUSG00000041481 | *Serpina3g* | 13.78602 | 5.122764 | 1.63E-04 |
| ENSMUSG00000070738 | *Dgkd* | 1786.699 | 4.6178 | 1.64E-04 |
| ENSMUSG00000041544 | *Disp3* | 126.8626 | 6.400498 | 1.64E-04 |
| ENSMUSG00000034452 | *Slc24a1* | 353.8096 | 6.907779 | 1.67E-04 |
| ENSMUSG00000020627 | *Klhl29* | 14.37958 | 4.936339 | 1.68E-04 |
| ENSMUSG00000061136 | *Prpf40a* | 1256.295 | 5.089676 | 1.69E-04 |
| ENSMUSG00000035314 | *Gdpd5* | 1095.301 | 5.790263 | 1.70E-04 |
| ENSMUSG00000027459 | *Fam110a* | 450.617 | 6.041756 | 1.70E-04 |
| ENSMUSG00000004655 | *Aqp1* | 23.44695 | 5.070823 | 1.71E-04 |
| ENSMUSG00000026728 | *Vim* | 11127.04 | 3.879686 | 1.75E-04 |
| ENSMUSG00000020116 | *Pno1* | 67.28465 | 5.393077 | 1.75E-04 |
| ENSMUSG00000044770 | *Scml4* | 133.1259 | 6.734486 | 1.81E-04 |
| ENSMUSG00000080242 | *Gm15487* | 332.9836 | 7.751344 | 1.82E-04 |
| ENSMUSG00000057156 | *Homez* | 1269.787 | 7.321641 | 1.84E-04 |
| ENSMUSG00000027932 | *Slc27a3* | 3139.297 | 5.527851 | 1.85E-04 |
| ENSMUSG00000028221 | *Tmem55a* | 567.0991 | 6.410294 | 1.88E-04 |
| ENSMUSG00000043998 | *Mgat2* | 3065.145 | 5.914686 | 1.90E-04 |
| ENSMUSG00000001127 | *Araf* | 2764.984 | 4.348798 | 1.91E-04 |
| ENSMUSG00000039910 | *Cited2* | 25195.65 | 3.498424 | 1.91E-04 |
| ENSMUSG00000044288 | *Cnr1* | 253.5737 | 6.594507 | 1.95E-04 |
| ENSMUSG00000079557 | *March2* | 2080.79 | 6.658016 | 1.95E-04 |
| ENSMUSG00000030352 | *Tspan9* | 1536.782 | 5.827236 | 1.96E-04 |
| ENSMUSG00000054115 | *Skp2* | 1030.088 | 5.245791 | 1.97E-04 |
| ENSMUSG00000020023 | *Tmcc3* | 271.03 | 6.909967 | 1.98E-04 |
| ENSMUSG00000075596 | *B130006D01Rik* | 29.20753 | 4.859548 | 1.99E-04 |
| ENSMUSG00000107705 | *Gm45062* | 23.80864 | 5.422671 | 2.01E-04 |
| ENSMUSG00000071713 | *Csf2rb* | 1315.476 | 5.459933 | 2.06E-04 |
| ENSMUSG00000079477 | *Rab7* | 10797.63 | 5.232609 | 2.07E-04 |
| ENSMUSG00000027304 | *Rtf1* | 6227.06 | 4.562346 | 2.10E-04 |
| ENSMUSG00000022871 | *Fetub* | 95.89528 | 4.357911 | 2.11E-04 |
| ENSMUSG00000029111 | *Nelfa* | 1025.039 | 5.76424 | 2.11E-04 |
| ENSMUSG00000034252 | *Senp6* | 1512.266 | 5.314921 | 2.14E-04 |
| ENSMUSG00000034403 | *Pja1* | 5222.411 | 5.771508 | 2.16E-04 |
| ENSMUSG00000042734 | *Ttc9* | 389.2041 | 6.075108 | 2.17E-04 |
| ENSMUSG00000055945 | *Prr18* | 23.36584 | 4.974398 | 2.18E-04 |
| ENSMUSG00000038696 | *Mapkap1* | 2228.975 | 6.586336 | 2.25E-04 |
| ENSMUSG00000029638 | *Glcci1* | 266.4196 | 5.685651 | 2.26E-04 |
| ENSMUSG00000031565 | *Fgfr1* | 3153.852 | 4.595376 | 2.28E-04 |
| ENSMUSG00000021728 | *Emb* | 252.4315 | 5.448792 | 2.36E-04 |
| ENSMUSG00000027276 | *Jag1* | 4727.368 | 5.813482 | 2.40E-04 |
| ENSMUSG00000006445 | *Epha2* | 2118.828 | 8.146628 | 2.43E-04 |
| ENSMUSG00000107068 | *Gm42742* | 1310.852 | 5.506813 | 2.52E-04 |
| ENSMUSG00000022536 | *Glyr1* | 4063.707 | 4.471155 | 2.54E-04 |
| ENSMUSG00000036902 | *Neto2* | 51.21286 | 5.646068 | 2.56E-04 |
| ENSMUSG00000015312 | *Gadd45b* | 949.5155 | 6.060614 | 2.61E-04 |
| ENSMUSG00000073650 | *Catip* | 292.6107 | 6.44262 | 2.62E-04 |
| ENSMUSG00000020598 | *Nrcam* | 1149.72 | 6.305601 | 2.63E-04 |
| ENSMUSG00000040463 | *Mybbp1a* | 20683.86 | 4.541469 | 2.64E-04 |
| ENSMUSG00000034391 | *Fbxo15* | 34.25052 | 5.464838 | 2.64E-04 |
| ENSMUSG00000040387 | *Klhl32* | 16.49229 | 5.306664 | 2.65E-04 |
| ENSMUSG00000032383 | *Ppib* | 7921.729 | 4.792943 | 2.65E-04 |
| ENSMUSG00000031967 | *Afg3l1* | 4125.928 | 4.885855 | 2.67E-04 |
| ENSMUSG00000070934 | *Rraga* | 1135.498 | 5.562867 | 2.69E-04 |
| ENSMUSG00000020656 | *Grhl1* | 13.94653 | 4.670396 | 2.72E-04 |
| ENSMUSG00000029762 | *Akr1b8* | 176.7597 | 6.2809 | 2.72E-04 |
| ENSMUSG00000107023 | *Gm42715* | 30.44237 | 5.127982 | 2.73E-04 |
| ENSMUSG00000079426 | *Arpc4* | 1828.143 | 4.040288 | 2.74E-04 |
| ENSMUSG00000058006 | *Mdn1* | 11448.78 | 3.518503 | 2.76E-04 |
| ENSMUSG00000037185 | *Krt80* | 16.2706 | 5.108017 | 2.77E-04 |
| ENSMUSG00000095286 | *Olfr117* | 15.85656 | 4.603552 | 2.81E-04 |
| ENSMUSG00000067702 | *Tuba3a* | 14.3238 | 4.915417 | 2.82E-04 |
| ENSMUSG00000049436 | *Upk1b* | 22.33938 | 4.9763 | 2.82E-04 |
| ENSMUSG00000062075 | *Lmnb2* | 3397.091 | 5.59903 | 2.84E-04 |
| ENSMUSG00000013089 | *Etv5* | 160.2922 | 6.170065 | 2.84E-04 |
| ENSMUSG00000028851 | *Nudc* | 9683.654 | 4.770967 | 2.87E-04 |
| ENSMUSG00000055044 | *Pdlim1* | 5368.072 | 4.11911 | 2.89E-04 |
| ENSMUSG00000028992 | *Nmnat1* | 1019.14 | 6.278732 | 2.91E-04 |
| ENSMUSG00000037060 | *Cavin3* | 71.95377 | 5.804257 | 2.99E-04 |
| ENSMUSG00000028639 | *Ybx1* | 27402.28 | 3.539348 | 2.99E-04 |
| ENSMUSG00000041028 | *Ghitm* | 4777.525 | 5.683584 | 2.99E-04 |
| ENSMUSG00000036052 | *Dnajb5* | 535.6449 | 6.201227 | 3.02E-04 |
| ENSMUSG00000074909 | *Ranbp6* | 127.3219 | 6.170557 | 3.02E-04 |
| ENSMUSG00000021725 | *Parp8* | 1048.792 | 4.517013 | 3.04E-04 |
| ENSMUSG00000003032 | *Klf4* | 951.0003 | 5.599587 | 3.06E-04 |
| ENSMUSG00000028967 | *Errfi1* | 2328.366 | 5.500427 | 3.06E-04 |
| ENSMUSG00000061462 | *Obscn* | 83.05689 | 5.788776 | 3.16E-04 |
| ENSMUSG00000026532 | *Spta1* | 26.24952 | 5.269798 | 3.17E-04 |
| ENSMUSG00000025855 | *Prkar1b* | 411.9312 | 6.114489 | 3.25E-04 |
| ENSMUSG00000009941 | *Nxf2* | 60.73994 | 4.857428 | 3.27E-04 |
| ENSMUSG00000029802 | *Abcg2* | 236.3958 | 5.44525 | 3.28E-04 |
| ENSMUSG00000028277 | *Ube2j1* | 1190.604 | 6.214867 | 3.28E-04 |
| ENSMUSG00000066568 | *Lsm14a* | 2280.216 | 5.012713 | 3.28E-04 |
| ENSMUSG00000050468 | *Astl* | 46.08649 | 6.222738 | 3.28E-04 |
| ENSMUSG00000024959 | *Bad* | 1861.065 | 6.452673 | 3.34E-04 |
| ENSMUSG00000019564 | *Arid3a* | 2762.163 | 5.840771 | 3.35E-04 |
| ENSMUSG00000018171 | *Vmp1* | 2460.473 | 5.731945 | 3.37E-04 |
| ENSMUSG00000079451 | *Tmprss11g* | 83.20089 | 5.372628 | 3.41E-04 |
| ENSMUSG00000043872 | *Zmym1* | 1884.3 | 7.553308 | 3.52E-04 |
| ENSMUSG00000086583 | *Gm15500* | 8326.903 | 5.092914 | 3.52E-04 |
| ENSMUSG00000059873 | *Olfr1029* | 96.38628 | 5.563959 | 3.56E-04 |
| ENSMUSG00000022040 | *Ephx2* | 36731.17 | 3.840058 | 3.60E-04 |
| ENSMUSG00000038244 | *Mical2* | 3796.924 | 5.34792 | 3.64E-04 |
| ENSMUSG00000074280 | *Gm6166* | 249.209 | 6.986125 | 3.68E-04 |
| ENSMUSG00000019852 | *Arfgef3* | 573.825 | 5.448189 | 3.70E-04 |
| ENSMUSG00000026455 | *Klhl12* | 3403.322 | 6.39326 | 3.71E-04 |
| ENSMUSG00000038970 | *Lmtk2* | 6712.154 | 4.234 | 3.77E-04 |
| ENSMUSG00000018899 | *Irf1* | 1284.709 | 6.157849 | 3.79E-04 |
| ENSMUSG00000109801 | *Olfr1316* | 11.2681 | 3.798233 | 3.80E-04 |
| ENSMUSG00000025423 | *Pias2* | 3329.713 | 5.791402 | 3.86E-04 |
| ENSMUSG00000030088 | *Aldh1l1* | 6084.311 | 5.805459 | 3.92E-04 |
| ENSMUSG00000002068 | *Ccne1* | 16.43539 | 4.287124 | 3.94E-04 |
| ENSMUSG00000034731 | *Dgkh* | 123.1164 | 5.283074 | 3.98E-04 |
| ENSMUSG00000036887 | *C1qa* | 69.42391 | 4.710403 | 4.03E-04 |
| ENSMUSG00000049811 | *Fam161a* | 149.6022 | 5.827643 | 4.06E-04 |
| ENSMUSG00000018846 | *Pank3* | 1276.995 | 4.833429 | 4.07E-04 |
| ENSMUSG00000041073 | *Nacad* | 731.0352 | 5.824578 | 4.11E-04 |
| ENSMUSG00000038651 | *Sycp2l* | 10.33264 | 4.57688 | 4.11E-04 |
| ENSMUSG00000024359 | *Hspa9* | 4830.062 | 4.260347 | 4.13E-04 |
| ENSMUSG00000051146 | *Camk2n2* | 226.7029 | 6.542789 | 4.15E-04 |
| ENSMUSG00000021774 | *Ube2e1* | 487.298 | 5.747084 | 4.19E-04 |
| ENSMUSG00000039515 | *Ptpa* | 5395.312 | 5.18389 | 4.19E-04 |
| ENSMUSG00000051223 | *Bzw1* | 2920.3 | 3.635124 | 4.20E-04 |
| ENSMUSG00000057858 | *Fam204a* | 566.8094 | 6.261045 | 4.21E-04 |
| ENSMUSG00000004044 | *Cavin1* | 5941.922 | 3.984352 | 4.24E-04 |
| ENSMUSG00000020783 | *Ncbp3* | 3223.261 | 4.699772 | 4.34E-04 |
| ENSMUSG00000071604 | *Fam189a2* | 15.1015 | 4.709901 | 4.37E-04 |
| ENSMUSG00000009687 | *Fxyd5* | 1083.137 | 6.631996 | 4.38E-04 |
| ENSMUSG00000043542 | *Zc2hc1a* | 75.44311 | 5.507026 | 4.38E-04 |
| ENSMUSG00000031600 | *Vps37a* | 4453.393 | 5.15621 | 4.39E-04 |
| ENSMUSG00000032307 | *Ube2q2* | 207.8804 | 4.656971 | 4.41E-04 |
| ENSMUSG00000026174 | *Cnot9* | 1949.18 | 6.222116 | 4.45E-04 |
| ENSMUSG00000071644 | *Eef1g* | 9057.179 | 5.028695 | 4.51E-04 |
| ENSMUSG00000015970 | *Chdh* | 140.1046 | 6.530912 | 4.54E-04 |
| ENSMUSG00000005125 | *Ndrg1* | 431.6425 | 6.119247 | 4.68E-04 |
| ENSMUSG00000071317 | *Bves* | 31.04875 | 5.126478 | 4.70E-04 |
| ENSMUSG00000020614 | *Fam20a* | 25.10219 | 4.134705 | 4.86E-04 |
| ENSMUSG00000047141 | *Zfp654* | 841.5154 | 5.143835 | 4.89E-04 |
| ENSMUSG00000022641 | *Bbx* | 3709.854 | 4.401971 | 4.90E-04 |
| ENSMUSG00000059920 | *4930453N24Rik* | 84.75521 | 4.710586 | 4.92E-04 |
| ENSMUSG00000028060 | *2810403A07Rik* | 2126.059 | 4.993867 | 4.95E-04 |
| ENSMUSG00000028948 | *Nol9* | 934.7736 | 4.391534 | 4.96E-04 |
| ENSMUSG00000027195 | *Hsd17b12* | 1060.982 | 5.136236 | 4.97E-04 |
| ENSMUSG00000073411 | *H2* | 9244.215 | 4.333156 | 4.98E-04 |
| ENSMUSG00000029338 | *Antxr2* | 1319.883 | 5.496682 | 5.01E-04 |
| ENSMUSG00000037815 | *Ctnna1* | 7591.63 | 4.063887 | 5.07E-04 |
| ENSMUSG00000062234 | *Gak* | 9395.869 | 4.585486 | 5.12E-04 |
| ENSMUSG00000061186 | *Sfmbt2* | 21.1818 | 4.729839 | 5.15E-04 |
| ENSMUSG00000039068 | *Zzz3* | 4057.955 | 4.872307 | 5.16E-04 |
| ENSMUSG00000037855 | *Zfp365* | 1450.19 | 6.658097 | 5.16E-04 |
| ENSMUSG00000062762 | *Ei24* | 1758.178 | 5.319948 | 5.18E-04 |
| ENSMUSG00000037493 | *Cib2* | 25.38546 | 4.713914 | 5.20E-04 |
| ENSMUSG00000035129 | *Gm6781* | 7.317528 | 4.000823 | 5.24E-04 |
| ENSMUSG00000031530 | *Dusp4* | 1336.1 | 6.698079 | 5.29E-04 |
| ENSMUSG00000039252 | *Lgi2* | 14.65607 | 4.311043 | 5.42E-04 |
| ENSMUSG00000041355 | *Ssr2* | 1613.897 | 4.624819 | 5.53E-04 |
| ENSMUSG00000021067 | *Sav1* | 1442.402 | 5.498947 | 5.55E-04 |
| ENSMUSG00000017747 | *Ghdc* | 1448.539 | 6.466678 | 5.57E-04 |
| ENSMUSG00000082596 | *Gm14227* | 12.32933 | 5.067412 | 5.64E-04 |
| ENSMUSG00000050299 | *Gm9843* | 3048.377 | 4.769363 | 5.66E-04 |
| ENSMUSG00000021759 | *Plpp1* | 57.34565 | 4.741257 | 5.66E-04 |
| ENSMUSG00000025034 | *Trim8* | 8203.87 | 3.529943 | 5.69E-04 |
| ENSMUSG00000031960 | *Aars* | 8935.018 | 3.452886 | 5.70E-04 |
| ENSMUSG00000093385 | *A330044P14Rik* | 52.73862 | 5.357109 | 5.71E-04 |
| ENSMUSG00000036371 | *Serbp1* | 18057.72 | 3.522606 | 5.76E-04 |
| ENSMUSG00000028063 | *Lmna* | 6392.229 | 4.085164 | 5.79E-04 |
| ENSMUSG00000023868 | *Pde10a* | 1120.946 | 5.142505 | 5.81E-04 |
| ENSMUSG00000048285 | *Frmd6* | 1246.772 | 5.659052 | 5.86E-04 |
| ENSMUSG00000024907 | *Gal* | 7.140005 | 3.754482 | 5.86E-04 |
| ENSMUSG00000046108 | *Il17c* | 10.06911 | 4.099597 | 5.89E-04 |
| ENSMUSG00000028431 | *Ikbkap* | 1998.47 | 5.272108 | 5.90E-04 |
| ENSMUSG00000021217 | *Tshz3* | 12.0623 | 4.751209 | 5.95E-04 |
| ENSMUSG00000063410 | *Stk24* | 2331.101 | 4.311357 | 6.00E-04 |
| ENSMUSG00000092586 | *Ly6g6c* | 90.42321 | 5.251221 | 6.01E-04 |
| ENSMUSG00000002455 | *Prpf6* | 2183.674 | 4.79197 | 6.01E-04 |
| ENSMUSG00000030123 | *Plxnd1* | 5018.386 | 5.083536 | 6.02E-04 |
| ENSMUSG00000048007 | *Timm8a1* | 22.12885 | 4.574479 | 6.05E-04 |
| ENSMUSG00000100204 | *Gm4849* | 14.03098 | 4.585758 | 6.05E-04 |
| ENSMUSG00000047638 | *Nr1h4* | 10.10641 | 3.889101 | 6.06E-04 |
| ENSMUSG00000050592 | *Fam78a* | 2529.898 | 4.726161 | 6.09E-04 |
| ENSMUSG00000030560 | *Ctsc* | 145.3769 | 5.95123 | 6.13E-04 |
| ENSMUSG00000031428 | *Zcchc18* | 7.991344 | 4.161606 | 6.14E-04 |
| ENSMUSG00000021288 | *Klc1* | 16084.48 | 4.382195 | 6.20E-04 |
| ENSMUSG00000028309 | *Rnf20* | 5634.386 | 4.637704 | 6.23E-04 |
| ENSMUSG00000044709 | *Gemin7* | 983.2033 | 5.535064 | 6.25E-04 |
| ENSMUSG00000003360 | *Ddx23* | 12671.25 | 4.455075 | 6.31E-04 |
| ENSMUSG00000025921 | *Rdh10* | 83.10008 | 5.649403 | 6.32E-04 |
| ENSMUSG00000025647 | *Shisa5* | 4845.977 | 4.090308 | 6.36E-04 |
| ENSMUSG00000054450 | *Gm9945* | 47.85459 | 5.884888 | 6.40E-04 |
| ENSMUSG00000042167 | *Papd4* | 628.0824 | 5.739504 | 6.42E-04 |
| ENSMUSG00000022516 | *Nudt16l1* | 666.5595 | 6.20513 | 6.47E-04 |
| ENSMUSG00000021614 | *Vcan* | 42296.08 | 3.172045 | 6.56E-04 |
| ENSMUSG00000075012 | *Fjx1* | 109.3069 | 6.020444 | 6.65E-04 |
| ENSMUSG00000040459 | *Arglu1* | 4827.093 | 4.411429 | 6.72E-04 |
| ENSMUSG00000072235 | *Tuba1a* | 36699.75 | 4.02478 | 6.77E-04 |
| ENSMUSG00000054843 | *Atrnl1* | 4387.958 | 4.953823 | 6.81E-04 |
| ENSMUSG00000046731 | *Kctd11* | 406.1755 | 6.30025 | 6.89E-04 |
| ENSMUSG00000039105 | *Atp6v1g1* | 444.0862 | 5.253851 | 6.91E-04 |
| ENSMUSG00000079003 | *Samd1* | 3493.215 | 5.830778 | 7.00E-04 |
| ENSMUSG00000017999 | *Ddx27* | 4480.134 | 3.943352 | 7.05E-04 |
| ENSMUSG00000004415 | *Col26a1* | 168.4978 | 6.261095 | 7.10E-04 |
| ENSMUSG00000028412 | *Slc44a1* | 4405.22 | 4.633027 | 7.11E-04 |
| ENSMUSG00000020982 | *Nemf* | 2977.052 | 5.022784 | 7.19E-04 |
| ENSMUSG00000020917 | *Acly* | 19472.24 | 3.272333 | 7.39E-04 |
| ENSMUSG00000087006 | *Gm13889* | 1249.632 | 6.342667 | 7.39E-04 |
| ENSMUSG00000038000 | *Acd* | 1691.058 | 5.438352 | 7.40E-04 |
| ENSMUSG00000101660 | *Gm18666* | 112.0393 | 6.28638 | 7.41E-04 |
| ENSMUSG00000068699 | *Flnc* | 1808.02 | 4.956728 | 7.49E-04 |
| ENSMUSG00000081967 | *Gm14017* | 9.670284 | 3.850218 | 7.53E-04 |
| ENSMUSG00000023235 | *Ccl25* | 600.7176 | 5.199417 | 7.53E-04 |
| ENSMUSG00000010095 | *Slc3a2* | 7947.215 | 3.329502 | 7.71E-04 |
| ENSMUSG00000018428 | *Akap1* | 7142.652 | 4.023291 | 7.77E-04 |
| ENSMUSG00000041075 | *Fzd7* | 2113.727 | 5.832506 | 7.83E-04 |
| ENSMUSG00000032034 | *Kcnj5* | 1111.765 | 5.248273 | 7.95E-04 |
| ENSMUSG00000027774 | *Gfm1* | 6101.57 | 6.427239 | 8.14E-04 |
| ENSMUSG00000006740 | *Kif5b* | 3619.485 | 4.464109 | 8.16E-04 |
| ENSMUSG00000061607 | *Mdc1* | 3312.954 | 4.088612 | 8.32E-04 |
| ENSMUSG00000038578 | *Susd1* | 2968.685 | 4.523796 | 8.37E-04 |
| ENSMUSG00000028385 | *Snx30* | 85.63725 | 5.756073 | 8.39E-04 |
| ENSMUSG00000035045 | *Zc3h12b* | 297.5099 | 5.674965 | 8.52E-04 |
| ENSMUSG00000060548 | *Tnfrsf19* | 15.68636 | 4.346379 | 8.52E-04 |
| ENSMUSG00000038528 | *Mfsd4b5* | 4689.015 | 5.038148 | 8.52E-04 |
| ENSMUSG00000021903 | *Galnt15* | 2351.672 | 5.192247 | 8.53E-04 |
| ENSMUSG00000041124 | *Msantd4* | 1330.465 | 4.218679 | 8.54E-04 |
| ENSMUSG00000042712 | *Tceal9* | 14308.13 | 3.64007 | 8.72E-04 |
| ENSMUSG00000001569 | *Nom1* | 541.1567 | 4.605532 | 8.72E-04 |
| ENSMUSG00000019362 | *D8Ertd738e* | 774.2444 | 5.870519 | 8.82E-04 |
| ENSMUSG00000025743 | *Sdc3* | 4199.001 | 4.164241 | 8.87E-04 |
| ENSMUSG00000062580 | *Timm17a* | 476.7107 | 5.243932 | 8.90E-04 |
| ENSMUSG00000032412 | *Atp1b3* | 5083.204 | 5.228542 | 8.90E-04 |
| ENSMUSG00000092284 | *Gm8801* | 82.38476 | 5.756061 | 8.97E-04 |
| ENSMUSG00000012350 | *Ehf* | 200.4173 | 6.490839 | 9.08E-04 |
| ENSMUSG00000022416 | *Cacna1i* | 33.87503 | 4.954029 | 9.25E-04 |
| ENSMUSG00000028392 | *Bspry* | 252.945 | 5.543307 | 9.27E-04 |
| ENSMUSG00000038024 | *Dennd4c* | 3475.932 | 4.966998 | 9.27E-04 |
| ENSMUSG00000109511 | *Nup62* | 5104.149 | 4.64784 | 9.32E-04 |
| ENSMUSG00000046388 | *Gm9806* | 63.46575 | 5.609118 | 9.35E-04 |
| ENSMUSG00000064137 | *Rhox8* | 50608.72 | 4.413574 | 9.43E-04 |
| ENSMUSG00000019989 | *Enpp3* | 625.9145 | 5.365846 | 9.55E-04 |
| ENSMUSG00000038845 | *Phb* | 4568.283 | 5.01979 | 9.56E-04 |
| ENSMUSG00000020420 | *Zfp607a* | 112.4975 | 6.155158 | 9.68E-04 |
| ENSMUSG00000023826 | *Park2* | 13.5336 | 4.156609 | 9.77E-04 |
| ENSMUSG00000022814 | *Umps* | 2131.859 | 4.907782 | 9.78E-04 |
| ENSMUSG00000024853 | *Sf3b2* | 4246.905 | 3.420628 | 9.86E-04 |
| ENSMUSG00000029151 | *Slc30a3* | 221.676 | 4.724872 | 9.88E-04 |
| ENSMUSG00000040029 | *Ipo8* | 6880.857 | 5.108113 | 9.95E-04 |
| ENSMUSG00000023018 | *Smarcd1* | 1567.166 | 4.794532 | 9.97E-04 |
| ENSMUSG00000028522 | *Mier1* | 8864.444 | 5.203434 | 0.001023 |
| ENSMUSG00000095929 | *Olfr487* | 9.290867 | 3.780955 | 0.001041 |
| ENSMUSG00000067722 | *BC003965* | 2103.762 | 4.661168 | 0.001045 |
| ENSMUSG00000071172 | *Srsf3* | 1765.024 | 3.831515 | 0.001057 |
| ENSMUSG00000042678 | *Myo15* | 18.97378 | 4.690713 | 0.001069 |
| ENSMUSG00000045636 | *Mtus1* | 543.5469 | 5.126316 | 0.001075 |
| ENSMUSG00000024713 | *Pcsk5* | 8796.503 | 2.887672 | 0.001081 |
| ENSMUSG00000039137 | *Whrn* | 1749.487 | 4.748993 | 0.001083 |
| ENSMUSG00000021431 | *Snrnp48* | 2141.548 | 4.944158 | 0.001083 |
| ENSMUSG00000082674 | *Gm11914* | 122.2456 | 5.365055 | 0.001095 |
| ENSMUSG00000053253 | *Ndfip2* | 1415.659 | 5.673639 | 0.001096 |
| ENSMUSG00000026553 | *Copa* | 10918.42 | 4.099104 | 0.001098 |
| ENSMUSG00000114456 | *Hist1h2bh* | 4870.796 | 4.13624 | 0.001103 |
| ENSMUSG00000063888 | *Rpl7l1* | 3242.795 | 5.465695 | 0.001112 |
| ENSMUSG00000021365 | *Nedd9* | 5493.573 | 4.221585 | 0.001113 |
| ENSMUSG00000078365 | *Mos* | 56.34402 | 5.458161 | 0.001124 |
| ENSMUSG00000049550 | *Clip1* | 5058.313 | 3.790948 | 0.00113 |
| ENSMUSG00000029314 | *Gpat3* | 218.368 | 6.15575 | 0.001141 |
| ENSMUSG00000026547 | *Tagln2* | 9233.245 | 4.846156 | 0.001147 |
| ENSMUSG00000072872 | *Rybp* | 1355.025 | 4.771576 | 0.001158 |
| ENSMUSG00000089716 | *Gm6264* | 931.4304 | 6.412279 | 0.001164 |
| ENSMUSG00000074918 | *Inafm2* | 185.6848 | 4.622479 | 0.001166 |
| ENSMUSG00000024104 | *Washc2* | 5298.712 | 4.831436 | 0.001174 |
| ENSMUSG00000029675 | *Eln* | 489.5208 | 4.902834 | 0.001182 |
| ENSMUSG00000001995 | *Sipa1l2* | 7029.051 | 5.158086 | 0.001183 |
| ENSMUSG00000078490 | *Cfap74* | 33.0084 | 4.672787 | 0.001193 |
| ENSMUSG00000035051 | *Dhx57* | 2350.054 | 4.284801 | 0.001193 |
| ENSMUSG00000006050 | *Sra1* | 1584.671 | 4.431847 | 0.001196 |
| ENSMUSG00000038774 | *Ascc3* | 1965.308 | 4.777425 | 0.001215 |
| ENSMUSG00000013539 | *Tango2* | 395.2188 | 5.265214 | 0.001215 |
| ENSMUSG00000034158 | *Lrrc58* | 4395.936 | 3.060213 | 0.001218 |
| ENSMUSG00000020463 | *Ppp4r3b* | 929.2132 | 4.918826 | 0.00122 |
| ENSMUSG00000019797 | *1700021F05Rik* | 1427.713 | 5.87329 | 0.001223 |
| ENSMUSG00000027601 | *Mtfr1* | 4640.202 | 5.575659 | 0.001227 |
| ENSMUSG00000031575 | *Ash2l* | 3511.128 | 5.096877 | 0.001231 |
| ENSMUSG00000041046 | *Ramp3* | 8.280786 | 3.874706 | 0.001258 |
| ENSMUSG00000036478 | *Btg1* | 864.5514 | 4.925617 | 0.001263 |
| ENSMUSG00000018666 | *Cbx1* | 1587.725 | 4.140971 | 0.001271 |
| ENSMUSG00000022883 | *Robo1* | 15546.3 | 3.121004 | 0.001272 |
| ENSMUSG00000021822 | *Plau* | 9497.393 | 4.2144 | 0.001274 |
| ENSMUSG00000027665 | *Pik3ca* | 845.8637 | 4.369068 | 0.001276 |
| ENSMUSG00000033581 | *Igf2bp2* | 992.0711 | 6.361693 | 0.00128 |
| ENSMUSG00000001065 | *Zfp276* | 3137.353 | 5.452484 | 0.001286 |
| ENSMUSG00000026604 | *Ptpn14* | 3851.876 | 5.207727 | 0.001291 |
| ENSMUSG00000059552 | *Trp53* | 4474.518 | 4.935326 | 0.001301 |
| ENSMUSG00000104651 | *Gm21168* | 5954.601 | 4.224171 | 0.001307 |
| ENSMUSG00000030844 | *Rgs10* | 122.2335 | 6.181658 | 0.00132 |
| ENSMUSG00000029309 | *Sparcl1* | 2258.066 | 5.904078 | 0.001328 |
| ENSMUSG00000045281 | *Gpr20* | 6.556968 | 3.75642 | 0.001329 |
| ENSMUSG00000024841 | *Eif1ad* | 1703.753 | 4.50656 | 0.00133 |
| ENSMUSG00000047619 | *Ddi1* | 355.0584 | 5.565128 | 0.001332 |
| ENSMUSG00000075199 | *Olfr52* | 8.510979 | 3.838402 | 0.001332 |
| ENSMUSG00000032373 | *Car12* | 354.7689 | 5.37529 | 0.001334 |
| ENSMUSG00000025130 | *P4hb* | 16544.19 | 3.019361 | 0.001335 |
| ENSMUSG00000031734 | *Irx3* | 14394.56 | 5.793693 | 0.001343 |
| ENSMUSG00000041476 | *Smpx* | 5.899681 | 3.496382 | 0.001346 |
| ENSMUSG00000029076 | *Sdf4* | 1973.004 | 4.383042 | 0.001351 |
| ENSMUSG00000030304 | *Ergic2* | 1011.721 | 4.792654 | 0.001362 |
| ENSMUSG00000068566 | *Myadm* | 2368.98 | 4.232784 | 0.001363 |
| ENSMUSG00000073295 | *Nudt11* | 71.64495 | 4.382147 | 0.001365 |
| ENSMUSG00000041229 | *Phf8* | 3995.609 | 5.145085 | 0.001369 |
| ENSMUSG00000035621 | *Midn* | 7144.661 | 2.998596 | 0.001369 |
| ENSMUSG00000026965 | *Anapc2* | 13716.65 | 4.675765 | 0.001387 |
| ENSMUSG00000019087 | *Atp6ap1* | 3902.322 | 3.848756 | 0.001398 |
| ENSMUSG00000019814 | *Ltv1* | 740.4361 | 5.586406 | 0.001406 |
| ENSMUSG00000029106 | *Add1* | 9954.603 | 4.557855 | 0.001412 |
| ENSMUSG00000022003 | *Slc25a30* | 4597.673 | 3.520769 | 0.001418 |
| ENSMUSG00000024339 | *Tap2* | 2494.612 | 5.26101 | 0.001425 |
| ENSMUSG00000032501 | *Trib1* | 297.0031 | 4.307491 | 0.001432 |
| ENSMUSG00000055296 | *Tmem245* | 6762.301 | 3.860942 | 0.001432 |
| ENSMUSG00000090486 | *BC035947* | 6.705117 | 3.680267 | 0.001441 |
| ENSMUSG00000087687 | *Pet100* | 733.1884 | 5.652063 | 0.001444 |
| ENSMUSG00000000552 | *Zfp385a* | 412.925 | 4.719163 | 0.001448 |
| ENSMUSG00000036564 | *Ndrg4* | 122.3487 | 5.576884 | 0.001462 |
| ENSMUSG00000027829 | *Ccnl1* | 3602.297 | 4.733089 | 0.001462 |
| ENSMUSG00000036036 | *Zfp57* | 173.0578 | 5.220941 | 0.001465 |
| ENSMUSG00000034486 | *Gbx2* | 6.391968 | 3.699215 | 0.001476 |
| ENSMUSG00000019738 | *Polr2i* | 2241.001 | 5.294143 | 0.001478 |
| ENSMUSG00000029433 | *Diablo* | 2024.434 | 5.075379 | 0.001488 |
| ENSMUSG00000070056 | *Mfhas1* | 417.2727 | 5.246245 | 0.001492 |
| ENSMUSG00000047242 | *Taf9b* | 114.2947 | 4.862316 | 0.001495 |
| ENSMUSG00000067219 | *Nipal1* | 791.4677 | 4.346927 | 0.001503 |
| ENSMUSG00000030763 | *Lcmt1* | 366.1377 | 5.954675 | 0.001512 |
| ENSMUSG00000038534 | *Osbpl7* | 2185.849 | 5.124141 | 0.001523 |
| ENSMUSG00000025880 | *Smad7* | 3613.045 | 4.870494 | 0.001539 |
| ENSMUSG00000000290 | *Itgb2* | 27.71438 | 4.83572 | 0.001541 |
| ENSMUSG00000025278 | *Flnb* | 10961.28 | 4.383094 | 0.001564 |
| ENSMUSG00000032135 | *Mcam* | 59.63234 | 4.108734 | 0.001568 |
| ENSMUSG00000051379 | *Flrt3* | 441.3895 | 4.925525 | 0.001577 |
| ENSMUSG00000043895 | *S1pr2* | 666.5751 | 5.556939 | 0.001586 |
| ENSMUSG00000042428 | *Mgat3* | 26.92916 | 4.892684 | 0.001587 |
| ENSMUSG00000036553 | *Sh3tc1* | 75.28524 | 4.543587 | 0.001594 |
| ENSMUSG00000085795 | *Zfp703* | 1572.674 | 4.666359 | 0.001595 |
| ENSMUSG00000050635 | *Sprr2f* | 6.896551 | 3.378446 | 0.001607 |
| ENSMUSG00000027878 | *Notch2* | 48462.66 | 2.846706 | 0.00161 |
| ENSMUSG00000043099 | *Hic1* | 25.26279 | 4.795086 | 0.001611 |
| ENSMUSG00000025402 | *Nab2* | 1774.931 | 4.611125 | 0.001613 |
| ENSMUSG00000035177 | *Nlrp2* | 44.67756 | 5.555457 | 0.00162 |
| ENSMUSG00000006378 | *Gcat* | 16.51485 | 4.256087 | 0.001643 |
| ENSMUSG00000030117 | *Gdf3* | 6.34488 | 3.115137 | 0.001657 |
| ENSMUSG00000041147 | *Brca2* | 1790.829 | 5.09281 | 0.001657 |
| ENSMUSG00000058773 | *Hist1h1b* | 759.5687 | 4.02998 | 0.001665 |
| ENSMUSG00000055725 | *Paqr3* | 2236.941 | 5.323 | 0.00167 |
| ENSMUSG00000057604 | *Lmcd1* | 21.25102 | 4.606638 | 0.001681 |
| ENSMUSG00000030096 | *Slc6a6* | 32328.81 | 2.471278 | 0.001709 |
| ENSMUSG00000007682 | *Dio2* | 228.5741 | 5.812373 | 0.001709 |
| ENSMUSG00000022711 | *Pmm2* | 4746.084 | 5.379298 | 0.001711 |
| ENSMUSG00000041168 | *Lonp1* | 12576.69 | 3.862995 | 0.001731 |
| ENSMUSG00000042444 | *Mindy2* | 1700.747 | 5.658255 | 0.001733 |
| ENSMUSG00000071645 | *Tut1* | 1682.56 | 4.858838 | 0.001735 |
| ENSMUSG00000041837 | *Pdcd7* | 250.6456 | 5.151358 | 0.001739 |
| ENSMUSG00000029510 | *Gpc2* | 368.8161 | 4.733388 | 0.001748 |
| ENSMUSG00000029404 | *Arl6ip4* | 5179.73 | 4.499958 | 0.001749 |
| ENSMUSG00000030662 | *Ipo5* | 5643.597 | 3.538472 | 0.00175 |
| ENSMUSG00000003380 | *Rabac1* | 949.9362 | 3.915702 | 0.00178 |
| ENSMUSG00000037916 | *Ndufv1* | 3047.576 | 4.314872 | 0.001789 |
| ENSMUSG00000022526 | *Zfp251* | 607.1186 | 4.257452 | 0.001789 |
| ENSMUSG00000054134 | *Umodl1* | 218.1993 | 5.541658 | 0.001793 |
| ENSMUSG00000034850 | *Tmem127* | 2330.893 | 4.503363 | 0.001822 |
| ENSMUSG00000035437 | *Rabgap1* | 1660.901 | 4.902214 | 0.001834 |
| ENSMUSG00000061904 | *Slc25a3* | 5375.11 | 3.347816 | 0.001835 |
| ENSMUSG00000028271 | *Gtf2b* | 814.0083 | 5.703475 | 0.001846 |
| ENSMUSG00000032418 | *Me1* | 8713.907 | 4.197292 | 0.001849 |
| ENSMUSG00000021720 | *Rnf180* | 3111.193 | 4.693824 | 0.001852 |
| ENSMUSG00000061232 | *H2* | 4329.135 | 3.752865 | 0.001856 |
| ENSMUSG00000067736 | *Gm10222* | 3737.728 | 7.647107 | 0.001875 |
| ENSMUSG00000027367 | *Stard7* | 853.0016 | 4.31004 | 0.001876 |
| ENSMUSG00000047714 | *Ppp1r2* | 294.8193 | 5.0627 | 0.00188 |
| ENSMUSG00000073616 | *Cops9* | 211.9921 | 4.733917 | 0.001881 |
| ENSMUSG00000027835 | *Pdcd10* | 33.57722 | 4.824234 | 0.001907 |
| ENSMUSG00000062627 | *Mysm1* | 410.8609 | 3.84429 | 0.001908 |
| ENSMUSG00000047466 | *8030462N17Rik* | 671.5613 | 4.557495 | 0.001917 |
| ENSMUSG00000037351 | *Actr1b* | 6791.323 | 4.746633 | 0.001922 |
| ENSMUSG00000011256 | *Adam19* | 117.2493 | 4.676137 | 0.001927 |
| ENSMUSG00000039114 | *Nrn1* | 8684.838 | 3.795368 | 0.001936 |
| ENSMUSG00000090935 | *Synj2bp* | 1284.65 | 6.074453 | 0.001942 |
| ENSMUSG00000033909 | *Usp36* | 47226.32 | 3.837384 | 0.001948 |
| ENSMUSG00000000776 | *Polr3d* | 1246.784 | 3.820764 | 0.001959 |
| ENSMUSG00000029346 | *Srrd* | 115.6871 | 4.92121 | 0.001963 |
| ENSMUSG00000070473 | *Cldn3* | 8.212533 | 3.876419 | 0.001963 |
| ENSMUSG00000015243 | *Abca1* | 54789.32 | 4.31435 | 0.001965 |
| ENSMUSG00000044937 | *Ttc41* | 20.04678 | 4.391086 | 0.001974 |
| ENSMUSG00000032080 | *Apoa4* | 7542.837 | 4.040416 | 0.001979 |
| ENSMUSG00000055322 | *Tns1* | 6649.471 | 3.774653 | 0.001981 |
| ENSMUSG00000028121 | *Bcar3* | 2735.285 | 4.695029 | 0.001983 |
| ENSMUSG00000034254 | *Agpat1* | 3287.109 | 3.464739 | 0.002 |
| ENSMUSG00000049097 | *Ankrd34a* | 6.496821 | 3.423994 | 0.002002 |
| ENSMUSG00000032802 | *Srxn1* | 307.3022 | 5.674983 | 0.002008 |
| ENSMUSG00000028454 | *Pigo* | 1612.124 | 5.119307 | 0.002011 |
| ENSMUSG00000041506 | *Rrp9* | 2595.274 | 5.437015 | 0.002027 |
| ENSMUSG00000081739 | *Mdm4* | 19.48685 | 4.066437 | 0.002035 |
| ENSMUSG00000015053 | *Gata2* | 21.84324 | 4.344801 | 0.002046 |
| ENSMUSG00000025812 | *Pard3* | 5081.565 | 3.990666 | 0.002067 |
| ENSMUSG00000033855 | *Ston1* | 1625.635 | 4.338492 | 0.002072 |
| ENSMUSG00000034247 | *Plekhm1* | 1430.212 | 4.377337 | 0.002091 |
| ENSMUSG00000056216 | *Cebpg* | 699.5498 | 4.804005 | 0.002094 |
| ENSMUSG00000030209 | *Grin2b* | 781.6506 | 3.867932 | 0.002123 |
| ENSMUSG00000033487 | *Fndc3a* | 3283.102 | 4.472417 | 0.002132 |
| ENSMUSG00000049687 | *Fam109b* | 18.31488 | 4.357063 | 0.002133 |
| ENSMUSG00000029203 | *Ube2k* | 5481.835 | 3.707401 | 0.002141 |
| ENSMUSG00000044786 | *Zfp36* | 2215.89 | 4.721743 | 0.002147 |
| ENSMUSG00000037894 | *H2afz* | 2191.932 | 4.650034 | 0.002152 |
| ENSMUSG00000030657 | *Xylt1* | 822.1782 | 4.660372 | 0.002156 |
| ENSMUSG00000058546 | *Rpl23a* | 6972.535 | 3.164915 | 0.002177 |
| ENSMUSG00000025958 | *Creb1* | 2555.674 | 4.770686 | 0.002197 |
| ENSMUSG00000000628 | *Hk2* | 17490.86 | 4.230499 | 0.002199 |
| ENSMUSG00000032913 | *Lrig2* | 709.4111 | 4.575187 | 0.002216 |
| ENSMUSG00000026615 | *Eprs* | 1636.396 | 3.623172 | 0.002222 |
| ENSMUSG00000046470 | *Sox18* | 48.38038 | 4.854827 | 0.002225 |
| ENSMUSG00000081524 | *Gm11658* | 6.417973 | 3.498175 | 0.002236 |
| ENSMUSG00000037594 | *BC022687* | 99.10932 | 4.896545 | 0.002241 |
| ENSMUSG00000021133 | *Susd6* | 4200.835 | 4.310942 | 0.00225 |
| ENSMUSG00000028563 | *Tm2d1* | 9.339928 | 2.796703 | 0.002256 |
| ENSMUSG00000035357 | *Pdzrn3* | 801.9435 | 5.635818 | 0.002259 |
| ENSMUSG00000022634 | *Yaf2* | 500.0251 | 5.36134 | 0.002278 |
| ENSMUSG00000036845 | *Lin37* | 1023.562 | 5.116916 | 0.002303 |
| ENSMUSG00000025650 | *Col7a1* | 154.1683 | 5.698545 | 0.002313 |
| ENSMUSG00000028234 | *Rps20* | 587.2874 | 4.57486 | 0.002315 |
| ENSMUSG00000032191 | *Bcl2l10* | 57.59479 | 5.28363 | 0.00232 |
| ENSMUSG00000021494 | *Ddx41* | 2616.527 | 4.655906 | 0.002322 |
| ENSMUSG00000050896 | *Rtn4rl2* | 8.328916 | 3.787774 | 0.002324 |
| ENSMUSG00000026271 | *Gpr35* | 103.5779 | 4.403917 | 0.002333 |
| ENSMUSG00000016942 | *Tmprss6* | 10.28929 | 3.445649 | 0.002339 |
| ENSMUSG00000035105 | *Egln3* | 835.066 | 5.550745 | 0.002339 |
| ENSMUSG00000035171 | *1110059E24Rik* | 74.41236 | 5.310131 | 0.00234 |
| ENSMUSG00000068617 | *Efcab1* | 6.856645 | 3.530351 | 0.002368 |
| ENSMUSG00000068335 | *Dok1* | 128.8063 | 4.625724 | 0.002375 |
| ENSMUSG00000026622 | *Nek2* | 844.4437 | 4.918386 | 0.002377 |
| ENSMUSG00000046841 | *Ckap4* | 6247.583 | 3.431744 | 0.002394 |
| ENSMUSG00000026385 | *Dbi* | 984.6976 | 4.856056 | 0.002395 |
| ENSMUSG00000022537 | *Tmem44* | 45.0177 | 4.550326 | 0.002405 |
| ENSMUSG00000028648 | *Ndufs5* | 995.015 | 5.212399 | 0.002405 |
| ENSMUSG00000030771 | *Micalcl* | 6.872012 | 3.716264 | 0.002413 |
| ENSMUSG00000021911 | *Parg* | 604.6732 | 4.405732 | 0.002423 |
| ENSMUSG00000113175 | *Gm9973* | 15076.02 | 4.281758 | 0.002444 |
| ENSMUSG00000032494 | *Tdgf1* | 16.90118 | 3.659924 | 0.002448 |
| ENSMUSG00000043913 | *Ccdc60* | 169.6503 | 5.221065 | 0.002529 |
| ENSMUSG00000017679 | *Ttpal* | 2233.044 | 4.621505 | 0.002549 |
| ENSMUSG00000052730 | *Gm5111* | 14.97709 | 3.624182 | 0.002592 |
| ENSMUSG00000042289 | *Hsd3b7* | 93.72259 | 4.636913 | 0.002599 |
| ENSMUSG00000041301 | *Cftr* | 17.48261 | 4.601993 | 0.00261 |
| ENSMUSG00000047415 | *Gpr68* | 132.5306 | 4.341102 | 0.002612 |
| ENSMUSG00000035183 | *Slc24a5* | 85.67784 | 4.286172 | 0.002618 |
| ENSMUSG00000052419 | *2610001J05Rik* | 86.55244 | 4.237135 | 0.00262 |
| ENSMUSG00000005672 | *Kit* | 73.40855 | 4.973576 | 0.002635 |
| ENSMUSG00000027513 | *Pck1* | 34.48061 | 4.905886 | 0.002646 |
| ENSMUSG00000041141 | *Pnmal1* | 6.737913 | 3.085594 | 0.002669 |
| ENSMUSG00000019139 | *Isyna1* | 10688.19 | 3.194823 | 0.00267 |
| ENSMUSG00000016409 | *Nkap* | 113.9316 | 4.783095 | 0.002673 |
| ENSMUSG00000021621 | *Zcchc9* | 629.5495 | 4.588804 | 0.002686 |
| ENSMUSG00000021264 | *Yy1* | 1438.895 | 3.623463 | 0.002688 |
| ENSMUSG00000032624 | *Eml4* | 3218.498 | 4.681651 | 0.00269 |
| ENSMUSG00000052075 | *1700029F12Rik* | 5.642182 | 3.199861 | 0.002692 |
| ENSMUSG00000024184 | *Pdia2* | 7.927073 | 3.623314 | 0.002702 |
| ENSMUSG00000045763 | *Basp1* | 277.2939 | 5.412591 | 0.002722 |
| ENSMUSG00000016534 | *Lamp2* | 7092.433 | 4.304742 | 0.002738 |
| ENSMUSG00000037410 | *Tbc1d2b* | 1559.841 | 4.71019 | 0.002742 |
| ENSMUSG00000052673 | *Gm9887* | 5.579031 | 3.372912 | 0.002744 |
| ENSMUSG00000057134 | *Ado* | 1689.598 | 4.014484 | 0.002753 |
| ENSMUSG00000032425 | *Zfp949* | 148.7694 | 5.206996 | 0.002761 |
| ENSMUSG00000019849 | *Prep* | 6724.09 | 3.856295 | 0.002772 |
| ENSMUSG00000114114 | *AC154368* | 14.74787 | 3.67302 | 0.00278 |
| ENSMUSG00000041491 | *Cep78* | 255.9656 | 5.099665 | 0.002789 |
| ENSMUSG00000030432 | *Rpl28* | 2057.247 | 3.615434 | 0.002791 |
| ENSMUSG00000061286 | *Exosc5* | 1463.425 | 4.439185 | 0.002813 |
| ENSMUSG00000021125 | *Arg2* | 29.48143 | 4.758177 | 0.002816 |
| ENSMUSG00000041995 | *Zbed3* | 2850.387 | 3.923182 | 0.002817 |
| ENSMUSG00000081423 | *Gm12882* | 81.47597 | 5.006487 | 0.002821 |
| ENSMUSG00000025487 | *Psmd13* | 3564.112 | 5.229867 | 0.002825 |
| ENSMUSG00000027007 | *Ssfa2* | 2065.607 | 4.465571 | 0.002835 |
| ENSMUSG00000033285 | *Wdr3* | 971.8466 | 4.825191 | 0.002835 |
| ENSMUSG00000027968 | *Larp7* | 1176.358 | 4.485519 | 0.002837 |
| ENSMUSG00000025764 | *Jade1* | 2058.108 | 4.263109 | 0.002848 |
| ENSMUSG00000020219 | *Timm13* | 512.2058 | 4.922933 | 0.002854 |
| ENSMUSG00000051341 | *Zfp52* | 283.7788 | 5.045202 | 0.002857 |
| ENSMUSG00000019947 | *Arid5b* | 716.3831 | 4.361535 | 0.002857 |
| ENSMUSG00000001507 | *Itga3* | 12981.66 | 4.177938 | 0.002865 |
| ENSMUSG00000032180 | *Tmed1* | 1662.241 | 4.15683 | 0.002865 |
| ENSMUSG00000022940 | *Pigp* | 19.31794 | 4.132055 | 0.002866 |
| ENSMUSG00000022999 | *Lmbr1l* | 9086.877 | 5.934354 | 0.002874 |
| ENSMUSG00000048332 | *Lhfp* | 15.12574 | 3.855763 | 0.002877 |
| ENSMUSG00000043671 | *Dpy19l3* | 949.263 | 4.995467 | 0.002878 |
| ENSMUSG00000020256 | *Aldh1l2* | 27.33755 | 4.29158 | 0.002882 |
| ENSMUSG00000034509 | *Mad2l1bp* | 365.0277 | 5.072593 | 0.002896 |
| ENSMUSG00000057193 | *Slc44a2* | 5528.159 | 4.454609 | 0.002897 |
| ENSMUSG00000002205 | *Vrk3* | 1731.306 | 4.988104 | 0.002901 |
| ENSMUSG00000110533 | *LOC665443* | 281.5941 | 5.028908 | 0.002904 |
| ENSMUSG00000078681 | *Tm2d3* | 193.3933 | 5.062675 | 0.002914 |
| ENSMUSG00000069378 | *Prdm6* | 4.896586 | 3.333009 | 0.002921 |
| ENSMUSG00000103367 | *Gm38158* | 12.50179 | 4.032688 | 0.002935 |
| ENSMUSG00000027995 | *Tlr2* | 71.47363 | 5.347445 | 0.00294 |
| ENSMUSG00000059248 | *9-Sep* | 4850.921 | 3.645437 | 0.002943 |
| ENSMUSG00000110841 | *Gpx4* | 449.8191 | 5.736221 | 0.002943 |
| ENSMUSG00000040732 | *Erg* | 274.5001 | 5.020131 | 0.002979 |
| ENSMUSG00000111567 | *Olfr1251* | 61.76058 | 5.100204 | 0.002988 |
| ENSMUSG00000032101 | *Ddx25* | 12.86784 | 4.032785 | 0.002998 |
| ENSMUSG00000025782 | *Taf3* | 5304.667 | 3.925116 | 0.003026 |
| ENSMUSG00000020893 | *Per1* | 5627.697 | 3.92192 | 0.003056 |
| ENSMUSG00000022718 | *Dgcr8* | 4863.767 | 4.413054 | 0.003081 |
| ENSMUSG00000048763 | *Hoxb3* | 142.3402 | 5.101703 | 0.003086 |
| ENSMUSG00000051390 | *Zbtb22* | 1543.915 | 4.191417 | 0.003087 |
| ENSMUSG00000029033 | *Acap3* | 762.1431 | 5.10675 | 0.0031 |
| ENSMUSG00000032285 | *Dnaja4* | 2460.692 | 4.166982 | 0.003102 |
| ENSMUSG00000010406 | *Mrpl52* | 109.2223 | 3.810061 | 0.003108 |
| ENSMUSG00000009741 | *Ubp1* | 4614.79 | 3.503907 | 0.00311 |
| ENSMUSG00000023967 | *Mrps18a* | 856.7452 | 4.356999 | 0.003122 |
| ENSMUSG00000020849 | *Ywhae* | 24091.1 | 2.792841 | 0.003126 |
| ENSMUSG00000024906 | *Mus81* | 911.9421 | 4.71899 | 0.003133 |
| ENSMUSG00000054499 | *Dedd2* | 553.6538 | 5.18063 | 0.003142 |
| ENSMUSG00000041408 | *Wapl* | 4412.698 | 4.234439 | 0.003143 |
| ENSMUSG00000058626 | *Capn11* | 5.722116 | 2.970943 | 0.003145 |
| ENSMUSG00000051375 | *Pcdh1* | 42.0295 | 4.1018 | 0.003146 |
| ENSMUSG00000095567 | *Noc2l* | 22243.68 | 3.786868 | 0.003164 |
| ENSMUSG00000032621 | *Srek1* | 7042.807 | 3.516148 | 0.003165 |
| ENSMUSG00000034871 | *Fam151a* | 36.29594 | 4.688418 | 0.003183 |
| ENSMUSG00000050812 | *AI314180* | 1946.635 | 4.297146 | 0.003189 |
| ENSMUSG00000031355 | *Arhgap6* | 247.2764 | 4.647804 | 0.003204 |
| ENSMUSG00000033871 | *Ppargc1b* | 613.235 | 5.189016 | 0.003246 |
| ENSMUSG00000098557 | *Kctd12* | 2222.124 | 5.523189 | 0.003274 |
| ENSMUSG00000037857 | *Nufip2* | 10430.64 | 3.965746 | 0.003288 |
| ENSMUSG00000018449 | *Rpain* | 40.58476 | 4.187081 | 0.00329 |
| ENSMUSG00000063320 | *1190007I07Rik* | 5.953064 | 3.219966 | 0.003342 |
| ENSMUSG00000028048 | *Gba* | 1334.447 | 4.262079 | 0.003361 |
| ENSMUSG00000050668 | *Gpatch11* | 434.644 | 5.273335 | 0.003365 |
| ENSMUSG00000004460 | *Dnajb11* | 977.046 | 4.113235 | 0.003372 |
| ENSMUSG00000062785 | *Kcnc3* | 8.654498 | 3.622503 | 0.003374 |
| ENSMUSG00000039384 | *Dusp10* | 703.1573 | 5.422174 | 0.003378 |
| ENSMUSG00000039210 | *Gpatch2* | 2683.656 | 4.683308 | 0.003388 |
| ENSMUSG00000028158 | *Mttp* | 57.30102 | 4.803735 | 0.003412 |
| ENSMUSG00000009291 | *Pttg1ip* | 8467.654 | 4.254666 | 0.003415 |
| ENSMUSG00000045518 | *Onecut3* | 189.0931 | 5.249408 | 0.003419 |
| ENSMUSG00000080895 | *Gm11645* | 476.2319 | 5.074796 | 0.003422 |
| ENSMUSG00000019647 | *Sema6a* | 3766.862 | 5.06011 | 0.003435 |
| ENSMUSG00000047844 | *Bex4* | 7288.799 | 5.800463 | 0.00346 |
| ENSMUSG00000030045 | *Mrpl19* | 281.8324 | 5.236098 | 0.003465 |
| ENSMUSG00000052752 | *Traf7* | 3345.242 | 3.876362 | 0.003468 |
| ENSMUSG00000023249 | *Parp3* | 219.0117 | 5.115278 | 0.003474 |
| ENSMUSG00000010307 | *Tmem86a* | 13089.27 | 2.379329 | 0.003481 |
| ENSMUSG00000024091 | *Vapa* | 1772.32 | 3.786198 | 0.003505 |
| ENSMUSG00000035671 | *Zswim4* | 4471.734 | 4.583369 | 0.00353 |
| ENSMUSG00000028330 | *Ncbp1* | 3647.541 | 3.934042 | 0.003563 |
| ENSMUSG00000027933 | *Ints3* | 5277.297 | 3.413071 | 0.003567 |
| ENSMUSG00000084353 | *Gm15452* | 261.1294 | 5.485072 | 0.003591 |
| ENSMUSG00000037369 | *Kdm6a* | 4004.375 | 3.897094 | 0.003604 |
| ENSMUSG00000041354 | *Rgl2* | 1411.459 | 4.186154 | 0.00361 |
| ENSMUSG00000009281 | *Rarres2* | 162.0255 | 4.573738 | 0.003612 |
| ENSMUSG00000048537 | *Phldb1* | 10602.76 | 3.654872 | 0.003628 |
| ENSMUSG00000110576 | *Gm36368* | 187.6974 | 5.298461 | 0.003643 |
| ENSMUSG00000005625 | *Psmd4* | 8644.826 | 2.745371 | 0.003647 |
| ENSMUSG00000024597 | *Slc12a2* | 522.4345 | 3.748217 | 0.003651 |
| ENSMUSG00000024927 | *Rela* | 5165.045 | 3.600084 | 0.003658 |
| ENSMUSG00000015980 | *Lrrc27* | 7.844412 | 3.334033 | 0.00366 |
| ENSMUSG00000022898 | *Dscr3* | 474.9487 | 4.43392 | 0.003667 |
| ENSMUSG00000031751 | *Amfr* | 4498.663 | 2.843572 | 0.003667 |
| ENSMUSG00000014226 | *Cacybp* | 201.8419 | 4.573918 | 0.003691 |
| ENSMUSG00000038619 | *Ensa* | 3527.579 | 4.448549 | 0.003714 |
| ENSMUSG00000025930 | *Msc* | 110.8457 | 5.098805 | 0.003722 |
| ENSMUSG00000021824 | *Ap3m1* | 2403.343 | 4.914091 | 0.003727 |
| ENSMUSG00000022706 | *Mrpl40* | 336.9575 | 4.74009 | 0.003732 |
| ENSMUSG00000030421 | *Uri1* | 709.1026 | 4.302585 | 0.003758 |
| ENSMUSG00000029561 | *Oasl2* | 1327.587 | 3.579825 | 0.003771 |
| ENSMUSG00000030061 | *Uba3* | 1307.128 | 4.281993 | 0.003786 |
| ENSMUSG00000032199 | *Polr2m* | 2154.842 | 4.63091 | 0.00379 |
| ENSMUSG00000018334 | *Ksr1* | 4994.061 | 4.072374 | 0.003793 |
| ENSMUSG00000044807 | *Zfp354c* | 98.55083 | 5.042671 | 0.003799 |
| ENSMUSG00000027472 | *Pdrg1* | 849.7305 | 4.402741 | 0.003802 |
| ENSMUSG00000073725 | *Lmbrd1* | 789.2812 | 4.106554 | 0.003807 |
| ENSMUSG00000095440 | *Fignl2* | 113.2682 | 4.686439 | 0.003818 |
| ENSMUSG00000062275 | *Fbxw24* | 75.88597 | 4.758033 | 0.003849 |
| ENSMUSG00000036790 | *Slitrk2* | 9.209217 | 3.177337 | 0.003871 |
| ENSMUSG00000047139 | *Cd24a* | 180.6477 | 4.751216 | 0.003883 |
| ENSMUSG00000035226 | *Rims4* | 4734.624 | 3.359442 | 0.003888 |
| ENSMUSG00000024937 | *Ehbp1l1* | 1852.323 | 4.557559 | 0.003896 |
| ENSMUSG00000026889 | *Rbm18* | 3690.425 | 4.604596 | 0.003901 |
| ENSMUSG00000083798 | *Gm14584* | 81.00174 | 5.367515 | 0.003904 |
| ENSMUSG00000039615 | *Stub1* | 4809.857 | 4.660653 | 0.003907 |
| ENSMUSG00000002007 | *Srpk3* | 24.09927 | 4.177539 | 0.003947 |
| ENSMUSG00000108302 | *Gm18207* | 17.62711 | 3.801082 | 0.003953 |
| ENSMUSG00000022364 | *Tbc1d31* | 1335.844 | 4.295549 | 0.003955 |
| ENSMUSG00000019122 | *Ccl9* | 50.78334 | 3.944131 | 0.003979 |
| ENSMUSG00000021377 | *Dek* | 6630.055 | 3.951816 | 0.00398 |
| ENSMUSG00000020521 | *Rnft1* | 37.5076 | 3.907407 | 0.003983 |
| ENSMUSG00000090698 | *Apold1* | 7.430206 | 3.677578 | 0.003989 |
| ENSMUSG00000036026 | *Tmem63b* | 4964.442 | 3.518996 | 0.004003 |
| ENSMUSG00000022972 | *1110004E09Rik* | 2081.089 | 4.866003 | 0.004014 |
| ENSMUSG00000003410 | *Elavl3* | 24.72359 | 4.064573 | 0.004016 |
| ENSMUSG00000046079 | *Lrrc8d* | 3995.757 | 3.818254 | 0.004034 |
| ENSMUSG00000028036 | *Ptgfr* | 744.8026 | 5.441063 | 0.004046 |
| ENSMUSG00000100351 | *Gm7867* | 6.536213 | 3.238954 | 0.004051 |
| ENSMUSG00000004846 | *Plod3* | 12151.25 | 3.774946 | 0.004054 |
| ENSMUSG00000047371 | *Zfp768* | 1481.199 | 5.075976 | 0.004075 |
| ENSMUSG00000031119 | *Gpc4* | 12022.53 | 2.631068 | 0.004094 |
| ENSMUSG00000020029 | *Nudt4* | 5220.449 | 3.876776 | 0.004108 |
| ENSMUSG00000032827 | *Ppp1r9a* | 9749.747 | 4.260145 | 0.004128 |
| ENSMUSG00000029196 | *Tada2b* | 2048.96 | 4.251699 | 0.004142 |
| ENSMUSG00000025192 | *Entpd7* | 3500.565 | 3.917089 | 0.004152 |
| ENSMUSG00000028931 | *Kcnab2* | 14.30918 | 3.730699 | 0.004164 |
| ENSMUSG00000002297 | *Dbf4* | 160.377 | 4.50041 | 0.004165 |
| ENSMUSG00000010755 | *Cars* | 2159.175 | 4.088906 | 0.004171 |
| ENSMUSG00000046792 | *Zfp787* | 2171.578 | 4.633769 | 0.00421 |
| ENSMUSG00000032497 | *Lrrfip2* | 2367.084 | 4.121111 | 0.004211 |
| ENSMUSG00000002699 | *Lcp2* | 390.8815 | 4.411287 | 0.004211 |
| ENSMUSG00000030930 | *Chst15* | 7143.687 | 3.724663 | 0.00423 |
| ENSMUSG00000020277 | *Pfkl* | 7577.22 | 4.478795 | 0.004238 |
| ENSMUSG00000025407 | *Gli1* | 76.8056 | 4.310917 | 0.004244 |
| ENSMUSG00000047507 | *Baiap3* | 7.170146 | 3.200889 | 0.004247 |
| ENSMUSG00000114942 | *Lats2* | 306.094 | 5.265044 | 0.004284 |
| ENSMUSG00000031864 | *Ints10* | 4804.152 | 4.406859 | 0.004301 |
| ENSMUSG00000022339 | *Ebag9* | 447.3474 | 5.089528 | 0.004308 |
| ENSMUSG00000027203 | *Dut* | 314.2541 | 4.879502 | 0.004318 |
| ENSMUSG00000039501 | *Znfx1* | 4016.944 | 3.448218 | 0.004324 |
| ENSMUSG00000023279 | *Bmp15* | 254.784 | 5.350092 | 0.004348 |
| ENSMUSG00000020921 | *Tmem101* | 1801.827 | 4.92752 | 0.004371 |
| ENSMUSG00000023348 | *Trip6* | 2233.56 | 3.805295 | 0.004387 |
| ENSMUSG00000049686 | *Orai1* | 1149.7 | 4.570107 | 0.004388 |
| ENSMUSG00000030095 | *Tmem43* | 1232.536 | 2.896596 | 0.004389 |
| ENSMUSG00000031862 | *Atp13a1* | 5211.745 | 4.380755 | 0.004403 |
| ENSMUSG00000015165 | *Hnrnpl* | 8331.884 | 3.653907 | 0.004415 |
| ENSMUSG00000021012 | *Zc3h14* | 2535.594 | 4.295407 | 0.00442 |
| ENSMUSG00000029790 | *Cep41* | 10.9655 | 3.41392 | 0.004436 |
| ENSMUSG00000017837 | *Nkiras2* | 709.9367 | 4.490285 | 0.004445 |
| ENSMUSG00000054008 | *Ndst1* | 5948.328 | 3.546852 | 0.004468 |
| ENSMUSG00000028115 | *Bnipl* | 7.190492 | 3.341227 | 0.004468 |
| ENSMUSG00000059288 | *Cdyl* | 746.3824 | 5.000091 | 0.004479 |
| ENSMUSG00000042489 | *Clspn* | 4953.524 | 4.653939 | 0.00449 |
| ENSMUSG00000056708 | *Ier5* | 685.427 | 5.155462 | 0.004508 |
| ENSMUSG00000003929 | *Zfp81* | 34.58408 | 3.5705 | 0.004534 |
| ENSMUSG00000037007 | *Zfp113* | 5850.491 | 4.648113 | 0.004545 |
| ENSMUSG00000032035 | *Ets1* | 294.1359 | 4.737199 | 0.004548 |
| ENSMUSG00000035495 | *Tstd2* | 1431.359 | 4.881656 | 0.004563 |
| ENSMUSG00000040596 | *Pogk* | 3453.589 | 4.249737 | 0.004621 |
| ENSMUSG00000031799 | *Tpm4* | 6217.975 | 3.09317 | 0.00464 |
| ENSMUSG00000028282 | *Casp8ap2* | 568.1574 | 4.871359 | 0.004655 |
| ENSMUSG00000015745 | *Plekho1* | 351.8545 | 4.48838 | 0.004698 |
| ENSMUSG00000039001 | *Rps21* | 2600.659 | 4.762715 | 0.00471 |
| ENSMUSG00000041592 | *Sdk2* | 89.31992 | 4.49746 | 0.004711 |
| ENSMUSG00000019943 | *Atp2b1* | 6607.545 | 3.229693 | 0.004716 |
| ENSMUSG00000003810 | *Mast2* | 2151.445 | 3.640354 | 0.00473 |
| ENSMUSG00000055093 | *Gm8430* | 774.3146 | 4.904821 | 0.004736 |
| ENSMUSG00000047514 | *Tspyl1* | 2096.003 | 4.290527 | 0.004739 |
| ENSMUSG00000042417 | *Ccno* | 133.1339 | 4.701871 | 0.004741 |
| ENSMUSG00000025980 | *Hspd1* | 3425.554 | 4.239059 | 0.004758 |
| ENSMUSG00000046191 | *Pcdhb20* | 16.91066 | 3.548315 | 0.004764 |
| ENSMUSG00000038612 | *Mcl1* | 1196.836 | 3.457058 | 0.004765 |
| ENSMUSG00000036858 | *Ptcra* | 346.1655 | 4.176057 | 0.004773 |
| ENSMUSG00000050148 | *Ubqln2* | 2927.15 | 4.057825 | 0.004776 |
| ENSMUSG00000001435 | *Col18a1* | 2107.243 | 4.301439 | 0.004784 |
| ENSMUSG00000047414 | *Flrt2* | 180.3888 | 4.090544 | 0.004793 |
| ENSMUSG00000035247 | *Hectd1* | 10766.05 | 2.986597 | 0.00483 |
| ENSMUSG00000021072 | *Tmx1* | 2393.462 | 3.676791 | 0.004849 |
| ENSMUSG00000059119 | *Nap1l4* | 7310.492 | 4.003099 | 0.004851 |
| ENSMUSG00000114931 | *AC135377* | 13.35365 | 4.313013 | 0.004879 |
| ENSMUSG00000027329 | *Spef1* | 1099.183 | 4.66686 | 0.004883 |
| ENSMUSG00000021557 | *Agtpbp1* | 613.075 | 4.408856 | 0.004889 |
| ENSMUSG00000104777 | *Gm4865* | 9.018912 | 3.687216 | 0.004889 |
| ENSMUSG00000031161 | *Hdac6* | 1836.993 | 4.334152 | 0.004907 |
| ENSMUSG00000052407 | *Ccdc171* | 364.2782 | 3.985733 | 0.004908 |
| ENSMUSG00000004264 | *Phb2* | 6872.672 | 3.936758 | 0.004941 |
| ENSMUSG00000061013 | *Mkx* | 1236.06 | 3.665316 | 0.004941 |
| ENSMUSG00000038274 | *Fau* | 537.2343 | 4.449433 | 0.004944 |
| ENSMUSG00000035049 | *Rrp12* | 4695.848 | 4.449108 | 0.004971 |
| ENSMUSG00000040952 | *Rps19* | 3596.946 | 3.427987 | 0.004993 |
| ENSMUSG00000019960 | *Dusp6* | 2759.078 | 4.93328 | 0.00503 |
| ENSMUSG00000022529 | *Zfp263* | 583.756 | 4.460675 | 0.005032 |
| ENSMUSG00000021287 | *Xrcc3* | 1244.76 | 4.331848 | 0.005046 |
| ENSMUSG00000043257 | *Pigv* | 835.3484 | 4.580439 | 0.005046 |
| ENSMUSG00000030499 | *Kctd15* | 1090.307 | 5.311749 | 0.005056 |
| ENSMUSG00000067851 | *Arfgef1* | 2407.773 | 4.014652 | 0.005084 |
| ENSMUSG00000037149 | *Ddx1* | 1126.875 | 4.085188 | 0.00511 |
| ENSMUSG00000025066 | *Sfr1* | 1639.954 | 4.272673 | 0.005114 |
| ENSMUSG00000031226 | *Pbdc1* | 913.7312 | 4.279111 | 0.005145 |
| ENSMUSG00000048170 | *Mcmbp* | 2093.189 | 4.051303 | 0.005159 |
| ENSMUSG00000030409 | *Dmpk* | 1654.378 | 3.654525 | 0.005174 |
| ENSMUSG00000071235 | *Vrtn* | 177.3416 | 4.090237 | 0.005181 |
| ENSMUSG00000063870 | *Chd4* | 102552.9 | 3.046537 | 0.005183 |
| ENSMUSG00000061374 | *Fiz1* | 1526.71 | 3.682533 | 0.005195 |
| ENSMUSG00000007036 | *Abhd16a* | 3954.429 | 4.910599 | 0.005204 |
| ENSMUSG00000033152 | *Podxl2* | 3093.019 | 4.385404 | 0.005205 |
| ENSMUSG00000024883 | *Rin1* | 510.4969 | 4.973672 | 0.005206 |
| ENSMUSG00000038990 | *Cables2* | 848.2575 | 4.479671 | 0.005218 |
| ENSMUSG00000052296 | *Ppp6r1* | 7487.546 | 3.275933 | 0.005227 |
| ENSMUSG00000061758 | *Akr1b10* | 76.15569 | 4.469922 | 0.005237 |
| ENSMUSG00000031353 | *Rbbp7* | 5788.948 | 3.951529 | 0.005248 |
| ENSMUSG00000007038 | *Neu1* | 1890.278 | 4.444598 | 0.00525 |
| ENSMUSG00000083012 | *Fam220a* | 2106.495 | 4.860605 | 0.005256 |
| ENSMUSG00000031983 | *2310022B05Rik* | 1052.555 | 4.12968 | 0.005269 |
| ENSMUSG00000032482 | *Cspg5* | 22.56956 | 3.813877 | 0.005269 |
| ENSMUSG00000039004 | *Bmp6* | 1632.138 | 5.146486 | 0.005271 |
| ENSMUSG00000004085 | *Map3k20* | 2060.503 | 3.94534 | 0.005281 |
| ENSMUSG00000050708 | *Ftl1* | 20221.45 | 2.252928 | 0.005288 |
| ENSMUSG00000085006 | *BC021767* | 32.94598 | 4.252998 | 0.005292 |
| ENSMUSG00000052406 | *Rexo4* | 1547.757 | 4.798095 | 0.005299 |
| ENSMUSG00000038729 | *Akap2* | 11229.57 | 4.118967 | 0.00532 |
| ENSMUSG00000026064 | *Ptp4a1* | 609.9013 | 4.196149 | 0.005323 |
| ENSMUSG00000028771 | *Ptpn12* | 1758.978 | 4.783004 | 0.005333 |
| ENSMUSG00000048191 | *Muc6* | 9643.883 | 5.770761 | 0.005344 |
| ENSMUSG00000037544 | *Dlgap5* | 582.4231 | 4.800447 | 0.005369 |
| ENSMUSG00000036098 | *Myrf* | 1119.197 | 4.050596 | 0.005376 |
| ENSMUSG00000003378 | *Grik5* | 7645.739 | 3.708403 | 0.005387 |
| ENSMUSG00000005514 | *Por* | 5169.874 | 3.300835 | 0.005407 |
| ENSMUSG00000028042 | *Zbtb7b* | 1406.344 | 4.024312 | 0.005447 |
| ENSMUSG00000032966 | *Fkbp1a* | 5510.318 | 3.266594 | 0.005463 |
| ENSMUSG00000025127 | *Gcgr* | 58.58559 | 4.583292 | 0.005465 |
| ENSMUSG00000053062 | *Jam2* | 4717.001 | 4.088516 | 0.005468 |
| ENSMUSG00000027534 | *Snx16* | 11.73778 | 3.269517 | 0.005471 |
| ENSMUSG00000002477 | *Snrpd1* | 1088.153 | 3.671327 | 0.005481 |
| ENSMUSG00000025997 | *Ikzf2* | 145.4674 | 4.583547 | 0.005487 |
| ENSMUSG00000076441 | *Ass1* | 360.7852 | 4.529931 | 0.005507 |
| ENSMUSG00000016344 | *Ppdpf* | 270.0052 | 4.863193 | 0.005534 |
| ENSMUSG00000004040 | *Stat3* | 2977.262 | 3.567759 | 0.005535 |
| ENSMUSG00000022229 | *Atp12a* | 5.995959 | 3.338824 | 0.005573 |
| ENSMUSG00000031578 | *Mak16* | 3548.228 | 4.793388 | 0.005589 |
| ENSMUSG00000051590 | *Map3k19* | 18.8565 | 3.842545 | 0.005608 |
| ENSMUSG00000042541 | *Sem1* | 449.5458 | 4.316578 | 0.005626 |
| ENSMUSG00000096458 | *Moap1* | 69.99771 | 4.411653 | 0.005634 |
| ENSMUSG00000066362 | *Rps13* | 25.51528 | 3.664854 | 0.005647 |
| ENSMUSG00000003070 | *Efna2* | 30.60629 | 4.219658 | 0.005664 |
| ENSMUSG00000050751 | *Pgbd5* | 2058.472 | 4.660906 | 0.005682 |
| ENSMUSG00000016626 | *Nlrp14* | 267.2279 | 5.275988 | 0.005683 |
| ENSMUSG00000029723 | *Tsc22d4* | 3234.037 | 3.276972 | 0.005687 |
| ENSMUSG00000022487 | *Gtsf1* | 5.263372 | 2.988725 | 0.005693 |
| ENSMUSG00000035770 | *Dync1li2* | 4530.05 | 3.930495 | 0.005704 |
| ENSMUSG00000022144 | *Gdnf* | 1745.557 | 4.66662 | 0.00572 |
| ENSMUSG00000034220 | *Gpc1* | 7346.463 | 3.757363 | 0.005743 |
| ENSMUSG00000052151 | *Plpp2* | 1661.776 | 4.747234 | 0.005745 |
| ENSMUSG00000026974 | *Zmynd19* | 424.244 | 4.146543 | 0.005746 |
| ENSMUSG00000004948 | *Zp3* | 1866.332 | 4.914271 | 0.005773 |
| ENSMUSG00000028776 | *Tinagl1* | 4062.863 | 4.340246 | 0.005787 |
| ENSMUSG00000101188 | *Eif4a* | 35013.45 | 2.860399 | 0.005811 |
| ENSMUSG00000021866 | *Anxa11* | 837.9411 | 3.593779 | 0.00582 |
| ENSMUSG00000027669 | *Gnb4* | 1095.67 | 4.482789 | 0.005824 |
| ENSMUSG00000106038 | *Gm4962* | 1242.439 | 4.442808 | 0.00586 |
| ENSMUSG00000070802 | *Pnmal2* | 546.4646 | 4.67785 | 0.005892 |
| ENSMUSG00000074746 | *Pdzd8* | 8816.652 | 3.391961 | 0.005898 |
| ENSMUSG00000050966 | *Lin28a* | 34.22069 | 4.161177 | 0.005906 |
| ENSMUSG00000020522 | *Mfap3* | 1853.753 | 3.994378 | 0.005918 |
| ENSMUSG00000041959 | *S100a10* | 1241.738 | 4.148896 | 0.005931 |
| ENSMUSG00000003585 | *Sec14l2* | 108.0347 | 4.589772 | 0.005944 |
| ENSMUSG00000073409 | *H2* | 64.16259 | 4.746819 | 0.005944 |
| ENSMUSG00000032374 | *Plod2* | 14167.35 | 3.663472 | 0.005946 |
| ENSMUSG00000051256 | *Jagn1* | 1150.956 | 5.112012 | 0.00598 |
| ENSMUSG00000031990 | *Jam3* | 591.4672 | 4.732736 | 0.006017 |
| ENSMUSG00000074030 | *Exoc8* | 1772.712 | 4.805334 | 0.006032 |
| ENSMUSG00000066595 | *Flvcr1* | 234.4425 | 4.425477 | 0.006064 |
| ENSMUSG00000082768 | *Gm12727* | 9.943455 | 3.524449 | 0.006072 |
| ENSMUSG00000032405 | *Pias1* | 1592.704 | 4.332687 | 0.006077 |
| ENSMUSG00000079235 | *Ccdc13* | 5.30498 | 2.949972 | 0.006092 |
| ENSMUSG00000018858 | *Mrpl58* | 380.5644 | 4.307974 | 0.006095 |
| ENSMUSG00000028617 | *Lrrc42* | 499.0093 | 3.914619 | 0.006097 |
| ENSMUSG00000096370 | *Gm21992* | 4772.095 | 4.420501 | 0.006108 |
| ENSMUSG00000028093 | *Acp6* | 1876.888 | 4.647794 | 0.006128 |
| ENSMUSG00000028896 | *Rcc1* | 1901.384 | 3.990584 | 0.006137 |
| ENSMUSG00000083579 | *Gm15538* | 406.8672 | 5.378146 | 0.006157 |
| ENSMUSG00000079225 | *Gm9531* | 134707.8 | 5.837338 | 0.006158 |
| ENSMUSG00000040822 | *1700123O20Rik* | 149.4994 | 4.61928 | 0.006165 |
| ENSMUSG00000030609 | *Aen* | 1186.705 | 3.824215 | 0.006171 |
| ENSMUSG00000041235 | *Chd7* | 1241.979 | 4.108892 | 0.006171 |
| ENSMUSG00000042801 | *Olfr769* | 13.47332 | 3.288189 | 0.00619 |
| ENSMUSG00000074569 | *Gcnt7* | 438.0441 | 3.875514 | 0.006193 |
| ENSMUSG00000113871 | *AC134439* | 7.598842 | 3.14059 | 0.006195 |
| ENSMUSG00000004843 | *Chmp2b* | 221.8581 | 4.865613 | 0.006229 |
| ENSMUSG00000047123 | *Ticam1* | 874.1182 | 4.439672 | 0.006258 |
| ENSMUSG00000030888 | *Rrp8* | 180.7223 | 4.634003 | 0.006277 |
| ENSMUSG00000069769 | *Msi2* | 8226.195 | 2.528129 | 0.006282 |
| ENSMUSG00000000903 | *Vpreb3* | 28.62716 | 4.114718 | 0.006334 |
| ENSMUSG00000015839 | *Nfe2l2* | 1213.874 | 3.481149 | 0.006365 |
| ENSMUSG00000042613 | *Pbxip1* | 3914.801 | 4.104798 | 0.006414 |
| ENSMUSG00000050064 | *Zfp697* | 1769.489 | 4.84098 | 0.00642 |
| ENSMUSG00000020211 | *Sf3a2* | 1586.435 | 3.521999 | 0.006428 |
| ENSMUSG00000026248 | *Mrpl44* | 77.93302 | 4.0317 | 0.006438 |
| ENSMUSG00000036834 | *Plch1* | 92.68411 | 4.231651 | 0.006449 |
| ENSMUSG00000093904 | *Tomm20* | 1628.925 | 3.952531 | 0.006459 |
| ENSMUSG00000030847 | *Bag3* | 1020.428 | 3.554924 | 0.006493 |
| ENSMUSG00000015568 | *Lpl* | 3624.17 | 4.631019 | 0.006494 |
| ENSMUSG00000022594 | *Lynx1* | 628.7552 | 5.588118 | 0.006498 |
| ENSMUSG00000037095 | *Lrg1* | 68.63069 | 4.283147 | 0.006505 |
| ENSMUSG00000005481 | *Ddx39* | 2991.992 | 3.58246 | 0.006518 |
| ENSMUSG00000023966 | *Rsph9* | 11.27679 | 3.677919 | 0.006525 |
| ENSMUSG00000023110 | *Prmt5* | 3989.781 | 4.474793 | 0.00653 |
| ENSMUSG00000035164 | *Zc3h12c* | 3839.537 | 5.388428 | 0.00654 |
| ENSMUSG00000038555 | *Reep2* | 281.9706 | 4.552735 | 0.006545 |
| ENSMUSG00000063884 | *Ptcd3* | 1022.066 | 4.271108 | 0.006548 |
| ENSMUSG00000047731 | *Wbp1l* | 8952.178 | 2.996857 | 0.006615 |
| ENSMUSG00000029364 | *Wsb2* | 7415.491 | 3.67328 | 0.006619 |
| ENSMUSG00000040616 | *Tmem51* | 866.6185 | 4.408145 | 0.006639 |
| ENSMUSG00000021996 | *Esd* | 444.4937 | 4.643443 | 0.006647 |
| ENSMUSG00000029093 | *Sorcs2* | 100.7958 | 3.915615 | 0.006711 |
| ENSMUSG00000014609 | *Chrne* | 4.970032 | 2.902719 | 0.006717 |
| ENSMUSG00000089662 | *Gm14057* | 31.86787 | 3.425849 | 0.00672 |
| ENSMUSG00000092035 | *Peg10* | 11907.55 | 3.579447 | 0.006776 |
| ENSMUSG00000018861 | *Fdxr* | 7715.809 | 3.762791 | 0.006778 |
| ENSMUSG00000015697 | *Setdb1* | 2708.851 | 3.784068 | 0.006802 |
| ENSMUSG00000092454 | *Gm2991* | 5.326141 | 2.902493 | 0.00681 |
| ENSMUSG00000008668 | *Rps18* | 30938.76 | 3.659927 | 0.006821 |
| ENSMUSG00000031662 | *Snx20* | 32.66531 | 3.759072 | 0.006858 |
| ENSMUSG00000032014 | *Oaf* | 2833.704 | 3.002052 | 0.006877 |
| ENSMUSG00000032344 | *Mb21d1* | 5.0447 | 2.536222 | 0.006887 |
| ENSMUSG00000092541 | *Gm20537* | 116.1829 | 4.482395 | 0.006897 |
| ENSMUSG00000029461 | *Fam168a* | 5092.518 | 4.318025 | 0.006924 |
| ENSMUSG00000070803 | *Cited4* | 3816.322 | 3.744614 | 0.006957 |
| ENSMUSG00000024817 | *Uhrf2* | 1277.193 | 4.102876 | 0.006967 |
| ENSMUSG00000020902 | *Ntn1* | 107.1758 | 4.403524 | 0.006989 |
| ENSMUSG00000022241 | *Tars* | 4084.76 | 3.794395 | 0.00704 |
| ENSMUSG00000020473 | *Aebp1* | 12240.74 | 3.142065 | 0.007052 |
| ENSMUSG00000025962 | *Fastkd2* | 677.9183 | 5.210955 | 0.007073 |
| ENSMUSG00000020657 | *Dnajc27* | 456.7807 | 5.248696 | 0.007095 |
| ENSMUSG00000008475 | *Arpc5* | 3063.918 | 4.154492 | 0.007109 |
| ENSMUSG00000025959 | *Klf7* | 1751.48 | 3.876849 | 0.007134 |
| ENSMUSG00000040345 | *Arhgap9* | 5.306453 | 3.097015 | 0.007143 |
| ENSMUSG00000069662 | *Marcks* | 1676.768 | 2.975981 | 0.007188 |
| ENSMUSG00000052926 | *Rnaseh2a* | 1140.866 | 3.728081 | 0.007188 |
| ENSMUSG00000040028 | *Elavl1* | 2333.512 | 4.179534 | 0.007188 |
| ENSMUSG00000055799 | *Tcf7l1* | 3823.868 | 4.68918 | 0.007204 |
| ENSMUSG00000023068 | *Nus1* | 772.5794 | 4.180515 | 0.007243 |
| ENSMUSG00000026499 | *Acbd3* | 4847.798 | 3.653593 | 0.007249 |
| ENSMUSG00000022403 | *St13* | 2604.354 | 3.336131 | 0.007261 |
| ENSMUSG00000110679 | *Rpl10* | 887.7591 | 6.741851 | 0.007273 |
| ENSMUSG00000021109 | *Hif1a* | 4456.867 | 3.503569 | 0.007277 |
| ENSMUSG00000072974 | *Gm4787* | 17.55388 | 2.991163 | 0.00729 |
| ENSMUSG00000005609 | *Ctr9* | 14680.02 | 4.07744 | 0.007296 |
| ENSMUSG00000028886 | *Eya3* | 1823.274 | 3.873348 | 0.007299 |
| ENSMUSG00000006542 | *Prkag3* | 2521.403 | 5.915064 | 0.007301 |
| ENSMUSG00000025534 | *Gusb* | 6561.201 | 5.676702 | 0.007303 |
| ENSMUSG00000071533 | *Pcnp* | 1981.451 | 4.793924 | 0.007317 |
| ENSMUSG00000096557 | *Gm7258* | 6.236417 | 2.934759 | 0.00737 |
| ENSMUSG00000024812 | *Tjp2* | 1199.81 | 4.429343 | 0.007392 |
| ENSMUSG00000024926 | *Kat5* | 707.4576 | 4.347752 | 0.007431 |
| ENSMUSG00000004892 | *Bcan* | 35.49913 | 3.599904 | 0.007436 |
| ENSMUSG00000034111 | *Tmed8* | 1644.895 | 4.745217 | 0.007469 |
| ENSMUSG00000056201 | *Cfl1* | 14243.86 | 2.358559 | 0.007479 |
| ENSMUSG00000068882 | *Ssb* | 5194.084 | 3.987052 | 0.007486 |
| ENSMUSG00000022214 | *Dcaf11* | 2870.582 | 3.61575 | 0.007495 |
| ENSMUSG00000034773 | *BC030867* | 85.06511 | 3.513278 | 0.007504 |
| ENSMUSG00000009112 | *Bcl2l13* | 1275.392 | 3.268893 | 0.007539 |
| ENSMUSG00000029767 | *Calu* | 2756.794 | 3.073811 | 0.007576 |
| ENSMUSG00000021635 | *Rad17* | 139.4999 | 4.040391 | 0.007586 |
| ENSMUSG00000011096 | *Akt1s1* | 1579.571 | 3.541354 | 0.007614 |
| ENSMUSG00000024767 | *Otub1* | 8981.836 | 4.030432 | 0.007631 |
| ENSMUSG00000025190 | *Got1* | 2604.512 | 3.490766 | 0.007632 |
| ENSMUSG00000053289 | *Ddx10* | 894.9493 | 3.805784 | 0.007648 |
| ENSMUSG00000032350 | *Gclc* | 3432.624 | 3.673897 | 0.007651 |
| ENSMUSG00000025351 | *Cd63* | 3041.893 | 2.315424 | 0.007663 |
| ENSMUSG00000032323 | *Cyp11a1* | 38418.93 | 2.221145 | 0.007739 |
| ENSMUSG00000014782 | *Plekhg4* | 49.67152 | 4.067554 | 0.007753 |
| ENSMUSG00000027130 | *Slc12a6* | 8859.764 | 4.936906 | 0.007768 |
| ENSMUSG00000063065 | *Mapk3* | 1844.362 | 3.804094 | 0.007824 |
| ENSMUSG00000020585 | *Laptm4a* | 4921.584 | 4.004632 | 0.007826 |
| ENSMUSG00000037266 | *Rsrp1* | 3808.695 | 3.072056 | 0.007878 |
| ENSMUSG00000023286 | *Ube2j2* | 576.8305 | 4.16859 | 0.007881 |
| ENSMUSG00000057093 | *Zfp607b* | 19.45871 | 3.50639 | 0.007889 |
| ENSMUSG00000038178 | *Slc43a2* | 2333.264 | 4.074538 | 0.007895 |
| ENSMUSG00000054161 | *Fam83e* | 115.1692 | 4.209163 | 0.00791 |
| ENSMUSG00000002489 | *Tiam1* | 2015.939 | 3.794391 | 0.007918 |
| ENSMUSG00000043614 | *Vps37d* | 11.58755 | 3.139795 | 0.007977 |
| ENSMUSG00000020170 | *Frs2* | 5074.548 | 3.539546 | 0.007995 |
| ENSMUSG00000108815 | *AC149222* | 2438.933 | 6.980348 | 0.007995 |
| ENSMUSG00000039330 | *Tsga10ip* | 4.805033 | 2.811106 | 0.008046 |
| ENSMUSG00000019054 | *Fis1* | 1888.559 | 4.652332 | 0.00807 |
| ENSMUSG00000098016 | *Gm9211* | 4.93896 | 2.60925 | 0.008121 |
| ENSMUSG00000037331 | *Larp1* | 13778.48 | 2.629846 | 0.008138 |
| ENSMUSG00000010045 | *Tmem115* | 1758.729 | 3.953158 | 0.008142 |
| ENSMUSG00000048899 | *Rimkla* | 46.74291 | 3.99613 | 0.008194 |
| ENSMUSG00000025613 | *Cct8* | 3031.832 | 2.875606 | 0.008205 |
| ENSMUSG00000001131 | *Timp1* | 78.52652 | 3.69233 | 0.008219 |
| ENSMUSG00000041020 | *Map7d2* | 9.272542 | 3.042034 | 0.008236 |
| ENSMUSG00000047388 | *Atmin* | 727.2939 | 4.428845 | 0.008246 |
| ENSMUSG00000032623 | *Oas1d* | 358.6965 | 4.162934 | 0.008269 |
| ENSMUSG00000034543 | *Morc2a* | 5083.6 | 3.447131 | 0.008275 |
| ENSMUSG00000027782 | *Kpna4* | 1140.964 | 4.354245 | 0.008301 |
| ENSMUSG00000022443 | *Myh9* | 32194.8 | 3.038048 | 0.008331 |
| ENSMUSG00000037001 | *Zfp39* | 303.9685 | 3.907784 | 0.008388 |
| ENSMUSG00000027255 | *Arfgap2* | 6382.065 | 3.764158 | 0.008408 |
| ENSMUSG00000087370 | *Tmem170b* | 456.6961 | 3.739312 | 0.00843 |
| ENSMUSG00000024921 | *Smarca2* | 10089.72 | 4.047266 | 0.008432 |
| ENSMUSG00000046139 | *Patl1* | 3524.619 | 3.563753 | 0.00844 |
| ENSMUSG00000054206 | *Gzmm* | 5.853601 | 2.791065 | 0.008448 |
| ENSMUSG00000042354 | *Gnl3* | 1193.939 | 3.965662 | 0.008462 |
| ENSMUSG00000018363 | *Smurf2* | 2829.147 | 4.867487 | 0.00847 |
| ENSMUSG00000037326 | *Capn15* | 3720.85 | 3.727961 | 0.008523 |
| ENSMUSG00000060882 | *Kcnd2* | 264.1501 | 4.197563 | 0.00857 |
| ENSMUSG00000061111 | *Mcrip1* | 2107.476 | 3.715067 | 0.008576 |
| ENSMUSG00000079215 | *Zfp664* | 5876.298 | 4.180746 | 0.008583 |
| ENSMUSG00000063904 | *Dpp3* | 2110.552 | 3.10572 | 0.008595 |
| ENSMUSG00000060924 | *Csmd1* | 5016.902 | 3.720829 | 0.008607 |
| ENSMUSG00000084842 | *Pabpc1l2b* | 52.51399 | 4.250433 | 0.008611 |
| ENSMUSG00000096576 | *Oog1* | 4.878373 | 2.779099 | 0.008622 |
| ENSMUSG00000080969 | *Gm12372* | 6.400905 | 3.09364 | 0.008623 |
| ENSMUSG00000023156 | *Rpp14* | 147.3699 | 4.325667 | 0.008648 |
| ENSMUSG00000037613 | *Tnfrsf23* | 17.56985 | 3.761494 | 0.008673 |
| ENSMUSG00000019864 | *Rtn4ip1* | 158.7984 | 4.67715 | 0.008681 |
| ENSMUSG00000040731 | *Eif4h* | 11076.36 | 1.972684 | 0.008713 |
| ENSMUSG00000003072 | *Atp5d* | 2028.531 | 3.638303 | 0.008713 |
| ENSMUSG00000028478 | *Clta* | 4071.619 | 2.887242 | 0.008749 |
| ENSMUSG00000010110 | *Stx5a* | 1895.539 | 3.535655 | 0.008755 |
| ENSMUSG00000066900 | *Suds3* | 4960.118 | 3.731387 | 0.008845 |
| ENSMUSG00000028273 | *Pdlim5* | 1293.564 | 4.327732 | 0.008862 |
| ENSMUSG00000039682 | *Lap3* | 1249.429 | 4.790272 | 0.008865 |
| ENSMUSG00000078716 | *Tmem8b* | 2520.693 | 3.658243 | 0.008875 |
| ENSMUSG00000024991 | *Eif3a* | 26786.33 | 2.357971 | 0.008931 |
| ENSMUSG00000020121 | *Srgap1* | 1464.068 | 4.772735 | 0.008953 |
| ENSMUSG00000025885 | *Myo5b* | 236.935 | 5.295974 | 0.008956 |
| ENSMUSG00000028195 | *Cyr61* | 1581.307 | 4.335106 | 0.00896 |
| ENSMUSG00000058230 | *Arhgap35* | 8560.295 | 3.252038 | 0.008989 |
| ENSMUSG00000019590 | *Cyb561* | 253.4804 | 4.001684 | 0.009015 |
| ENSMUSG00000021823 | *Vcl* | 9427.212 | 2.803799 | 0.009022 |
| ENSMUSG00000024381 | *Bin1* | 5666.313 | 3.738324 | 0.009045 |
| ENSMUSG00000030602 | *Pak4* | 9354.059 | 4.073559 | 0.009123 |
| ENSMUSG00000049504 | *Proser1* | 4776.394 | 3.48866 | 0.009137 |
| ENSMUSG00000020923 | *Ubtf* | 52747.37 | 3.345346 | 0.009138 |
| ENSMUSG00000021906 | *Oxnad1* | 324.2684 | 4.609769 | 0.009187 |
| ENSMUSG00000025337 | *Sbds* | 4483.038 | 4.165834 | 0.009204 |
| ENSMUSG00000041528 | *Rnf123* | 4489.743 | 3.660984 | 0.009209 |
| ENSMUSG00000020091 | *Eif4ebp2* | 6151.52 | 3.280531 | 0.009211 |
| ENSMUSG00000102478 | *BC085271* | 111.2084 | 4.196326 | 0.009237 |
| ENSMUSG00000063919 | *Srrm4* | 5.341433 | 2.842529 | 0.009249 |
| ENSMUSG00000019842 | *Traf3ip2* | 1162.918 | 5.198981 | 0.009255 |
| ENSMUSG00000053819 | *Camk2d* | 3030.769 | 4.158882 | 0.009256 |
| ENSMUSG00000023345 | *Poc1a* | 795.3377 | 4.254981 | 0.009259 |
| ENSMUSG00000025324 | *Atp10a* | 6068.12 | 3.131433 | 0.009269 |
| ENSMUSG00000041695 | *Kcnj2* | 49.86908 | 3.905958 | 0.009282 |
| ENSMUSG00000027712 | *Anxa5* | 4972.046 | 3.808281 | 0.009349 |
| ENSMUSG00000047669 | *Msl3l2* | 77.44119 | 3.836437 | 0.009368 |
| ENSMUSG00000041459 | *Tardbp* | 3592.732 | 2.268891 | 0.009369 |
| ENSMUSG00000017144 | *Rnd3* | 1495.118 | 4.600746 | 0.00942 |
| ENSMUSG00000018008 | *Cyth4* | 50.72981 | 4.050651 | 0.009436 |
| ENSMUSG00000012819 | *Cdh23* | 35.97271 | 3.397007 | 0.009455 |
| ENSMUSG00000057963 | *Itpk1* | 2885.931 | 3.984059 | 0.00956 |
| ENSMUSG00000063406 | *Tmed5* | 902.0412 | 3.722854 | 0.009577 |
| ENSMUSG00000018362 | *Kpna2* | 2150.764 | 4.504024 | 0.009577 |
| ENSMUSG00000052331 | *Ankrd44* | 111.8214 | 4.124615 | 0.009595 |
| ENSMUSG00000026833 | *Olfm1* | 491.8948 | 3.630376 | 0.009649 |
| ENSMUSG00000030019 | *Fbxl14* | 1665.321 | 3.622049 | 0.009651 |
| ENSMUSG00000081692 | *Gm15971* | 31.73871 | 3.73435 | 0.009677 |
| ENSMUSG00000047446 | *Arl4a* | 418.3988 | 4.738077 | 0.009688 |
| ENSMUSG00000049281 | *Scn3b* | 28.83607 | 3.780809 | 0.009691 |
| ENSMUSG00000040904 | *Gm21988* | 80.57297 | 4.096367 | 0.009708 |
| ENSMUSG00000058793 | *Cds2* | 2401.965 | 4.005132 | 0.009716 |
| ENSMUSG00000036766 | *Dner* | 11.54201 | 3.276522 | 0.009724 |
| ENSMUSG00000034889 | *Cactin* | 4317.363 | 3.452175 | 0.009744 |
| ENSMUSG00000024042 | *Sik1* | 3220.362 | 3.054457 | 0.009757 |
| ENSMUSG00000019975 | *Ikbip* | 192.5682 | 4.472451 | 0.009758 |
| ENSMUSG00000032999 | *Nlrp4f* | 196.2219 | 4.592236 | 0.009768 |
| ENSMUSG00000042228 | *Lyn* | 64.41409 | 4.314 | 0.009777 |
| ENSMUSG00000031078 | *Cttn* | 10603.72 | 2.629397 | 0.009816 |
| ENSMUSG00000020806 | *Rhbdf2* | 8715.758 | 3.471408 | 0.009824 |
| ENSMUSG00000021540 | *Smad5* | 4312.479 | 4.16731 | 0.009846 |
| ENSMUSG00000045193 | *Cirbp* | 1193.69 | 4.053589 | 0.009856 |
| ENSMUSG00000022367 | *Has2* | 297.4289 | 4.697766 | 0.009865 |
| ENSMUSG00000025578 | *Cbx8* | 552.201 | 4.288989 | 0.009869 |
| ENSMUSG00000053931 | *Cnn3* | 10680.73 | 2.873225 | 0.009875 |
| ENSMUSG00000031483 | *Erlin2* | 2205.379 | 3.597785 | 0.009897 |
| ENSMUSG00000058407 | *Txndc9* | 544.5936 | 4.447598 | 0.009905 |
| ENSMUSG00000001418 | *Glmp* | 3727.027 | 3.672428 | 0.009924 |
| ENSMUSG00000021510 | *Zfp729a* | 1790.523 | 4.010458 | 0.009928 |
| ENSMUSG00000032577 | *Mapkapk3* | 1052.056 | 3.986306 | 0.009935 |
| ENSMUSG00000005973 | *Rcn1* | 3322.753 | 3.929643 | 0.009951 |
| ENSMUSG00000018501 | *Ncor1* | 9141.19 | 2.85997 | 0.009957 |
| ENSMUSG00000001288 | *Rarg* | 427.6483 | 4.199952 | 0.00996 |
| ENSMUSG00000031257 | *Nox1* | 5.114958 | 2.655686 | 0.009962 |
| ENSMUSG00000025332 | *Kdm5c* | 8757.196 | 3.005072 | 0.009966 |
| ENSMUSG00000021520 | *Uqcrb* | 156.7893 | 4.735526 | 0.009984 |
| ENSMUSG00000035476 | *Tab3* | 260.2554 | 3.993318 | 0.010002 |
| ENSMUSG00000034595 | *Ppp1r18* | 4959.954 | 3.339408 | 0.010034 |
| ENSMUSG00000029672 | *Fam3c* | 3559.569 | 3.728124 | 0.010077 |
| ENSMUSG00000043284 | *Tmem11* | 667.0696 | 4.298387 | 0.010096 |
| ENSMUSG00000025979 | *Mob4* | 87.56842 | 3.864553 | 0.010111 |
| ENSMUSG00000043969 | *Emx2* | 742.679 | 4.461468 | 0.010129 |
| ENSMUSG00000039770 | *Ypel5* | 322.752 | 4.382632 | 0.01013 |
| ENSMUSG00000030551 | *Nr2f2* | 1819.577 | 3.810143 | 0.01014 |
| ENSMUSG00000040385 | *Ppp1ca* | 5060.244 | 3.361734 | 0.010142 |
| ENSMUSG00000017485 | *Top2b* | 1644.879 | 3.37239 | 0.010149 |
| ENSMUSG00000051391 | *Ywhag* | 24486.84 | 2.580279 | 0.010167 |
| ENSMUSG00000083339 | *Gm11693* | 5.553641 | 2.780175 | 0.010211 |
| ENSMUSG00000027737 | *Slc7a11* | 1196.44 | 3.952651 | 0.010217 |
| ENSMUSG00000032280 | *Tle3* | 5716.921 | 2.486829 | 0.010266 |
| ENSMUSG00000024687 | *Osbp* | 5743.234 | 3.438204 | 0.010335 |
| ENSMUSG00000029625 | *Cpsf4* | 994.5593 | 3.874046 | 0.010347 |
| ENSMUSG00000032309 | *Fbxo22* | 770.1148 | 4.843657 | 0.010374 |
| ENSMUSG00000081559 | *Gm12411* | 424.9143 | 6.367359 | 0.010375 |
| ENSMUSG00000026678 | *Rgs5* | 9.160868 | 2.973556 | 0.010391 |
| ENSMUSG00000020363 | *Gfpt2* | 2864.939 | 4.49135 | 0.010391 |
| ENSMUSG00000115730 | *AC106830* | 8.087909 | 3.310954 | 0.010396 |
| ENSMUSG00000025794 | *Rpl14* | 334.2026 | 3.059311 | 0.010398 |
| ENSMUSG00000029175 | *Slc35f6* | 884.4222 | 3.593949 | 0.010431 |
| ENSMUSG00000021259 | *Cyp46a1* | 4.585033 | 2.678853 | 0.010483 |
| ENSMUSG00000031451 | *Gas6* | 5391.324 | 3.674299 | 0.010484 |
| ENSMUSG00000029432 | *Nipsnap2* | 2442.81 | 3.812415 | 0.010497 |
| ENSMUSG00000029778 | *Adcyap1r1* | 31.22458 | 3.636483 | 0.010514 |
| ENSMUSG00000001844 | *Zdhhc4* | 486.5482 | 4.090993 | 0.01053 |
| ENSMUSG00000081497 | *Gm15560* | 105.0467 | 4.669779 | 0.010577 |
| ENSMUSG00000043329 | *Gm8849* | 5.709007 | 2.769069 | 0.010611 |
| ENSMUSG00000039084 | *Chad* | 31.71265 | 3.740018 | 0.010635 |
| ENSMUSG00000027984 | *Hadh* | 1644.019 | 3.53421 | 0.010638 |
| ENSMUSG00000027087 | *Itgav* | 12402.66 | 2.980158 | 0.010638 |
| ENSMUSG00000041734 | *Kirrel* | 1367.046 | 3.338917 | 0.010651 |
| ENSMUSG00000018750 | *Zbtb4* | 2342.154 | 3.456756 | 0.010661 |
| ENSMUSG00000025246 | *Tbl1x* | 2957.546 | 3.348736 | 0.010677 |
| ENSMUSG00000040820 | *Hlcs* | 1474.042 | 4.155321 | 0.010705 |
| ENSMUSG00000022125 | *Cln5* | 1068.321 | 4.464426 | 0.010714 |
| ENSMUSG00000060938 | *Rpl26* | 11578.99 | 3.23673 | 0.01075 |
| ENSMUSG00000029328 | *Hnrnpdl* | 5013.883 | 3.812227 | 0.010763 |
| ENSMUSG00000070343 | *Gm10288* | 1819.893 | 6.125129 | 0.010807 |
| ENSMUSG00000028466 | *Creb3* | 3783.108 | 3.448792 | 0.010819 |
| ENSMUSG00000030265 | *Kras* | 1728.7 | 3.72127 | 0.010861 |
| ENSMUSG00000022543 | *4930451G09Rik* | 10.09376 | 3.497815 | 0.010864 |
| ENSMUSG00000029434 | *Vps33a* | 5342.023 | 3.904175 | 0.01089 |
| ENSMUSG00000037461 | *Ints7* | 2975.671 | 3.579711 | 0.010893 |
| ENSMUSG00000005873 | *Reep5* | 1272.161 | 3.930713 | 0.0109 |
| ENSMUSG00000012429 | *Mplkip* | 545.0324 | 3.955603 | 0.010976 |
| ENSMUSG00000024851 | *Pitpnm1* | 11200.41 | 2.978904 | 0.010985 |
| ENSMUSG00000008734 | *Gprc5b* | 4373.756 | 3.697973 | 0.010997 |
| ENSMUSG00000040621 | *Gemin8* | 180.5689 | 4.364523 | 0.011058 |
| ENSMUSG00000020745 | *Pafah1b1* | 4632.05 | 3.619773 | 0.011069 |
| ENSMUSG00000018263 | *Tbx5* | 164.5425 | 4.200717 | 0.011093 |
| ENSMUSG00000002870 | *Mcm2* | 5610.494 | 3.451478 | 0.011112 |
| ENSMUSG00000039427 | *Alg1* | 325.0723 | 4.415706 | 0.011129 |
| ENSMUSG00000002983 | *Relb* | 187.569 | 4.303588 | 0.011135 |
| ENSMUSG00000037656 | *Slc20a2* | 5278.713 | 3.18584 | 0.011138 |
| ENSMUSG00000002204 | *Napsa* | 16.93145 | 3.207926 | 0.011158 |
| ENSMUSG00000042680 | *Garem1* | 378.4795 | 4.43075 | 0.01116 |
| ENSMUSG00000030533 | *Unc45a* | 5236.868 | 2.35753 | 0.011225 |
| ENSMUSG00000054000 | *Tusc1* | 31.65321 | 3.663898 | 0.011245 |
| ENSMUSG00000024742 | *Fen1* | 855.8572 | 4.150907 | 0.011253 |
| ENSMUSG00000040935 | *Padi6* | 700.2144 | 4.119737 | 0.011391 |
| ENSMUSG00000019996 | *Map7* | 1453.419 | 4.040847 | 0.011401 |
| ENSMUSG00000027447 | *Cst3* | 5159.552 | 3.881637 | 0.011417 |
| ENSMUSG00000027706 | *Sec62* | 7008.356 | 2.684189 | 0.011436 |
| ENSMUSG00000022885 | *St6gal1* | 2161.6 | 2.972118 | 0.011518 |
| ENSMUSG00000027881 | *Prpf38b* | 29492.21 | 2.965536 | 0.011521 |
| ENSMUSG00000020697 | *Lig3* | 1143.22 | 3.142524 | 0.011521 |
| ENSMUSG00000027509 | *Rae1* | 639.7183 | 3.627516 | 0.011553 |
| ENSMUSG00000047286 | *Olfr370* | 39.49053 | 3.443047 | 0.011565 |
| ENSMUSG00000030249 | *Abcc9* | 441.7129 | 4.506961 | 0.011583 |
| ENSMUSG00000070282 | *3000002C10Rik* | 105.0238 | 4.062876 | 0.011626 |
| ENSMUSG00000034875 | *Nudt19* | 130.5894 | 4.273426 | 0.011627 |
| ENSMUSG00000027636 | *Sla2* | 10.02226 | 3.240356 | 0.011642 |
| ENSMUSG00000026786 | *Apbb1ip* | 12.66937 | 3.413883 | 0.011657 |
| ENSMUSG00000025933 | *Tmem14a* | 13.42785 | 3.347969 | 0.011666 |
| ENSMUSG00000039982 | *Dtx4* | 93.21861 | 3.997063 | 0.011677 |
| ENSMUSG00000056536 | *Pign* | 3744.689 | 3.658551 | 0.011677 |
| ENSMUSG00000029736 | *Nobox* | 6.972212 | 2.85191 | 0.011734 |
| ENSMUSG00000061878 | *Sphk1* | 243.4121 | 4.662452 | 0.01175 |
| ENSMUSG00000037868 | *Egr2* | 115.9239 | 4.450094 | 0.011768 |
| ENSMUSG00000025733 | *Rhot2* | 6205.99 | 3.371502 | 0.011796 |
| ENSMUSG00000004207 | *Psap* | 28438.49 | 2.272064 | 0.011809 |
| ENSMUSG00000036120 | *Rfxank* | 2548.995 | 4.709755 | 0.011815 |
| ENSMUSG00000083992 | *Gm11478* | 11.3178 | 2.600996 | 0.011837 |
| ENSMUSG00000000804 | *Usp32* | 828.9036 | 3.39883 | 0.011844 |
| ENSMUSG00000072812 | *Ahnak2* | 792.1689 | 4.698071 | 0.011936 |
| ENSMUSG00000073664 | *Nbeal1* | 3338.221 | 3.580326 | 0.011952 |
| ENSMUSG00000052516 | *Robo2* | 8495.741 | 3.464144 | 0.012055 |
| ENSMUSG00000066877 | *Nck2* | 2472.925 | 4.142589 | 0.012109 |
| ENSMUSG00000060131 | *Atp8b4* | 6.136706 | 2.769859 | 0.012168 |
| ENSMUSG00000112516 | *AC153522* | 142.2199 | 4.399452 | 0.012192 |
| ENSMUSG00000034292 | *Traf3ip1* | 1953.811 | 4.259665 | 0.012223 |
| ENSMUSG00000033760 | *Rbm4b* | 1624.491 | 3.954376 | 0.012271 |
| ENSMUSG00000017288 | *Vps53* | 849.0303 | 3.864909 | 0.012291 |
| ENSMUSG00000037887 | *Dusp8* | 465.1068 | 4.887608 | 0.012291 |
| ENSMUSG00000041052 | *Slc7a13* | 225.847 | 5.02472 | 0.012301 |
| ENSMUSG00000024287 | *Thoc1* | 67.17887 | 3.855538 | 0.01234 |
| ENSMUSG00000025779 | *Ly96* | 34.13189 | 4.220938 | 0.012341 |
| ENSMUSG00000018446 | *C1qbp* | 165.5863 | 3.628765 | 0.012423 |
| ENSMUSG00000046897 | *Zfp740* | 1728.722 | 3.322469 | 0.012429 |
| ENSMUSG00000023169 | *Slc38a1* | 9950.01 | 2.481553 | 0.012432 |
| ENSMUSG00000028745 | *Capzb* | 5024.022 | 2.969176 | 0.012481 |
| ENSMUSG00000080076 | *Hist1h2aj* | 204.0137 | 4.509276 | 0.012511 |
| ENSMUSG00000022257 | *Laptm4b* | 1620.732 | 3.038781 | 0.012545 |
| ENSMUSG00000026175 | *Vil1* | 235.5895 | 4.306833 | 0.012598 |
| ENSMUSG00000019370 | *Calm3* | 7796.294 | 2.647162 | 0.012636 |
| ENSMUSG00000061048 | *Cdh3* | 45.10736 | 4.17524 | 0.012646 |
| ENSMUSG00000032481 | *Smarcc1* | 2946.595 | 3.036926 | 0.012666 |
| ENSMUSG00000001493 | *Meox1* | 4.35576 | 2.460663 | 0.012671 |
| ENSMUSG00000000149 | *Gna12* | 5687.397 | 3.324943 | 0.012713 |
| ENSMUSG00000027012 | *Dync1i2* | 1709.066 | 3.389524 | 0.012713 |
| ENSMUSG00000044345 | *Marveld1* | 7733.738 | 4.571678 | 0.012715 |
| ENSMUSG00000040681 | *Hmgn1* | 6877.867 | 2.837969 | 0.012734 |
| ENSMUSG00000112178 | *AC164629* | 53.26913 | 4.591978 | 0.012737 |
| ENSMUSG00000004263 | *Atn1* | 3369.427 | 3.444466 | 0.012768 |
| ENSMUSG00000025742 | *Prps2* | 5391.304 | 4.7389 | 0.012774 |
| ENSMUSG00000040111 | *Gramd1b* | 43819.98 | 2.110814 | 0.012794 |
| ENSMUSG00000019978 | *Epb41l2* | 6350.957 | 3.034027 | 0.012796 |
| ENSMUSG00000030761 | *Myo7a* | 31007.39 | 2.899571 | 0.012829 |
| ENSMUSG00000037197 | *Rbm17* | 4050.437 | 2.842483 | 0.012843 |
| ENSMUSG00000019699 | *Akt3* | 745.6365 | 3.376889 | 0.012924 |
| ENSMUSG00000087034 | *Cbfa2t2* | 31.88349 | 3.394926 | 0.013011 |
| ENSMUSG00000113949 | *Scamp4* | 849.2712 | 4.23947 | 0.013031 |
| ENSMUSG00000029998 | *Pcyox1* | 10446.19 | 3.445919 | 0.013165 |
| ENSMUSG00000030994 | *D7Ertd443e* | 74.03557 | 4.039153 | 0.013201 |
| ENSMUSG00000026471 | *Mr1* | 60.75481 | 3.410767 | 0.013201 |
| ENSMUSG00000051316 | *Taf7* | 34.14483 | 4.072359 | 0.013248 |
| ENSMUSG00000038023 | *Atp6v0a2* | 2293.824 | 3.240052 | 0.013332 |
| ENSMUSG00000020044 | *Timp3* | 1404.328 | 2.515126 | 0.013431 |
| ENSMUSG00000032534 | *Cep63* | 484.5044 | 3.870446 | 0.013451 |
| ENSMUSG00000050705 | *2310061I04Rik* | 403.9429 | 4.528234 | 0.013545 |
| ENSMUSG00000040720 | *Virma* | 8928.12 | 3.226941 | 0.013602 |
| ENSMUSG00000071866 | *Ppia* | 2603.721 | 3.099842 | 0.013611 |
| ENSMUSG00000070493 | *Chchd2* | 975.8897 | 3.549011 | 0.013617 |
| ENSMUSG00000031072 | *Oraov1* | 141.7166 | 3.718281 | 0.013654 |
| ENSMUSG00000045098 | *Kmt5b* | 2955.711 | 3.372471 | 0.013666 |
| ENSMUSG00000042406 | *Atf4* | 5694.866 | 3.217192 | 0.013666 |
| ENSMUSG00000080977 | *Gm13772* | 6.546561 | 2.932909 | 0.013673 |
| ENSMUSG00000031502 | *Col4a1* | 15175.07 | 2.411173 | 0.013674 |
| ENSMUSG00000020530 | *Ggnbp2* | 1414.889 | 3.162389 | 0.013675 |
| ENSMUSG00000020257 | *Wdr82* | 2643.162 | 4.156406 | 0.013681 |
| ENSMUSG00000025290 | *Rps24* | 4367.067 | 2.734398 | 0.013718 |
| ENSMUSG00000028772 | *Zcchc17* | 191.0108 | 4.396975 | 0.013757 |
| ENSMUSG00000020098 | *Pcbd1* | 57.2704 | 3.349845 | 0.013766 |
| ENSMUSG00000038014 | *Fam120a* | 14254.13 | 2.248274 | 0.013829 |
| ENSMUSG00000006998 | *Psmd2* | 6422.889 | 2.980587 | 0.013868 |
| ENSMUSG00000038384 | *Setd1b* | 6683.666 | 2.616096 | 0.013879 |
| ENSMUSG00000020694 | *Tlk2* | 3017.241 | 3.669551 | 0.013881 |
| ENSMUSG00000028484 | *Psip1* | 3188.902 | 3.36297 | 0.013969 |
| ENSMUSG00000021263 | *Degs2* | 6.824001 | 2.661129 | 0.013996 |
| ENSMUSG00000113512 | *Gm9063* | 4.646354 | 2.569054 | 0.014014 |
| ENSMUSG00000057762 | *Gm6169* | 585.1173 | 4.315171 | 0.014015 |
| ENSMUSG00000026219 | *Trip12* | 5582.849 | 2.501665 | 0.01406 |
| ENSMUSG00000042709 | *Atpaf2* | 224.6704 | 3.445798 | 0.014079 |
| ENSMUSG00000021843 | *Ktn1* | 8550.142 | 3.24342 | 0.014095 |
| ENSMUSG00000031885 | *Cbfb* | 552.1441 | 3.737545 | 0.014108 |
| ENSMUSG00000007603 | *Dus3l* | 2160.768 | 3.724741 | 0.014131 |
| ENSMUSG00000054720 | *Lrrc8c* | 2456.501 | 3.202967 | 0.014153 |
| ENSMUSG00000073737 | *Gm10566* | 7.60113 | 2.547342 | 0.014274 |
| ENSMUSG00000019158 | *Tmem160* | 521.1689 | 4.312673 | 0.014294 |
| ENSMUSG00000028601 | *Echdc2* | 1821.857 | 3.097732 | 0.014298 |
| ENSMUSG00000024982 | *Zdhhc6* | 2548.409 | 3.604395 | 0.014299 |
| ENSMUSG00000073079 | *Srp54a* | 2033.062 | 3.963827 | 0.014315 |
| ENSMUSG00000028517 | *Plpp3* | 3578.503 | 3.045175 | 0.014339 |
| ENSMUSG00000067787 | *Blcap* | 1345.358 | 4.034511 | 0.014385 |
| ENSMUSG00000069265 | *Hist1h3a* | 651.3476 | 3.592442 | 0.014391 |
| ENSMUSG00000081896 | *Gm5389* | 38.44508 | 3.56232 | 0.014396 |
| ENSMUSG00000022365 | *Derl1* | 2772.632 | 3.130428 | 0.014447 |
| ENSMUSG00000029560 | *Snx8* | 2957.278 | 3.882537 | 0.014513 |
| ENSMUSG00000069237 | *Fam8a1* | 2862.37 | 4.073597 | 0.014547 |
| ENSMUSG00000004677 | *Myo9b* | 8433.576 | 2.543028 | 0.014575 |
| ENSMUSG00000029306 | *Ibsp* | 12.98129 | 2.965068 | 0.014619 |
| ENSMUSG00000030082 | *Sec61a1* | 13117.95 | 2.466299 | 0.014635 |
| ENSMUSG00000001098 | *Kctd10* | 2563.491 | 3.426838 | 0.014669 |
| ENSMUSG00000079509 | *Zfx* | 1801.353 | 3.998757 | 0.014731 |
| ENSMUSG00000008859 | *Rala* | 2335.841 | 4.070998 | 0.014786 |
| ENSMUSG00000041688 | *Amot* | 1240.878 | 3.435545 | 0.014809 |
| ENSMUSG00000041078 | *Grid1* | 103.0373 | 3.373311 | 0.014812 |
| ENSMUSG00000049225 | *Pdp1* | 127.775 | 3.937401 | 0.014814 |
| ENSMUSG00000047910 | *Pcdhb16* | 50.36447 | 3.571689 | 0.014851 |
| ENSMUSG00000054555 | *Adam12* | 3059.542 | 3.727347 | 0.014877 |
| ENSMUSG00000021929 | *Kpna3* | 576.8322 | 3.507921 | 0.014879 |
| ENSMUSG00000072294 | *Klf12* | 291.3492 | 3.800752 | 0.014935 |
| ENSMUSG00000027006 | *Dnajc10* | 1278.619 | 3.966399 | 0.014942 |
| ENSMUSG00000005161 | *Prdx2* | 4068.293 | 2.271 | 0.01497 |
| ENSMUSG00000031996 | *Aplp2* | 43430.35 | 2.041975 | 0.015056 |
| ENSMUSG00000038267 | *Slc22a23* | 69.49575 | 3.907262 | 0.015086 |
| ENSMUSG00000028104 | *Polr3gl* | 4715.22 | 3.917655 | 0.015087 |
| ENSMUSG00000070639 | *Lrrc8b* | 1628.673 | 3.464803 | 0.015148 |
| ENSMUSG00000004931 | *Apba3* | 656.3656 | 3.28545 | 0.015174 |
| ENSMUSG00000037058 | *Paip2* | 1750.083 | 3.393183 | 0.0152 |
| ENSMUSG00000004980 | *Hnrnpa2b1* | 51027.82 | 2.706927 | 0.015204 |
| ENSMUSG00000034675 | *Dbn1* | 15176.98 | 3.179846 | 0.015208 |
| ENSMUSG00000020272 | *Stk10* | 303.2559 | 4.054622 | 0.015239 |
| ENSMUSG00000045327 | *6330549D23Rik* | 36.5364 | 3.571241 | 0.015261 |
| ENSMUSG00000033389 | *Arhgap44* | 200.4646 | 4.166731 | 0.015289 |
| ENSMUSG00000032051 | *Fdx1* | 11690.46 | 2.660077 | 0.015339 |
| ENSMUSG00000035828 | *Pim3* | 2699.766 | 3.671402 | 0.015379 |
| ENSMUSG00000029062 | *Cdk11b* | 8498.413 | 3.14557 | 0.015405 |
| ENSMUSG00000027245 | *Hypk* | 511.9532 | 4.535768 | 0.015425 |
| ENSMUSG00000030782 | *Tgfb1i1* | 1842.936 | 3.136937 | 0.015495 |
| ENSMUSG00000036686 | *Cc2d1a* | 2786.884 | 3.237386 | 0.015607 |
| ENSMUSG00000066306 | *Numa1* | 7502.433 | 2.902543 | 0.015619 |
| ENSMUSG00000041763 | *Tpp2* | 1498.24 | 3.888851 | 0.015629 |
| ENSMUSG00000054136 | *Adm2* | 42.0147 | 4.169299 | 0.01569 |
| ENSMUSG00000026820 | *Ptges2* | 1439.752 | 4.506368 | 0.015707 |
| ENSMUSG00000030697 | *Ppp4c* | 1182.135 | 3.400455 | 0.015707 |
| ENSMUSG00000023034 | *Nr4a1* | 5178.68 | 2.734364 | 0.015747 |
| ENSMUSG00000038312 | *Edem2* | 1292.068 | 4.156298 | 0.015768 |
| ENSMUSG00000009073 | *Nf2* | 4201.992 | 3.253243 | 0.015791 |
| ENSMUSG00000039715 | *Wdr34* | 378.5371 | 3.99787 | 0.015838 |
| ENSMUSG00000021192 | *Golga5* | 442.175 | 3.699502 | 0.015883 |
| ENSMUSG00000028465 | *Tln1* | 23026.96 | 2.006795 | 0.015909 |
| ENSMUSG00000022010 | *Tsc22d1* | 19119.34 | 2.248017 | 0.015965 |
| ENSMUSG00000038145 | *Snrk* | 2963.111 | 3.758702 | 0.015978 |
| ENSMUSG00000030220 | *Arhgdib* | 6.159272 | 2.811698 | 0.015981 |
| ENSMUSG00000028560 | *Usp1* | 737.9084 | 4.296156 | 0.015987 |
| ENSMUSG00000091537 | *Tma7* | 932.9072 | 3.230609 | 0.015996 |
| ENSMUSG00000035126 | *Wdr78* | 13.86202 | 2.838105 | 0.016 |
| ENSMUSG00000020260 | *Pofut2* | 8006.086 | 2.181859 | 0.016018 |
| ENSMUSG00000024360 | *Etf1* | 1808.426 | 3.299331 | 0.016023 |
| ENSMUSG00000039159 | *Ube2h* | 8573.516 | 3.310412 | 0.016036 |
| ENSMUSG00000084883 | *Ccdc85c* | 295.1935 | 3.829147 | 0.016066 |
| ENSMUSG00000060166 | *Zdhhc8* | 2051.924 | 3.494199 | 0.016172 |
| ENSMUSG00000103906 | *Tigd5* | 575.0633 | 3.994954 | 0.016188 |
| ENSMUSG00000031925 | *Maml2* | 791.5831 | 3.73825 | 0.016233 |
| ENSMUSG00000037316 | *Bag4* | 101.196 | 3.798371 | 0.016238 |
| ENSMUSG00000081892 | *Gm8864* | 5.802029 | 2.50627 | 0.016245 |
| ENSMUSG00000028403 | *Zdhhc21* | 1168.032 | 3.313926 | 0.016255 |
| ENSMUSG00000090891 | *D6Ertd527e* | 417.7724 | 3.787914 | 0.016256 |
| ENSMUSG00000035941 | *Ibtk* | 1085.563 | 3.216099 | 0.016263 |
| ENSMUSG00000114779 | *AC160999* | 183.945 | 4.594895 | 0.016302 |
| ENSMUSG00000040767 | *Snrnp25* | 1254.158 | 5.529962 | 0.016331 |
| ENSMUSG00000026880 | *Stom* | 377.5691 | 4.122153 | 0.016332 |
| ENSMUSG00000018774 | *Cd68* | 340.658 | 4.383986 | 0.016356 |
| ENSMUSG00000022568 | *Scrib* | 12949.47 | 3.079547 | 0.016381 |
| ENSMUSG00000020273 | *Papolg* | 329.5106 | 3.571612 | 0.016399 |
| ENSMUSG00000071414 | *Gm6736* | 13.50537 | 3.174461 | 0.016459 |
| ENSMUSG00000022637 | *Cblb* | 5728.585 | 3.420593 | 0.016471 |
| ENSMUSG00000032215 | *Rsl24d1* | 2260.318 | 3.789139 | 0.016477 |
| ENSMUSG00000004934 | *Pias4* | 4018.428 | 3.480724 | 0.016482 |
| ENSMUSG00000026043 | *Col3a1* | 19689.72 | 4.056226 | 0.016504 |
| ENSMUSG00000111273 | *Olfr789* | 8.340906 | 3.378931 | 0.016533 |
| ENSMUSG00000036078 | *Sigmar1* | 1658.039 | 5.225326 | 0.016562 |
| ENSMUSG00000046262 | *C87977* | 228.3343 | 4.155978 | 0.016579 |
| ENSMUSG00000002900 | *Lamb1* | 13658.62 | 3.013346 | 0.016752 |
| ENSMUSG00000038170 | *Pde4dip* | 1191.322 | 3.5294 | 0.016752 |
| ENSMUSG00000002985 | *Apoe* | 2055.399 | 3.398615 | 0.016773 |
| ENSMUSG00000079277 | *Hoxd3* | 107.6238 | 3.749228 | 0.016798 |
| ENSMUSG00000017615 | *Tnfaip1* | 2541.149 | 3.54689 | 0.016816 |
| ENSMUSG00000039634 | *Zfp189* | 217.8168 | 4.158078 | 0.016825 |
| ENSMUSG00000074063 | *Osgin1* | 635.7548 | 3.154551 | 0.016858 |
| ENSMUSG00000040725 | *Hnrnpul1* | 11943.91 | 2.934925 | 0.016862 |
| ENSMUSG00000096099 | *Vmn1r220* | 5.335113 | 2.68429 | 0.016875 |
| ENSMUSG00000074676 | *Foxs1* | 17.74868 | 3.419823 | 0.016886 |
| ENSMUSG00000089774 | *Slc5a3* | 1192.325 | 3.408542 | 0.016936 |
| ENSMUSG00000027955 | *Fam198b* | 699.1272 | 4.137189 | 0.016981 |
| ENSMUSG00000031311 | *Nono* | 11381.99 | 2.802607 | 0.016997 |
| ENSMUSG00000034724 | *Cnot6l* | 924.8572 | 4.209572 | 0.017088 |
| ENSMUSG00000034837 | *Gnat1* | 6.32862 | 2.909864 | 0.017088 |
| ENSMUSG00000111483 | *Gm18997* | 517.5269 | 4.660367 | 0.017109 |
| ENSMUSG00000030982 | *9030624J02Rik* | 8205.734 | 3.159864 | 0.017197 |
| ENSMUSG00000056501 | *Cebpb* | 2468.013 | 3.060658 | 0.017205 |
| ENSMUSG00000025499 | *Hras* | 2375.837 | 4.396747 | 0.017215 |
| ENSMUSG00000026822 | *Lcn2* | 5.309845 | 2.591986 | 0.017231 |
| ENSMUSG00000024924 | *Vldlr* | 10936.08 | 3.120309 | 0.017256 |
| ENSMUSG00000055320 | *Tead1* | 6360.514 | 3.037868 | 0.017265 |
| ENSMUSG00000029816 | *Gpnmb* | 3677.09 | 4.74727 | 0.017309 |
| ENSMUSG00000039531 | *Zufsp* | 2328.575 | 3.929327 | 0.017349 |
| ENSMUSG00000061315 | *Naca* | 5396.178 | 3.282824 | 0.017359 |
| ENSMUSG00000051351 | *Zfp46* | 4174.828 | 3.604452 | 0.017384 |
| ENSMUSG00000025505 | *Tmem80* | 224.5395 | 3.519844 | 0.017391 |
| ENSMUSG00000016319 | *Slc25a5* | 2918.931 | 2.625569 | 0.017395 |
| ENSMUSG00000044646 | *Zbtb7c* | 625.8481 | 4.289999 | 0.017406 |
| ENSMUSG00000035372 | *1810055G02Rik* | 1702.292 | 4.405698 | 0.017416 |
| ENSMUSG00000024644 | *Cndp2* | 12300.74 | 4.24099 | 0.017429 |
| ENSMUSG00000022280 | *Rnf19a* | 4651.855 | 3.623336 | 0.017443 |
| ENSMUSG00000043087 | *Olfr855* | 4.792814 | 2.505617 | 0.017464 |
| ENSMUSG00000026331 | *Slco6c1* | 4.863675 | 2.355697 | 0.017485 |
| ENSMUSG00000082965 | *Gm6754* | 5.70561 | 2.602804 | 0.017494 |
| ENSMUSG00000023067 | *Cdkn1a* | 3019.458 | 3.432909 | 0.017536 |
| ENSMUSG00000033128 | *Gga1* | 2142.31 | 2.540532 | 0.017554 |
| ENSMUSG00000032094 | *Cd3d* | 1037.188 | 3.929929 | 0.017612 |
| ENSMUSG00000038615 | *Nfe2l1* | 19370.18 | 2.14294 | 0.01764 |
| ENSMUSG00000029564 | *4930519G04Rik* | 5.933381 | 2.563501 | 0.01764 |
| ENSMUSG00000043252 | *Tmem64* | 1923.074 | 3.695913 | 0.017657 |
| ENSMUSG00000071470 | *Ccnb1ip1* | 13301.98 | 2.240065 | 0.017663 |
| ENSMUSG00000041203 | *Trir* | 1453.866 | 3.671922 | 0.017677 |
| ENSMUSG00000036572 | *Upf3b* | 927.8326 | 3.812278 | 0.017726 |
| ENSMUSG00000055745 | *Rtl6* | 257.6752 | 4.14322 | 0.017727 |
| ENSMUSG00000032239 | *Rp9* | 4261.974 | 3.468221 | 0.017802 |
| ENSMUSG00000057207 | *Olfr1028* | 25.66559 | 3.400838 | 0.017851 |
| ENSMUSG00000096546 | *Smlr1* | 5.951666 | 2.491257 | 0.01797 |
| ENSMUSG00000020719 | *Ddx5* | 6091.218 | 2.827345 | 0.018141 |
| ENSMUSG00000029455 | *Aldh2* | 7822.576 | 3.280901 | 0.018146 |
| ENSMUSG00000015759 | *Cnih1* | 142.9411 | 3.474045 | 0.018146 |
| ENSMUSG00000061701 | *Fbxw20* | 4.026971 | 2.4364 | 0.018166 |
| ENSMUSG00000024999 | *Noc3l* | 572.6667 | 3.251235 | 0.018242 |
| ENSMUSG00000110761 | *Gm29761* | 8.915609 | 3.116462 | 0.018306 |
| ENSMUSG00000057335 | *Cep170* | 1491.617 | 2.888653 | 0.018341 |
| ENSMUSG00000012123 | *Crybg2* | 33.0022 | 2.984247 | 0.018446 |
| ENSMUSG00000005469 | *Prkaca* | 1070.426 | 3.406824 | 0.018524 |
| ENSMUSG00000083280 | *Gm11838* | 9.00782 | 3.033424 | 0.018555 |
| ENSMUSG00000031765 | *Mt1* | 35924.86 | 3.144124 | 0.01856 |
| ENSMUSG00000029335 | *Bmp3* | 4464.848 | 3.651296 | 0.01859 |
| ENSMUSG00000021815 | *Mss51* | 14.89269 | 3.66315 | 0.018596 |
| ENSMUSG00000053754 | *Chd8* | 7600.568 | 3.024885 | 0.018598 |
| ENSMUSG00000001576 | *Ergic1* | 4954.702 | 3.641279 | 0.018614 |
| ENSMUSG00000026825 | *Dnm1* | 657.8574 | 4.478541 | 0.018615 |
| ENSMUSG00000040687 | *Madd* | 6408.908 | 4.539502 | 0.018618 |
| ENSMUSG00000034863 | *Ano8* | 2521.206 | 3.52211 | 0.018621 |
| ENSMUSG00000037692 | *Ahdc1* | 1822.208 | 3.139834 | 0.018621 |
| ENSMUSG00000026399 | *Cd55* | 139.3686 | 3.176905 | 0.018713 |
| ENSMUSG00000024732 | *Ccdc86* | 1622.746 | 3.3147 | 0.018737 |
| ENSMUSG00000075028 | *Prdm11* | 3779.403 | 3.55112 | 0.018768 |
| ENSMUSG00000039057 | *Myo16* | 207.7541 | 4.160284 | 0.018804 |
| ENSMUSG00000031827 | *Cotl1* | 2294.403 | 4.368403 | 0.018889 |
| ENSMUSG00000008690 | *Ncaph2* | 2968.882 | 3.317444 | 0.018913 |
| ENSMUSG00000030203 | *Dusp16* | 493.446 | 4.004908 | 0.018954 |
| ENSMUSG00000033161 | *Atp1a1* | 33057.5 | 1.742564 | 0.01897 |
| ENSMUSG00000022000 | *Zc3h13* | 28910.44 | 3.109253 | 0.018991 |
| ENSMUSG00000029869 | *Ephb6* | 2181.13 | 3.577079 | 0.018998 |
| ENSMUSG00000026473 | *Glul* | 11539.93 | 2.849141 | 0.019047 |
| ENSMUSG00000051022 | *Hs3st1* | 3509.719 | 3.210484 | 0.019066 |
| ENSMUSG00000028980 | *H6pd* | 8345.384 | 3.458322 | 0.019092 |
| ENSMUSG00000022427 | *Tomm22* | 527.7865 | 2.877127 | 0.019106 |
| ENSMUSG00000030729 | *Pgm2l1* | 699.0341 | 3.803909 | 0.019156 |
| ENSMUSG00000083621 | *Gm14586* | 68.8577 | 3.217226 | 0.019167 |
| ENSMUSG00000068205 | *Macrod2* | 22.19758 | 3.47701 | 0.019185 |
| ENSMUSG00000002052 | *Supt6* | 12169.22 | 2.198082 | 0.019209 |
| ENSMUSG00000024900 | *Cpt1a* | 8059.952 | 2.490823 | 0.01922 |
| ENSMUSG00000022678 | *Nde1* | 731.1715 | 3.759545 | 0.019238 |
| ENSMUSG00000066278 | *Vps37b* | 1309.595 | 4.279782 | 0.01927 |
| ENSMUSG00000049897 | *Stkld1* | 8.506892 | 2.833446 | 0.019328 |
| ENSMUSG00000108083 | *Mug4* | 14.41119 | 3.122138 | 0.019335 |
| ENSMUSG00000072708 | *Olfr381* | 10.47764 | 2.677133 | 0.019344 |
| ENSMUSG00000022983 | *Scaf4* | 3131.591 | 2.963576 | 0.019577 |
| ENSMUSG00000044293 | *Olfr794* | 8.728357 | 3.01204 | 0.019591 |
| ENSMUSG00000042608 | *Stk40* | 1016.873 | 3.362364 | 0.019669 |
| ENSMUSG00000020720 | *Psmd12* | 994.8945 | 3.962053 | 0.019688 |
| ENSMUSG00000002365 | *Snx9* | 3029.408 | 3.837946 | 0.019754 |
| ENSMUSG00000045672 | *Col27a1* | 3611.092 | 3.682602 | 0.019823 |
| ENSMUSG00000028637 | *Ccdc30* | 77.08496 | 4.12888 | 0.019951 |
| ENSMUSG00000027610 | *Gss* | 1227.557 | 4.05922 | 0.020055 |
| ENSMUSG00000044707 | *Ccnjl* | 1906.355 | 3.769336 | 0.020091 |
| ENSMUSG00000056458 | *Mok* | 43.05547 | 3.547622 | 0.020143 |
| ENSMUSG00000023043 | *Krt18* | 102.7882 | 4.047391 | 0.020172 |
| ENSMUSG00000042842 | *Serpinb6b* | 159.3118 | 3.030586 | 0.020222 |
| ENSMUSG00000045348 | *Nyap1* | 1649.61 | 4.45767 | 0.020282 |
| ENSMUSG00000009079 | *Ewsr1* | 27808.09 | 2.882224 | 0.020289 |
| ENSMUSG00000020721 | *Helz* | 10859.44 | 2.861281 | 0.020364 |
| ENSMUSG00000030663 | *1110004F10Rik* | 2561.204 | 3.783576 | 0.020379 |
| ENSMUSG00000069516 | *Lyz2* | 53.67824 | 3.389437 | 0.020442 |
| ENSMUSG00000053024 | *Cntn2* | 90.02078 | 4.026253 | 0.020445 |
| ENSMUSG00000041741 | *Pde3a* | 388.2411 | 4.096853 | 0.020459 |
| ENSMUSG00000024750 | *Zfand5* | 537.3593 | 3.501021 | 0.020461 |
| ENSMUSG00000035021 | *Baz1a* | 3633.244 | 3.523838 | 0.020476 |
| ENSMUSG00000015363 | *Trabd* | 2117.466 | 3.479806 | 0.020593 |
| ENSMUSG00000079635 | *Rhox4c* | 6.003129 | 2.550109 | 0.020683 |
| ENSMUSG00000074886 | *Grk6* | 2393.98 | 3.375264 | 0.020707 |
| ENSMUSG00000020715 | *Ern1* | 7107.677 | 2.821183 | 0.020795 |
| ENSMUSG00000020423 | *Btg2* | 827.0299 | 3.876699 | 0.020805 |
| ENSMUSG00000029684 | *Wasl* | 3273.143 | 2.444509 | 0.020807 |
| ENSMUSG00000059991 | *Nptx2* | 15.91979 | 3.219286 | 0.020831 |
| ENSMUSG00000027523 | *Gnas* | 16585.65 | 2.760078 | 0.020842 |
| ENSMUSG00000048234 | *Rnf149* | 5048.086 | 3.801256 | 0.020852 |
| ENSMUSG00000027496 | *Aurka* | 255.532 | 3.605828 | 0.020871 |
| ENSMUSG00000038224 | *Serpinf2* | 4.08076 | 2.239828 | 0.020888 |
| ENSMUSG00000028557 | *Rnf11* | 453.9228 | 3.655634 | 0.020932 |
| ENSMUSG00000035107 | *Dcbld2* | 8255.018 | 2.837992 | 0.020958 |
| ENSMUSG00000047945 | *Marcksl1* | 3092.608 | 3.1124 | 0.020981 |
| ENSMUSG00000037846 | *Rtkn2* | 176.2861 | 4.062913 | 0.021014 |
| ENSMUSG00000038764 | *Ptpn3* | 207.4348 | 3.868956 | 0.021053 |
| ENSMUSG00000115248 | *AC154649* | 463.455 | 3.568403 | 0.021057 |
| ENSMUSG00000030759 | *Far1* | 3258.127 | 2.933424 | 0.021096 |
| ENSMUSG00000075595 | *Zfp652* | 2346.07 | 3.25787 | 0.021104 |
| ENSMUSG00000026509 | *Capn2* | 1919.078 | 3.187805 | 0.02112 |
| ENSMUSG00000036568 | *Bicral* | 1783.759 | 3.607474 | 0.02113 |
| ENSMUSG00000021702 | *Thbs4* | 67953.42 | 2.711505 | 0.021132 |
| ENSMUSG00000046865 | *Fbl* | 8768.352 | 3.400699 | 0.021143 |
| ENSMUSG00000032179 | *Bmp5* | 164.9919 | 4.080086 | 0.021195 |
| ENSMUSG00000027134 | *Lpcat4* | 628.0174 | 3.59194 | 0.021212 |
| ENSMUSG00000024858 | *Grk2* | 2981.215 | 3.428925 | 0.021234 |
| ENSMUSG00000029345 | *Tfip11* | 2323.119 | 3.614704 | 0.021236 |
| ENSMUSG00000049807 | *Arhgap23* | 3263.058 | 3.48623 | 0.021253 |
| ENSMUSG00000060044 | *Tmem26* | 1150.614 | 4.380695 | 0.021369 |
| ENSMUSG00000081731 | *Calr* | 4.890685 | 2.377833 | 0.02138 |
| ENSMUSG00000111164 | *AC147565* | 11355.38 | 4.609085 | 0.021381 |
| ENSMUSG00000021270 | *Hsp90aa1* | 50538.9 | 2.923744 | 0.021451 |
| ENSMUSG00000034471 | *Caskin2* | 4491.198 | 2.894588 | 0.021477 |
| ENSMUSG00000054414 | *Slc30a7* | 1095.62 | 3.551134 | 0.021479 |
| ENSMUSG00000042743 | *Sgtb* | 265.4153 | 3.811025 | 0.021494 |
| ENSMUSG00000043572 | *Pars2* | 48.33476 | 3.208031 | 0.021518 |
| ENSMUSG00000021127 | *Zfp36l1* | 5682.711 | 2.962317 | 0.021564 |
| ENSMUSG00000021597 | *Slf1* | 22.08355 | 2.463797 | 0.021585 |
| ENSMUSG00000059195 | *Gm12715* | 24382.52 | 4.296513 | 0.021617 |
| ENSMUSG00000028680 | *Plk3* | 296.1917 | 4.687439 | 0.021691 |
| ENSMUSG00000049252 | *Lrp1b* | 4.571474 | 2.188523 | 0.021753 |
| ENSMUSG00000003363 | *Pld3* | 1633.586 | 2.888676 | 0.021769 |
| ENSMUSG00000024457 | *Trim26* | 17443.26 | 2.376442 | 0.021805 |
| ENSMUSG00000025485 | *Ric8a* | 4490.648 | 3.323802 | 0.021862 |
| ENSMUSG00000040782 | *Rfwd2* | 3523.051 | 3.812376 | 0.021902 |
| ENSMUSG00000022203 | *Efs* | 43.34129 | 3.47461 | 0.021916 |
| ENSMUSG00000015812 | *Gnrh1* | 9.04412 | 2.892508 | 0.021961 |
| ENSMUSG00000032171 | *Pin1* | 1927.431 | 3.918059 | 0.022026 |
| ENSMUSG00000016128 | *Stard13* | 1903.763 | 3.940067 | 0.022046 |
| ENSMUSG00000021910 | *Nisch* | 36248.94 | 1.954724 | 0.022087 |
| ENSMUSG00000060487 | *Samd5* | 1369.572 | 3.698772 | 0.022092 |
| ENSMUSG00000029415 | *Sdad1* | 2261.786 | 3.501959 | 0.022116 |
| ENSMUSG00000047090 | *Tmem198b* | 192.2443 | 4.118556 | 0.022159 |
| ENSMUSG00000059878 | *Zfp422* | 171.6862 | 3.357122 | 0.022162 |
| ENSMUSG00000021482 | *Aaed1* | 221.0454 | 3.189259 | 0.022234 |
| ENSMUSG00000020472 | *Zkscan17* | 3675.348 | 3.368833 | 0.022272 |
| ENSMUSG00000045780 | *Olfr624* | 9501.119 | 2.668397 | 0.022318 |
| ENSMUSG00000015757 | *Ppil4* | 318.7017 | 3.53208 | 0.022327 |
| ENSMUSG00000043279 | *Trim56* | 2453.304 | 3.737924 | 0.022332 |
| ENSMUSG00000045316 | *Fahd1* | 241.8661 | 4.115141 | 0.022351 |
| ENSMUSG00000038914 | *Dido1* | 4665.226 | 2.643662 | 0.022371 |
| ENSMUSG00000027510 | *Rbm38* | 341.2508 | 3.29389 | 0.022414 |
| ENSMUSG00000022285 | *Ywhaz* | 19989.95 | 2.517256 | 0.022561 |
| ENSMUSG00000091898 | *Tnnc1* | 1235.397 | 4.254446 | 0.022568 |
| ENSMUSG00000057113 | *Npm1* | 7931.365 | 2.613892 | 0.022591 |
| ENSMUSG00000089945 | *Pakap* | 5714.951 | 4.31241 | 0.022613 |
| ENSMUSG00000018411 | *Mapt* | 6.966614 | 2.405085 | 0.022616 |
| ENSMUSG00000066463 | *Omt2a* | 4.421065 | 2.383322 | 0.022625 |
| ENSMUSG00000024661 | *Fth1* | 11659.75 | 2.485974 | 0.022661 |
| ENSMUSG00000025326 | *Ube3a* | 2975.069 | 2.802859 | 0.022719 |
| ENSMUSG00000018604 | *Tbx3* | 36.76462 | 3.089599 | 0.022763 |
| ENSMUSG00000006315 | *Tmem147* | 1177.139 | 3.398294 | 0.022783 |
| ENSMUSG00000029223 | *Uchl1* | 3470.116 | 2.835479 | 0.022789 |
| ENSMUSG00000044813 | *Shb* | 182.4759 | 3.225617 | 0.022828 |
| ENSMUSG00000039648 | *Kyat1* | 628.3493 | 4.316732 | 0.022832 |
| ENSMUSG00000032244 | *Fem1b* | 933.1971 | 3.442569 | 0.022877 |
| ENSMUSG00000071262 | *Zfp957* | 8.181628 | 2.727136 | 0.022898 |
| ENSMUSG00000032397 | *Tipin* | 80.48754 | 3.270799 | 0.022961 |
| ENSMUSG00000031657 | *Heatr3* | 90.00572 | 3.053601 | 0.022969 |
| ENSMUSG00000031586 | *Rbpms* | 1454.215 | 3.199545 | 0.02299 |
| ENSMUSG00000059729 | *Olfr1385* | 1394.776 | 4.210349 | 0.023056 |
| ENSMUSG00000054169 | *Ceacam10* | 4.282213 | 2.236403 | 0.023128 |
| ENSMUSG00000053175 | *Bcl3* | 467.3375 | 3.87758 | 0.023166 |
| ENSMUSG00000020680 | *Taf15* | 5494.395 | 3.455157 | 0.023248 |
| ENSMUSG00000063457 | *Rps15* | 14619.54 | 2.71183 | 0.02326 |
| ENSMUSG00000051675 | *Trim32* | 1944.251 | 3.613525 | 0.023298 |
| ENSMUSG00000024240 | *Epc1* | 1790.403 | 2.972592 | 0.023313 |
| ENSMUSG00000042506 | *Usp22* | 6425.378 | 2.719125 | 0.023333 |
| ENSMUSG00000024505 | *Dtwd2* | 6.325407 | 2.642961 | 0.023361 |
| ENSMUSG00000031825 | *Crispld2* | 15520.55 | 2.903832 | 0.023394 |
| ENSMUSG00000024782 | *Ak3* | 1070.525 | 3.695956 | 0.023406 |
| ENSMUSG00000016256 | *Ctsz* | 4424.169 | 3.872661 | 0.023409 |
| ENSMUSG00000024440 | *Pcdh12* | 9.592511 | 2.412331 | 0.023418 |
| ENSMUSG00000020156 | *Mum1* | 1423.316 | 3.343376 | 0.023432 |
| ENSMUSG00000009863 | *Sdhb* | 2408.794 | 3.328509 | 0.023435 |
| ENSMUSG00000079445 | *B3gnt7* | 14.71991 | 3.163504 | 0.02355 |
| ENSMUSG00000043019 | *Edem3* | 3392.293 | 3.464571 | 0.023558 |
| ENSMUSG00000050965 | *Prkca* | 811.5488 | 3.033766 | 0.02358 |
| ENSMUSG00000022462 | *Slc38a2* | 24330.43 | 2.553951 | 0.02358 |
| ENSMUSG00000029178 | *Klf3* | 907.3604 | 2.154135 | 0.023581 |
| ENSMUSG00000045215 | *Asxl3* | 190.0397 | 4.205674 | 0.023645 |
| ENSMUSG00000054890 | *Olfr1535* | 11.89104 | 2.812562 | 0.023656 |
| ENSMUSG00000024413 | *Npc1* | 1525.703 | 3.359438 | 0.023697 |
| ENSMUSG00000056820 | *Tsnax* | 1614.897 | 4.211826 | 0.023775 |
| ENSMUSG00000028468 | *Rgp1* | 1455.374 | 2.930107 | 0.023809 |
| ENSMUSG00000017428 | *Psmd11* | 1713.473 | 2.809362 | 0.02381 |
| ENSMUSG00000029003 | *Mad2l2* | 127.3149 | 3.73134 | 0.023889 |
| ENSMUSG00000029482 | *Aacs* | 1373.118 | 4.000827 | 0.023902 |
| ENSMUSG00000001506 | *Col1a1* | 14833.63 | 3.066381 | 0.023914 |
| ENSMUSG00000058291 | *Zfp68* | 188.247 | 3.84308 | 0.023932 |
| ENSMUSG00000043088 | *Il17re* | 14.16194 | 2.86871 | 0.023962 |
| ENSMUSG00000032041 | *Tirap* | 285.2036 | 3.912503 | 0.023996 |
| ENSMUSG00000066113 | *Adamtsl1* | 325.1301 | 4.490201 | 0.024037 |
| ENSMUSG00000034205 | *Loxl2* | 23706.61 | 2.606678 | 0.024083 |
| ENSMUSG00000010277 | *2610507B11Rik* | 3929.913 | 2.899605 | 0.02409 |
| ENSMUSG00000053334 | *Ficd* | 568.7064 | 3.561021 | 0.024098 |
| ENSMUSG00000026814 | *Eng* | 127.165 | 3.625406 | 0.024101 |
| ENSMUSG00000029466 | *Anapc7* | 5274.238 | 3.367627 | 0.024189 |
| ENSMUSG00000057751 | *Megf6* | 882.2672 | 3.221115 | 0.02422 |
| ENSMUSG00000058446 | *Znrf2* | 929.7741 | 3.310256 | 0.024223 |
| ENSMUSG00000079465 | *Col4a3* | 368.1386 | 3.047917 | 0.024229 |
| ENSMUSG00000030556 | *Lrrc28* | 94.25599 | 3.357832 | 0.024354 |
| ENSMUSG00000009621 | *Vav2* | 420.5151 | 4.017568 | 0.024368 |
| ENSMUSG00000001783 | *Rtcb* | 2161.584 | 3.737433 | 0.024372 |
| ENSMUSG00000029556 | *Hnf1a* | 4.506867 | 2.513432 | 0.024422 |
| ENSMUSG00000035673 | *Sbno2* | 6922.666 | 2.599916 | 0.024445 |
| ENSMUSG00000041815 | *Poldip3* | 7649.236 | 2.630208 | 0.024495 |
| ENSMUSG00000053799 | *Exoc6* | 28.22285 | 3.652265 | 0.024593 |
| ENSMUSG00000024165 | *Jpt2* | 600.2784 | 3.663354 | 0.02461 |
| ENSMUSG00000042105 | *Inpp5f* | 3306.521 | 3.80636 | 0.024631 |
| ENSMUSG00000052912 | *Smarca5* | 125.8124 | 3.880808 | 0.024705 |
| ENSMUSG00000039395 | *Mreg* | 105.6708 | 3.889338 | 0.024862 |
| ENSMUSG00000022419 | *Deptor* | 587.7265 | 2.970154 | 0.024863 |
| ENSMUSG00000028954 | *Nub1* | 4880.542 | 3.272093 | 0.024885 |
| ENSMUSG00000034533 | *Scn10a* | 13.49683 | 2.7474 | 0.024949 |
| ENSMUSG00000035064 | *Eef2k* | 1739.767 | 3.681067 | 0.02495 |
| ENSMUSG00000045594 | *Glb1* | 3547.446 | 3.872785 | 0.024951 |
| ENSMUSG00000044147 | *Arf6* | 4403.848 | 2.950466 | 0.024985 |
| ENSMUSG00000091119 | *Ccdc152* | 4.858922 | 2.295049 | 0.024999 |
| ENSMUSG00000042505 | *Sdhaf3* | 33.55112 | 3.099038 | 0.025014 |
| ENSMUSG00000029028 | *Lrrc47* | 2636.556 | 3.044018 | 0.025082 |
| ENSMUSG00000042178 | *Armc5* | 1083.874 | 3.39316 | 0.025093 |
| ENSMUSG00000039221 | *Rpl22l1* | 11.85132 | 2.952869 | 0.025105 |
| ENSMUSG00000028760 | *Eif4g3* | 9766.548 | 2.271609 | 0.02513 |
| ENSMUSG00000026202 | *Tuba4a* | 568.1621 | 4.075656 | 0.025155 |
| ENSMUSG00000030738 | *Eif3c* | 10012.74 | 2.182194 | 0.025284 |
| ENSMUSG00000020132 | *Rab21* | 623.2976 | 3.48003 | 0.025302 |
| ENSMUSG00000036814 | *Slc6a20a* | 4.360748 | 2.100539 | 0.025345 |
| ENSMUSG00000005262 | *Ufd1* | 1008.473 | 2.938615 | 0.025406 |
| ENSMUSG00000037742 | *Eef1a1* | 56408.31 | 2.032321 | 0.025444 |
| ENSMUSG00000028821 | *Syf2* | 2608.812 | 3.385531 | 0.025489 |
| ENSMUSG00000006333 | *Rps9* | 7709.857 | 2.149657 | 0.025573 |
| ENSMUSG00000028010 | *Gar1* | 7171.589 | 4.122406 | 0.025579 |
| ENSMUSG00000020340 | *Cyfip2* | 4721.55 | 3.373585 | 0.025606 |
| ENSMUSG00000024400 | *Wdr33* | 1977.351 | 2.882422 | 0.025633 |
| ENSMUSG00000027400 | *Pdyn* | 5.291029 | 2.370661 | 0.02566 |
| ENSMUSG00000055723 | *Rras2* | 211.5641 | 3.830609 | 0.025666 |
| ENSMUSG00000026277 | *Stk25* | 2016.315 | 2.9658 | 0.025705 |
| ENSMUSG00000039148 | *Sart1* | 6699.723 | 3.056165 | 0.02575 |
| ENSMUSG00000032384 | *Csnk1g1* | 1621.68 | 3.045081 | 0.025829 |
| ENSMUSG00000021070 | *Bdkrb2* | 8.539695 | 3.045009 | 0.025894 |
| ENSMUSG00000024222 | *Fkbp5* | 12347.18 | 2.794744 | 0.025895 |
| ENSMUSG00000006307 | *Kmt2b* | 12429.94 | 2.256129 | 0.025901 |
| ENSMUSG00000013787 | *Ehmt2* | 20957.16 | 3.085304 | 0.025954 |
| ENSMUSG00000047045 | *Tmem164* | 3613.989 | 2.676349 | 0.025957 |
| ENSMUSG00000035530 | *Eif1* | 2951.958 | 2.388846 | 0.025999 |
| ENSMUSG00000020368 | *Canx* | 36928.2 | 2.661264 | 0.026001 |
| ENSMUSG00000052384 | *Nrros* | 70.46693 | 3.006363 | 0.026002 |
| ENSMUSG00000047417 | *Rexo1* | 4650.468 | 3.180963 | 0.026009 |
| ENSMUSG00000020755 | *Sap30bp* | 2540.765 | 3.614932 | 0.026094 |
| ENSMUSG00000022897 | *Dyrk1a* | 1988.753 | 2.929405 | 0.026103 |
| ENSMUSG00000060188 | *Cxcl17* | 5.359008 | 2.386668 | 0.026119 |
| ENSMUSG00000015766 | *Eps8* | 1073.14 | 4.134254 | 0.026218 |
| ENSMUSG00000027642 | *Rpn2* | 9487.378 | 2.604608 | 0.026225 |
| ENSMUSG00000020114 | *Cand1* | 4675.987 | 3.308555 | 0.026247 |
| ENSMUSG00000021932 | *Rnaseh2b* | 311.7037 | 3.925908 | 0.026296 |
| ENSMUSG00000003949 | *Hlf* | 48.70428 | 3.737466 | 0.026319 |
| ENSMUSG00000050010 | *Shisa3* | 666.5476 | 3.679311 | 0.026335 |
| ENSMUSG00000045983 | *Eif4g1* | 38554.96 | 2.071448 | 0.026385 |
| ENSMUSG00000006818 | *Sod2* | 413.3765 | 3.147225 | 0.026398 |
| ENSMUSG00000005813 | *Metap1* | 505.0405 | 3.000157 | 0.026418 |
| ENSMUSG00000033633 | *Clec18a* | 20.62993 | 2.976964 | 0.026479 |
| ENSMUSG00000046062 | *Ppp1r15b* | 1041.059 | 3.32434 | 0.026481 |
| ENSMUSG00000050174 | *Nudt6* | 8.383474 | 2.440427 | 0.026483 |
| ENSMUSG00000113993 | *AC163621* | 32.15192 | 3.266547 | 0.026522 |
| ENSMUSG00000099908 | *Gm28539* | 345.1592 | 4.237907 | 0.026562 |
| ENSMUSG00000114558 | *AC165148* | 8.391818 | 2.537422 | 0.026568 |
| ENSMUSG00000043733 | *Ptpn11* | 6472.049 | 3.217485 | 0.026575 |
| ENSMUSG00000032249 | *Anp32a* | 209508.7 | 4.157821 | 0.026747 |
| ENSMUSG00000024045 | *Akap8* | 6699.13 | 3.357162 | 0.026807 |
| ENSMUSG00000028439 | *Fam219a* | 1744.303 | 4.200748 | 0.026885 |
| ENSMUSG00000030120 | *Mlf2* | 7066.248 | 2.315559 | 0.026938 |
| ENSMUSG00000027351 | *Spred1* | 3309.524 | 3.400863 | 0.027005 |
| ENSMUSG00000025225 | *Nfkb2* | 411.9722 | 2.771762 | 0.027096 |
| ENSMUSG00000073433 | *Arhgdig* | 70.56775 | 3.647656 | 0.027109 |
| ENSMUSG00000079588 | *Tmem182* | 5.441635 | 2.479342 | 0.027139 |
| ENSMUSG00000021904 | *Sema3g* | 4793.968 | 3.642965 | 0.027151 |
| ENSMUSG00000003345 | *Csnk1g2* | 7510.818 | 3.012669 | 0.027166 |
| ENSMUSG00000046329 | *Slc25a23* | 3712.213 | 3.691454 | 0.027202 |
| ENSMUSG00000020038 | *Cry1* | 763.5463 | 3.941714 | 0.027208 |
| ENSMUSG00000091900 | *Gm4353* | 5.366481 | 2.234011 | 0.027329 |
| ENSMUSG00000055302 | *Mrfap1* | 3565.798 | 2.766096 | 0.027331 |
| ENSMUSG00000071342 | *Lsmem1* | 8.050238 | 2.737784 | 0.027373 |
| ENSMUSG00000050944 | *Efcab5* | 4.105062 | 2.138431 | 0.027404 |
| ENSMUSG00000031328 | *Flna* | 44512.88 | 1.91214 | 0.027453 |
| ENSMUSG00000027940 | *Tpm3* | 8735.443 | 2.598026 | 0.027538 |
| ENSMUSG00000031487 | *Brf2* | 100.7325 | 3.695364 | 0.027545 |
| ENSMUSG00000007617 | *Homer1* | 152.6722 | 2.507423 | 0.027668 |
| ENSMUSG00000031988 | *Vps26b* | 2748.434 | 3.93172 | 0.027758 |
| ENSMUSG00000026228 | *Htr2b* | 32.02722 | 3.172921 | 0.027765 |
| ENSMUSG00000054400 | *Cklf* | 3233.294 | 4.54538 | 0.027794 |
| ENSMUSG00000069014 | *Gm5641* | 2081.683 | 4.93947 | 0.027822 |
| ENSMUSG00000024392 | *Bag6* | 16882.06 | 2.53802 | 0.027847 |
| ENSMUSG00000063362 | *Alg11* | 897.0659 | 3.669674 | 0.02785 |
| ENSMUSG00000003549 | *Ercc1* | 471.4851 | 3.244316 | 0.027877 |
| ENSMUSG00000031441 | *Atp11a* | 8874.355 | 3.120309 | 0.027918 |
| ENSMUSG00000102918 | *Pcdhgc3* | 1986.131 | 3.213181 | 0.027977 |
| ENSMUSG00000069867 | *Pabpn1l* | 74.54735 | 3.17096 | 0.027983 |
| ENSMUSG00000003062 | *Stard3nl* | 1701.379 | 3.764896 | 0.028087 |
| ENSMUSG00000039450 | *Dcxr* | 759.5101 | 4.514582 | 0.028131 |
| ENSMUSG00000022863 | *Btg3* | 45.57894 | 3.26378 | 0.028174 |
| ENSMUSG00000092376 | *Gm19203* | 941.4704 | 3.793856 | 0.028213 |
| ENSMUSG00000034109 | *Golim4* | 10543.14 | 2.638694 | 0.028264 |
| ENSMUSG00000040928 | *S100pbp* | 1489.7 | 3.050014 | 0.028277 |
| ENSMUSG00000066357 | *Wdr6* | 2580.131 | 2.992279 | 0.028282 |
| ENSMUSG00000030068 | *Gm20696* | 31.06798 | 3.110413 | 0.028353 |
| ENSMUSG00000018507 | *Trpv2* | 17.13312 | 3.003067 | 0.028415 |
| ENSMUSG00000082693 | *Gm15190* | 5.608829 | 2.708546 | 0.028418 |
| ENSMUSG00000057691 | *Zfp746* | 1058.155 | 3.855386 | 0.028437 |
| ENSMUSG00000040721 | *Zfhx2* | 1632.79 | 2.634872 | 0.028456 |
| ENSMUSG00000038733 | *Wdr26* | 20525.05 | 2.973828 | 0.028462 |
| ENSMUSG00000030956 | *Fam53b* | 1215.174 | 3.602082 | 0.02857 |
| ENSMUSG00000115219 | *Eef1akmt4* | 20.25465 | 2.934502 | 0.028612 |
| ENSMUSG00000038503 | *Mesd* | 2867.594 | 3.420097 | 0.028644 |
| ENSMUSG00000029265 | *Dr1* | 618.0317 | 3.401737 | 0.028666 |
| ENSMUSG00000038495 | *Otud7b* | 5033.178 | 2.854058 | 0.02873 |
| ENSMUSG00000024140 | *Epas1* | 26387.59 | 2.747064 | 0.028734 |
| ENSMUSG00000027800 | *Tm4sf1* | 4.397123 | 2.112797 | 0.028816 |
| ENSMUSG00000055491 | *Pprc1* | 5725.148 | 2.713471 | 0.028883 |
| ENSMUSG00000027132 | *Katnbl1* | 34.57105 | 3.096118 | 0.028893 |
| ENSMUSG00000019817 | *Plagl1* | 7943.511 | 2.939477 | 0.028912 |
| ENSMUSG00000020516 | *Rps6kb1* | 1032.92 | 3.34357 | 0.028935 |
| ENSMUSG00000035356 | *Nfkbiz* | 370.6691 | 3.645351 | 0.028962 |
| ENSMUSG00000030980 | *Knop1* | 4778.086 | 2.990754 | 0.029091 |
| ENSMUSG00000056586 | *Zar1l* | 129.4816 | 3.828411 | 0.029294 |
| ENSMUSG00000045103 | *Dmd* | 332.9988 | 3.244117 | 0.029355 |
| ENSMUSG00000022336 | *Eif3e* | 587.6795 | 3.501974 | 0.029361 |
| ENSMUSG00000061028 | *Clasrp* | 3422.142 | 2.85481 | 0.029413 |
| ENSMUSG00000046962 | *Zbtb21* | 1329.629 | 3.26874 | 0.029469 |
| ENSMUSG00000024528 | *Srfbp1* | 1406.755 | 3.911334 | 0.029513 |
| ENSMUSG00000057894 | *Zfp329* | 713.0202 | 3.767765 | 0.029547 |
| ENSMUSG00000082016 | *Pgam1* | 722.9059 | 3.95087 | 0.029586 |
| ENSMUSG00000024327 | *Slc39a7* | 11839.66 | 2.159699 | 0.02962 |
| ENSMUSG00000028706 | *Nsun4* | 200.9491 | 3.784613 | 0.029635 |
| ENSMUSG00000003402 | *Prkcsh* | 17707.08 | 2.566166 | 0.029784 |
| ENSMUSG00000024074 | *Crim1* | 29784.52 | 2.196944 | 0.029787 |
| ENSMUSG00000004885 | *Crabp2* | 2498.761 | 3.729822 | 0.029813 |
| ENSMUSG00000031302 | *Nlgn3* | 18.39682 | 2.791469 | 0.029827 |
| ENSMUSG00000038954 | *Supt3* | 2932.537 | 3.493131 | 0.02986 |
| ENSMUSG00000048626 | *Klf17* | 70.6825 | 3.306712 | 0.029879 |
| ENSMUSG00000109022 | *Olfr1432* | 326.051 | 3.207211 | 0.029894 |
| ENSMUSG00000006732 | *Mettl1* | 1484.401 | 4.127533 | 0.029952 |
| ENSMUSG00000041313 | *Slc7a1* | 4510.98 | 2.89701 | 0.029955 |
| ENSMUSG00000018974 | *Sart3* | 6014.398 | 3.009178 | 0.029966 |
| ENSMUSG00000020205 | *Phlda1* | 275.2534 | 3.606782 | 0.029996 |
| ENSMUSG00000059291 | *Rpl11* | 6770.568 | 3.049025 | 0.030103 |
| ENSMUSG00000018569 | *Cldn7* | 10.30439 | 2.860685 | 0.030145 |
| ENSMUSG00000021250 | *Fos* | 2485.709 | 3.678623 | 0.03015 |
| ENSMUSG00000079553 | *Kifc1* | 3230.916 | 3.782932 | 0.030203 |
| ENSMUSG00000022201 | *Zfr* | 15116.96 | 2.707823 | 0.030229 |
| ENSMUSG00000024941 | *Scyl1* | 3189.043 | 3.29366 | 0.030365 |
| ENSMUSG00000033960 | *Jcad* | 2039.229 | 3.636812 | 0.030404 |
| ENSMUSG00000058056 | *Palld* | 771.2099 | 3.652906 | 0.030419 |
| ENSMUSG00000066838 | *Zfp772* | 14.06647 | 2.735495 | 0.030463 |
| ENSMUSG00000034612 | *Chst11* | 23057.3 | 2.398174 | 0.030531 |
| ENSMUSG00000042744 | *Hectd4* | 9908.14 | 2.815935 | 0.03055 |
| ENSMUSG00000027411 | *Vps16* | 1169.048 | 3.355745 | 0.030612 |
| ENSMUSG00000026193 | *Fn1* | 21273.13 | 2.220356 | 0.030633 |
| ENSMUSG00000105189 | *Gm7774* | 6.492312 | 2.411597 | 0.030639 |
| ENSMUSG00000026042 | *Col5a2* | 839.8137 | 3.424884 | 0.030651 |
| ENSMUSG00000023055 | *Calcoco1* | 3384.709 | 3.177625 | 0.030658 |
| ENSMUSG00000022702 | *Hira* | 3077.135 | 3.62692 | 0.030677 |
| ENSMUSG00000081642 | *Gm13532* | 677.564 | 3.95807 | 0.030714 |
| ENSMUSG00000020495 | *Smg8* | 585.9867 | 3.522632 | 0.030734 |
| ENSMUSG00000061288 | *Taok3* | 822.972 | 3.626869 | 0.030861 |
| ENSMUSG00000046179 | *E2f8* | 414.4161 | 3.49763 | 0.030872 |
| ENSMUSG00000042492 | *Tbc1d10b* | 9092.682 | 2.980254 | 0.030987 |
| ENSMUSG00000031093 | *Dock11* | 27.06411 | 2.959757 | 0.031023 |
| ENSMUSG00000032126 | *Hmbs* | 889.9119 | 4.200751 | 0.031078 |
| ENSMUSG00000040118 | *Cacna2d1* | 2429.767 | 3.49828 | 0.031201 |
| ENSMUSG00000004100 | *Ppan* | 6316.482 | 2.452492 | 0.03122 |
| ENSMUSG00000071073 | *Lrrc73* | 5.764743 | 2.249205 | 0.031236 |
| ENSMUSG00000040565 | *Btaf1* | 647.8863 | 2.858617 | 0.031248 |
| ENSMUSG00000023039 | *Krt7* | 210.8267 | 3.379588 | 0.031332 |
| ENSMUSG00000028693 | *Nasp* | 1151.04 | 3.50276 | 0.031361 |
| ENSMUSG00000032078 | *Zpr1* | 989.0715 | 3.011678 | 0.03144 |
| ENSMUSG00000024472 | *Dcp2* | 651.6565 | 3.119032 | 0.03144 |
| ENSMUSG00000031901 | *Dus2* | 101.5091 | 3.34855 | 0.031496 |
| ENSMUSG00000055216 | *9430025C20Rik* | 8.776431 | 2.861413 | 0.03151 |
| ENSMUSG00000114999 | *AC166491* | 5.230255 | 2.302965 | 0.031532 |
| ENSMUSG00000050822 | *Slc29a4* | 8.183239 | 2.405546 | 0.031633 |
| ENSMUSG00000001525 | *Tubb5* | 37965.05 | 1.614073 | 0.031671 |
| ENSMUSG00000063605 | *Ccdc102a* | 564.588 | 4.338463 | 0.031672 |
| ENSMUSG00000015869 | *Prpsap1* | 3269.688 | 3.155319 | 0.031688 |
| ENSMUSG00000022676 | *Snai2* | 352.8494 | 4.059041 | 0.031732 |
| ENSMUSG00000093674 | *Rpl41* | 37468.12 | 2.019784 | 0.031793 |
| ENSMUSG00000069565 | *Dazap1* | 3108.652 | 3.329112 | 0.03181 |
| ENSMUSG00000055681 | *Cope* | 3191.149 | 3.026685 | 0.03184 |
| ENSMUSG00000039697 | *Ncoa7* | 3694.231 | 3.420822 | 0.031864 |
| ENSMUSG00000022971 | *Ifnar2* | 766.7687 | 3.772523 | 0.031951 |
| ENSMUSG00000059669 | *Taf1b* | 55.33748 | 3.293367 | 0.031963 |
| ENSMUSG00000056476 | *Med12l* | 39.50694 | 3.069412 | 0.031971 |
| ENSMUSG00000038646 | *Fam103a1* | 84.11927 | 3.852506 | 0.031977 |
| ENSMUSG00000034663 | *Bmp2k* | 2816.645 | 3.061331 | 0.032019 |
| ENSMUSG00000078652 | *Psme3* | 5605.75 | 2.247218 | 0.032029 |
| ENSMUSG00000031197 | *Vbp1* | 8.120793 | 2.274595 | 0.032098 |
| ENSMUSG00000040950 | *Mgl2* | 7.677718 | 2.504881 | 0.032215 |
| ENSMUSG00000061665 | *Cd2ap* | 729.9994 | 2.863694 | 0.032313 |
| ENSMUSG00000026238 | *Ptma* | 202951.4 | 2.363138 | 0.032447 |
| ENSMUSG00000004864 | *Mapk13* | 193.704 | 3.689995 | 0.032505 |
| ENSMUSG00000039286 | *Fndc3b* | 29596.99 | 2.374329 | 0.032603 |
| ENSMUSG00000021477 | *Ctsl* | 112760.7 | 2.111834 | 0.032775 |
| ENSMUSG00000040728 | *Esrp1* | 27.35561 | 2.919922 | 0.032801 |
| ENSMUSG00000030727 | *Rabep2* | 1412.355 | 3.878472 | 0.032841 |
| ENSMUSG00000002658 | *Gtf2f1* | 4553.46 | 3.126595 | 0.032871 |
| ENSMUSG00000020015 | *Cdk17* | 931.8374 | 3.866635 | 0.032875 |
| ENSMUSG00000026637 | *Traf5* | 556.0828 | 3.768869 | 0.032889 |
| ENSMUSG00000038059 | *Smim3* | 393.1047 | 3.4 | 0.032926 |
| ENSMUSG00000038608 | *Dock10* | 66.99355 | 3.337518 | 0.032932 |
| ENSMUSG00000021957 | *Tkt* | 9248.957 | 2.486402 | 0.032993 |
| ENSMUSG00000024232 | *Bambi* | 14.15546 | 2.669289 | 0.033007 |
| ENSMUSG00000069267 | *Hist1h3b* | 1909.315 | 3.57255 | 0.03318 |
| ENSMUSG00000022554 | *Hgh1* | 89.02941 | 3.295409 | 0.033223 |
| ENSMUSG00000023723 | *Mrps23* | 3248.253 | 3.350667 | 0.03327 |
| ENSMUSG00000051669 | *AU021092* | 372.8944 | 3.726667 | 0.033326 |
| ENSMUSG00000030400 | *Ercc2* | 1915.604 | 3.452603 | 0.033351 |
| ENSMUSG00000032515 | *Csrnp1* | 159.978 | 3.575888 | 0.033367 |
| ENSMUSG00000023932 | *Cdc5l* | 354.0248 | 3.339918 | 0.033544 |
| ENSMUSG00000033763 | *Mtss1l* | 3450.307 | 3.578593 | 0.033581 |
| ENSMUSG00000079227 | *Ccr5* | 9559.185 | 2.96713 | 0.033633 |
| ENSMUSG00000022538 | *Lsg1* | 1440.503 | 3.809482 | 0.033698 |
| ENSMUSG00000038175 | *Mylip* | 623.0762 | 3.999882 | 0.033704 |
| ENSMUSG00000026788 | *Zbtb43* | 664.2768 | 3.751273 | 0.033748 |
| ENSMUSG00000029669 | *Tspan12* | 176.4157 | 3.734296 | 0.033752 |
| ENSMUSG00000060152 | *Pop5* | 246.0252 | 2.818968 | 0.033764 |
| ENSMUSG00000018574 | *Acadvl* | 1994.789 | 2.927912 | 0.033788 |
| ENSMUSG00000058542 | *Gm15590* | 20.56291 | 2.523315 | 0.033807 |
| ENSMUSG00000026942 | *Traf2* | 741.1971 | 3.166764 | 0.033844 |
| ENSMUSG00000031994 | *Adamts8* | 6.386256 | 2.741418 | 0.033878 |
| ENSMUSG00000036054 | *Sugp2* | 1610.888 | 3.132172 | 0.033957 |
| ENSMUSG00000030536 | *Iqgap1* | 12347.94 | 2.179854 | 0.0341 |
| ENSMUSG00000027894 | *Slc6a17* | 2390.059 | 3.688298 | 0.034171 |
| ENSMUSG00000005583 | *Mef2c* | 1089.402 | 3.087729 | 0.034185 |
| ENSMUSG00000060373 | *Hnrnpc* | 4317.65 | 2.601615 | 0.034352 |
| ENSMUSG00000054083 | *Capn12* | 7.565073 | 2.503308 | 0.034353 |
| ENSMUSG00000026223 | *Itm2c* | 6099.155 | 2.547256 | 0.034422 |
| ENSMUSG00000090946 | *Ccdc71l* | 48.18768 | 3.296473 | 0.034586 |
| ENSMUSG00000034413 | *Neurl1b* | 686.2629 | 3.127029 | 0.034613 |
| ENSMUSG00000024780 | *Cdc37l1* | 664.6373 | 2.871565 | 0.034695 |
| ENSMUSG00000078517 | *Emc1* | 10813.22 | 2.75243 | 0.034739 |
| ENSMUSG00000053560 | *Ier2* | 732.5001 | 3.526335 | 0.034749 |
| ENSMUSG00000005204 | *Senp3* | 2106.285 | 2.927827 | 0.034832 |
| ENSMUSG00000032818 | *Loxhd1* | 28.89043 | 2.99956 | 0.034854 |
| ENSMUSG00000003437 | *Paf1* | 6141.781 | 3.196528 | 0.034876 |
| ENSMUSG00000024480 | *Ap3s1* | 55.66766 | 3.157224 | 0.034907 |
| ENSMUSG00000057596 | *Trim30d* | 4.085131 | 1.987034 | 0.034954 |
| ENSMUSG00000047793 | *Sned1* | 2605.661 | 2.412051 | 0.034989 |
| ENSMUSG00000037958 | *Nsrp1* | 1597.976 | 3.19345 | 0.035033 |
| ENSMUSG00000045128 | *Rpl18a* | 20261.1 | 2.261057 | 0.035076 |
| ENSMUSG00000025858 | *Get4* | 2206.609 | 3.855292 | 0.035284 |
| ENSMUSG00000042312 | *S100a13* | 59.84166 | 3.046161 | 0.03529 |
| ENSMUSG00000022822 | *Abcc5* | 2708.189 | 3.023745 | 0.035325 |
| ENSMUSG00000040855 | *Reps2* | 17.42544 | 2.833951 | 0.035366 |
| ENSMUSG00000030259 | *Rassf8* | 11592.08 | 2.995381 | 0.035387 |
| ENSMUSG00000021690 | *Jmy* | 5196.868 | 2.588846 | 0.035479 |
| ENSMUSG00000049148 | *Plcxd3* | 25.37781 | 3.27345 | 0.035487 |
| ENSMUSG00000032308 | *Ulk3* | 1736.53 | 3.184516 | 0.035533 |
| ENSMUSG00000007833 | *Aldh16a1* | 1254.451 | 3.788019 | 0.03554 |
| ENSMUSG00000031447 | *Lamp1* | 15458.26 | 2.81299 | 0.035546 |
| ENSMUSG00000046434 | *Hnrnpa1* | 11068.84 | 2.972307 | 0.035655 |
| ENSMUSG00000026753 | *Ppp6c* | 399.3942 | 3.477984 | 0.035666 |
| ENSMUSG00000071036 | *Gm10309* | 5.570237 | 2.241448 | 0.03567 |
| ENSMUSG00000027937 | *Jtb* | 420.2563 | 3.596179 | 0.035719 |
| ENSMUSG00000051998 | *Lax1* | 3.842025 | 2.055747 | 0.035736 |
| ENSMUSG00000052981 | *Ube2ql1* | 117.2674 | 3.716836 | 0.03578 |
| ENSMUSG00000044456 | *Rin3* | 372.354 | 3.689335 | 0.035789 |
| ENSMUSG00000042606 | *Hirip3* | 13049.84 | 3.184422 | 0.035834 |
| ENSMUSG00000060992 | *Copz1* | 889.2125 | 2.955633 | 0.035855 |
| ENSMUSG00000033685 | *Ucp2* | 16129.16 | 2.105064 | 0.035892 |
| ENSMUSG00000106067 | *Gm7902* | 577.1808 | 3.978896 | 0.035979 |
| ENSMUSG00000058360 | *Gm10040* | 606.3685 | 3.675122 | 0.035993 |
| ENSMUSG00000045731 | *Pnoc* | 464.2013 | 3.621672 | 0.035995 |
| ENSMUSG00000045576 | *St7l* | 1305.15 | 3.401174 | 0.036057 |
| ENSMUSG00000025586 | *Cpeb1* | 78.84923 | 3.604413 | 0.036174 |
| ENSMUSG00000074997 | *Pin1rt1* | 20.34439 | 2.964034 | 0.036229 |
| ENSMUSG00000061613 | *U2af1* | 1767.331 | 3.070336 | 0.03626 |
| ENSMUSG00000059495 | *Arhgef12* | 9137.066 | 2.024532 | 0.0363 |
| ENSMUSG00000002963 | *Pnkp* | 1773.867 | 3.1118 | 0.036336 |
| ENSMUSG00000019471 | *Cdc37* | 9833.977 | 2.784216 | 0.036423 |
| ENSMUSG00000016356 | *Col20a1* | 744.499 | 3.252299 | 0.036455 |
| ENSMUSG00000076431 | *Sox4* | 7174.399 | 2.473547 | 0.036518 |
| ENSMUSG00000021156 | *Zmynd11* | 4541.223 | 2.881229 | 0.03659 |
| ENSMUSG00000029406 | *Pitpnm2* | 1723.092 | 3.117027 | 0.036667 |
| ENSMUSG00000026234 | *Ncl* | 103888.7 | 1.986509 | 0.036766 |
| ENSMUSG00000020021 | *Fgd6* | 30.9272 | 2.539602 | 0.036812 |
| ENSMUSG00000020802 | *Ube2o* | 5703.693 | 2.797713 | 0.036837 |
| ENSMUSG00000089762 | *Ier5l* | 72.60255 | 3.252391 | 0.036892 |
| ENSMUSG00000032860 | *P2ry2* | 24.01856 | 3.012337 | 0.036985 |
| ENSMUSG00000024914 | *Drap1* | 1539.442 | 2.729806 | 0.037008 |
| ENSMUSG00000015961 | *Adss* | 1238.335 | 3.043957 | 0.037019 |
| ENSMUSG00000026643 | *Nmt2* | 998.3197 | 2.490152 | 0.037058 |
| ENSMUSG00000016831 | *Tox4* | 903.1744 | 3.615621 | 0.037121 |
| ENSMUSG00000039997 | *Ifi203* | 265.3293 | 3.74465 | 0.037147 |
| ENSMUSG00000054766 | *Set* | 72716.03 | 3.107818 | 0.037153 |
| ENSMUSG00000029730 | *Mcm7* | 3281.067 | 3.088566 | 0.037278 |
| ENSMUSG00000032375 | *Aph1b* | 56.29462 | 3.15551 | 0.037303 |
| ENSMUSG00000022112 | *Gpc5* | 4.32296 | 2.012535 | 0.037434 |
| ENSMUSG00000028603 | *Scp2* | 1933.08 | 3.178082 | 0.037434 |
| ENSMUSG00000020265 | *Sumo3* | 3100.079 | 3.022153 | 0.037466 |
| ENSMUSG00000036636 | *Clcn7* | 6299.729 | 3.028838 | 0.037652 |
| ENSMUSG00000004936 | *Map2k1* | 1313.925 | 3.062623 | 0.037653 |
| ENSMUSG00000001440 | *Kpnb1* | 5953.486 | 1.793599 | 0.03767 |
| ENSMUSG00000031605 | *Klhl2* | 874.9669 | 2.397202 | 0.037679 |
| ENSMUSG00000042548 | *Asxl1* | 3759.875 | 2.567579 | 0.03774 |
| ENSMUSG00000007030 | *Vwa7* | 17.91817 | 2.966571 | 0.037757 |
| ENSMUSG00000019818 | *Cd164* | 3465.019 | 2.895376 | 0.037815 |
| ENSMUSG00000007041 | *Clic1* | 1225.448 | 3.240001 | 0.037823 |
| ENSMUSG00000051627 | *Hist1h1e* | 611.7506 | 2.897819 | 0.037877 |
| ENSMUSG00000020386 | *Sar1b* | 125.9843 | 3.230453 | 0.037943 |
| ENSMUSG00000004110 | *Cacna1e* | 166.0299 | 2.988715 | 0.037958 |
| ENSMUSG00000022636 | *Alcam* | 1481.317 | 3.516703 | 0.037996 |
| ENSMUSG00000089837 | *Npcd* | 58.27153 | 3.042891 | 0.038041 |
| ENSMUSG00000022018 | *Rgcc* | 721.766 | 3.079548 | 0.038112 |
| ENSMUSG00000060126 | *Tpt1* | 19361.11 | 2.415482 | 0.038154 |
| ENSMUSG00000048379 | *Socs4* | 535.2413 | 3.125238 | 0.03817 |
| ENSMUSG00000049551 | *Fzd9* | 5.148769 | 2.242999 | 0.038178 |
| ENSMUSG00000020723 | *Cacng4* | 3625.63 | 4.308237 | 0.038248 |
| ENSMUSG00000085481 | *Gm16304* | 3.839336 | 1.974315 | 0.038249 |
| ENSMUSG00000035722 | *Abca7* | 3406.57 | 3.329251 | 0.038269 |
| ENSMUSG00000038188 | *Scarf1* | 14.42313 | 3.155975 | 0.038298 |
| ENSMUSG00000075146 | *Olfr1154* | 5.071273 | 2.153693 | 0.038328 |
| ENSMUSG00000006262 | *Mob1b* | 5189.782 | 2.966915 | 0.038368 |
| ENSMUSG00000056692 | *D17Wsu92e* | 3095.511 | 2.604704 | 0.038447 |
| ENSMUSG00000041245 | *Wnk3* | 168.5719 | 3.525464 | 0.03845 |
| ENSMUSG00000041836 | *Ptpre* | 1738.261 | 3.204578 | 0.03848 |
| ENSMUSG00000028339 | *Col15a1* | 679.3867 | 3.232543 | 0.038485 |
| ENSMUSG00000031485 | *Plpbp* | 327.3079 | 2.949738 | 0.038562 |
| ENSMUSG00000035696 | *Rnf38* | 12447.9 | 3.005889 | 0.038614 |
| ENSMUSG00000063316 | *Rpl27* | 376.5772 | 3.63534 | 0.038661 |
| ENSMUSG00000024576 | *Csnk1a1* | 2627.298 | 2.321595 | 0.038785 |
| ENSMUSG00000049556 | *Lingo1* | 36.53442 | 3.144759 | 0.038798 |
| ENSMUSG00000071014 | *Ndufb6* | 60.98693 | 3.118655 | 0.038807 |
| ENSMUSG00000046818 | *Ddit4l* | 1065.647 | 2.944256 | 0.038819 |
| ENSMUSG00000032193 | *Ldlr* | 9725.91 | 2.409622 | 0.038835 |
| ENSMUSG00000064264 | *Zfp428* | 346.6878 | 3.594634 | 0.038887 |
| ENSMUSG00000046186 | *Cd109* | 55.32514 | 2.977212 | 0.038918 |
| ENSMUSG00000021144 | *Mta1* | 3964.309 | 2.387227 | 0.039004 |
| ENSMUSG00000055128 | *Cgrrf1* | 51.37219 | 2.905448 | 0.039041 |
| ENSMUSG00000042515 | *Mum1l1* | 58.52767 | 3.203887 | 0.039117 |
| ENSMUSG00000028358 | *Zfp618* | 2984.617 | 3.18787 | 0.039132 |
| ENSMUSG00000038482 | *Tfdp1* | 581.1352 | 3.379209 | 0.039152 |
| ENSMUSG00000029674 | *Limk1* | 914.2889 | 3.557845 | 0.039173 |
| ENSMUSG00000015087 | *Rabl6* | 4455.605 | 2.742252 | 0.039187 |
| ENSMUSG00000028266 | *Lmo4* | 1053.539 | 3.028952 | 0.039191 |
| ENSMUSG00000017478 | *Zc3h18* | 9692.512 | 2.132549 | 0.03923 |
| ENSMUSG00000022956 | *Atp5o* | 4057.902 | 3.174893 | 0.039264 |
| ENSMUSG00000010461 | *Eya4* | 1478.333 | 3.565506 | 0.039297 |
| ENSMUSG00000078810 | *Gp6* | 4.42834 | 1.998286 | 0.039484 |
| ENSMUSG00000061104 | *Sap18b* | 80.41538 | 3.372329 | 0.03952 |
| ENSMUSG00000024590 | *Lmnb1* | 16374.44 | 2.575586 | 0.039579 |
| ENSMUSG00000039929 | *Urb1* | 1086.041 | 3.109567 | 0.039588 |
| ENSMUSG00000033502 | *Cdc14a* | 940.0981 | 3.284258 | 0.03984 |
| ENSMUSG00000040297 | *Suco* | 3359.296 | 2.588112 | 0.039961 |
| ENSMUSG00000044496 | *2510039O18Rik* | 28105.43 | 2.783335 | 0.040069 |
| ENSMUSG00000040612 | *Ildr2* | 327.5799 | 3.324194 | 0.040122 |
| ENSMUSG00000074102 | *Rbm15b* | 1877.27 | 2.735804 | 0.040153 |
| ENSMUSG00000025809 | *Itgb1* | 9065.189 | 2.052603 | 0.040187 |
| ENSMUSG00000038402 | *Foxf2* | 593.0132 | 3.937126 | 0.040221 |
| ENSMUSG00000020532 | *Acaca* | 3994.386 | 2.429891 | 0.040234 |
| ENSMUSG00000021102 | *Glrx5* | 396.4222 | 3.491676 | 0.040249 |
| ENSMUSG00000039714 | *Cplx3* | 8.496726 | 2.706606 | 0.040395 |
| ENSMUSG00000081094 | *Rpl19* | 2531.375 | 5.327898 | 0.04042 |
| ENSMUSG00000098078 | *Gm26992* | 7423.493 | 2.917209 | 0.040427 |
| ENSMUSG00000033819 | *Ppp1r16a* | 2597.076 | 3.772028 | 0.040456 |
| ENSMUSG00000028635 | *Edn2* | 3.565382 | 2.04959 | 0.040517 |
| ENSMUSG00000029518 | *Rab35* | 829.8539 | 2.828817 | 0.040556 |
| ENSMUSG00000026923 | *Notch1* | 3374.72 | 4.035177 | 0.040615 |
| ENSMUSG00000031778 | *Cx3cl1* | 1548.008 | 3.784863 | 0.04064 |
| ENSMUSG00000068252 | *Apol7b* | 115.129 | 3.066929 | 0.040687 |
| ENSMUSG00000021782 | *Dlg5* | 7539.787 | 2.426219 | 0.040746 |
| ENSMUSG00000034120 | *Srsf2* | 4540.725 | 2.298014 | 0.040831 |
| ENSMUSG00000032727 | *Mier3* | 452.7826 | 3.053544 | 0.040883 |
| ENSMUSG00000070856 | *Olfr1115* | 7.060919 | 2.415681 | 0.040886 |
| ENSMUSG00000000751 | *Rpa1* | 2912.61 | 3.294142 | 0.040888 |
| ENSMUSG00000031278 | *Acsl4* | 1655.139 | 3.043213 | 0.040948 |
| ENSMUSG00000040667 | *Nup88* | 1301.86 | 2.739943 | 0.041025 |
| ENSMUSG00000020571 | *Pdia6* | 7485.407 | 2.737129 | 0.041031 |
| ENSMUSG00000115792 | *Vmn1r16* | 12.33932 | 2.660641 | 0.041066 |
| ENSMUSG00000073982 | *Rhog* | 1120.911 | 3.20261 | 0.041085 |
| ENSMUSG00000024188 | *Luc7l* | 2175.597 | 2.782972 | 0.041138 |
| ENSMUSG00000034300 | *Fam53c* | 2170.725 | 2.612507 | 0.04123 |
| ENSMUSG00000106248 | *Gm9728* | 4.826699 | 2.147488 | 0.041286 |
| ENSMUSG00000034903 | *Cobll1* | 7721.9 | 2.969443 | 0.041315 |
| ENSMUSG00000038250 | *Usp38* | 493.0197 | 3.499718 | 0.041362 |
| ENSMUSG00000031785 | *Adgrg1* | 34427.68 | 2.660175 | 0.041409 |
| ENSMUSG00000070645 | *Ren1* | 315.0585 | 3.001368 | 0.041502 |
| ENSMUSG00000028552 | *Eps15* | 598.2192 | 3.409166 | 0.041656 |
| ENSMUSG00000036678 | *Aaas* | 360.4382 | 2.690985 | 0.041668 |
| ENSMUSG00000031729 | *Ist1* | 1664.666 | 2.763285 | 0.041669 |
| ENSMUSG00000031534 | *Smim19* | 267.2062 | 3.437757 | 0.041777 |
| ENSMUSG00000045225 | *Olfr1152* | 101.6112 | 3.481814 | 0.041868 |
| ENSMUSG00000051329 | *Nup160* | 301.4799 | 3.021607 | 0.041902 |
| ENSMUSG00000033014 | *Trim33* | 586.2061 | 2.640746 | 0.041905 |
| ENSMUSG00000040850 | *Psme4* | 1535.501 | 2.494873 | 0.041999 |
| ENSMUSG00000043467 | *Zbtb37* | 1305.503 | 3.796635 | 0.042005 |
| ENSMUSG00000054717 | *Hmgb2* | 28783.51 | 3.64276 | 0.042047 |
| ENSMUSG00000021413 | *Prpf4b* | 3815.989 | 2.764609 | 0.042052 |
| ENSMUSG00000068923 | *Syt11* | 648.6472 | 3.149931 | 0.042143 |
| ENSMUSG00000059323 | *Tonsl* | 879.4464 | 3.020551 | 0.042186 |
| ENSMUSG00000045302 | *Preb* | 5784.268 | 2.578697 | 0.042208 |
| ENSMUSG00000032511 | *Scn5a* | 897.7254 | 3.942457 | 0.042322 |
| ENSMUSG00000001472 | *Tcf25* | 9109.856 | 2.004185 | 0.042335 |
| ENSMUSG00000026810 | *Dpm2* | 155.4614 | 2.885905 | 0.042364 |
| ENSMUSG00000028649 | *Macf1* | 16240.19 | 2.142576 | 0.042378 |
| ENSMUSG00000025737 | *Wdr24* | 3457.666 | 3.576336 | 0.042435 |
| ENSMUSG00000020477 | *Mrps24* | 28.4504 | 2.474797 | 0.042481 |
| ENSMUSG00000047187 | *Rab2a* | 3117.894 | 2.441088 | 0.042487 |
| ENSMUSG00000041057 | *Wdr43* | 2670.958 | 2.817406 | 0.042508 |
| ENSMUSG00000019428 | *Fkbp8* | 4079.235 | 2.734311 | 0.042544 |
| ENSMUSG00000040123 | *Zmym5* | 826.4569 | 3.450471 | 0.042595 |
| ENSMUSG00000068267 | *Cenpb* | 49936.34 | 3.350065 | 0.042642 |
| ENSMUSG00000019863 | *Qrsl1* | 23.26294 | 2.947316 | 0.042649 |
| ENSMUSG00000024831 | *Ighmbp2* | 292.4091 | 3.462838 | 0.042668 |
| ENSMUSG00000014355 | *Anapc1* | 4529.984 | 2.13247 | 0.042715 |
| ENSMUSG00000045349 | *Sh2d5* | 132.1168 | 3.22751 | 0.042942 |
| ENSMUSG00000021509 | *Slc25a48* | 81.38206 | 3.095752 | 0.042943 |
| ENSMUSG00000029822 | *Osbpl3* | 4198.748 | 3.105836 | 0.042963 |
| ENSMUSG00000018761 | *Mpdu1* | 370.3979 | 2.421261 | 0.043038 |
| ENSMUSG00000014551 | *Mrps25* | 513.7172 | 3.285719 | 0.043069 |
| ENSMUSG00000022817 | *Itgb5* | 4912.019 | 3.093154 | 0.04308 |
| ENSMUSG00000050640 | *Tmem150c* | 15.207 | 2.798645 | 0.043106 |
| ENSMUSG00000037315 | *Jade3* | 46.55333 | 2.591334 | 0.043254 |
| ENSMUSG00000051978 | *Erich1* | 529.529 | 3.915129 | 0.043318 |
| ENSMUSG00000037089 | *Slc35b2* | 2673.574 | 3.177677 | 0.043334 |
| ENSMUSG00000037370 | *Enpp1* | 657.8841 | 3.241738 | 0.04334 |
| ENSMUSG00000048636 | *A730049H05Rik* | 705.8556 | 3.451089 | 0.043453 |
| ENSMUSG00000038418 | *Egr1* | 7763.486 | 3.023827 | 0.043469 |
| ENSMUSG00000047649 | *Cd3eap* | 59.18543 | 2.902978 | 0.043481 |
| ENSMUSG00000094248 | *Hist1h2ao* | 2089.467 | 3.246655 | 0.043502 |
| ENSMUSG00000057322 | *Rpl38* | 713.1398 | 3.109753 | 0.043569 |
| ENSMUSG00000006344 | *Ggt5* | 38.94553 | 2.973872 | 0.043575 |
| ENSMUSG00000021962 | *Dcp1a* | 1671.173 | 3.015158 | 0.043633 |
| ENSMUSG00000053113 | *Socs3* | 298.0247 | 3.12613 | 0.043644 |
| ENSMUSG00000047907 | *Tshz2* | 9735.382 | 3.587442 | 0.043748 |
| ENSMUSG00000030103 | *Bhlhe40* | 1789.952 | 2.950045 | 0.043771 |
| ENSMUSG00000046562 | *Unc119b* | 1689.469 | 2.899795 | 0.043881 |
| ENSMUSG00000114603 | *AC187103* | 4.571307 | 2.098993 | 0.044138 |
| ENSMUSG00000043881 | *Kbtbd7* | 153.5229 | 3.343726 | 0.044142 |
| ENSMUSG00000031924 | *Cyb5b* | 3505.464 | 2.700956 | 0.044248 |
| ENSMUSG00000070378 | *Olfr403* | 13.05889 | 2.795847 | 0.044282 |
| ENSMUSG00000004562 | *Arhgef40* | 1912.053 | 2.745335 | 0.044296 |
| ENSMUSG00000000959 | *Oxa1l* | 2448.063 | 3.050256 | 0.044419 |
| ENSMUSG00000058835 | *Abi1* | 2833.238 | 3.423045 | 0.044442 |
| ENSMUSG00000046668 | *Cxxc5* | 1323.324 | 3.600965 | 0.044553 |
| ENSMUSG00000023048 | *Prr13* | 1839.346 | 2.987421 | 0.044668 |
| ENSMUSG00000028868 | *Wasf2* | 7230.018 | 2.663446 | 0.044704 |
| ENSMUSG00000071054 | *Safb* | 12291.24 | 2.544096 | 0.044824 |
| ENSMUSG00000048546 | *Tob2* | 1521.331 | 3.179565 | 0.044872 |
| ENSMUSG00000004393 | *Ddx56* | 1316.065 | 3.339816 | 0.04488 |
| ENSMUSG00000017667 | *Zfp334* | 5500.671 | 3.768011 | 0.044886 |
| ENSMUSG00000007880 | *Arid1a* | 15199.64 | 1.99622 | 0.0451 |
| ENSMUSG00000024429 | *Gnl1* | 5708.571 | 3.092737 | 0.04523 |
| ENSMUSG00000086688 | *Gm11560* | 3.875941 | 1.852818 | 0.045266 |
| ENSMUSG00000050043 | *Tmx2* | 182.8949 | 2.904341 | 0.045278 |
| ENSMUSG00000001657 | *Hoxc8* | 67.96148 | 3.275569 | 0.045289 |
| ENSMUSG00000020728 | *Cep112* | 814.0271 | 3.919921 | 0.045292 |
| ENSMUSG00000019254 | *Ppp1r12c* | 6210.246 | 2.580615 | 0.045294 |
| ENSMUSG00000083829 | *Gm2199* | 4.315661 | 2.007235 | 0.045348 |
| ENSMUSG00000030357 | *Fkbp4* | 14532.33 | 2.845337 | 0.045357 |
| ENSMUSG00000040356 | *Skiv2l* | 3922.699 | 2.745039 | 0.045475 |
| ENSMUSG00000028173 | *Wls* | 682.162 | 3.264368 | 0.045487 |
| ENSMUSG00000022769 | *Sdf2l1* | 891.9886 | 3.530195 | 0.045594 |
| ENSMUSG00000075318 | *Scn2a* | 293.1931 | 3.725682 | 0.045677 |
| ENSMUSG00000001016 | *Ilf2* | 886.1524 | 3.046409 | 0.045738 |
| ENSMUSG00000030530 | *Furin* | 3784.599 | 2.452581 | 0.045802 |
| ENSMUSG00000040430 | *Pitpnc1* | 886.2287 | 3.090215 | 0.045826 |
| ENSMUSG00000021820 | *Camk2g* | 2230.977 | 2.184213 | 0.045833 |
| ENSMUSG00000037161 | *Mgarp* | 4728.052 | 2.48914 | 0.045976 |
| ENSMUSG00000004098 | *Col5a3* | 4.096306 | 2.100496 | 0.046076 |
| ENSMUSG00000038893 | *Fam117a* | 157.033 | 3.301291 | 0.046094 |
| ENSMUSG00000012296 | *Tjap1* | 2084.733 | 3.118694 | 0.046121 |
| ENSMUSG00000025876 | *Unc5a* | 7.401904 | 2.449947 | 0.046159 |
| ENSMUSG00000032580 | *Rbm5* | 4132.79 | 2.874936 | 0.046221 |
| ENSMUSG00000020101 | *Vsir* | 154.8277 | 3.181691 | 0.046263 |
| ENSMUSG00000041570 | *Camsap2* | 1839.207 | 2.986279 | 0.046279 |
| ENSMUSG00000060475 | *Wtap* | 399.2745 | 3.078826 | 0.046401 |
| ENSMUSG00000003234 | *Abcf3* | 2377.95 | 2.891397 | 0.046437 |
| ENSMUSG00000023032 | *Slc4a8* | 2340.547 | 3.226318 | 0.046458 |
| ENSMUSG00000015092 | *Edf1* | 4955.223 | 2.668293 | 0.04672 |
| ENSMUSG00000022421 | *Nptxr* | 182.7158 | 3.281754 | 0.046733 |
| ENSMUSG00000057446 | *Cts8* | 7.40568 | 2.360814 | 0.046739 |
| ENSMUSG00000022665 | *Ccdc80* | 499.1956 | 2.984437 | 0.046829 |
| ENSMUSG00000007908 | *Hmgcll1* | 6.897298 | 1.954336 | 0.04683 |
| ENSMUSG00000029430 | *Ran* | 3077.766 | 2.436372 | 0.046836 |
| ENSMUSG00000028599 | *Tnfrsf1b* | 951.9556 | 3.558442 | 0.046951 |
| ENSMUSG00000014077 | *Chp1* | 2259.948 | 2.801414 | 0.047128 |
| ENSMUSG00000071655 | *Ubxn1* | 6295.223 | 2.26744 | 0.047177 |
| ENSMUSG00000061518 | *Cox5b* | 1035.156 | 3.360753 | 0.047204 |
| ENSMUSG00000002307 | *Daxx* | 4819.463 | 2.63112 | 0.047267 |
| ENSMUSG00000029061 | *Mmp23* | 825.2742 | 3.177106 | 0.047278 |
| ENSMUSG00000071454 | *Dtnb* | 5717.434 | 2.579225 | 0.047356 |
| ENSMUSG00000027080 | *Med19* | 122.4451 | 3.389409 | 0.047358 |
| ENSMUSG00000021794 | *Glud1* | 2289.15 | 3.09943 | 0.04737 |
| ENSMUSG00000036606 | *Plxnb2* | 37078.97 | 2.157329 | 0.047491 |
| ENSMUSG00000031134 | *Rbmx* | 2048.642 | 3.261292 | 0.047514 |
| ENSMUSG00000022174 | *Dad1* | 737.0614 | 2.804872 | 0.047593 |
| ENSMUSG00000012396 | *Nanog* | 10.67374 | 2.388302 | 0.047633 |
| ENSMUSG00000026566 | *Mpzl1* | 5435.614 | 3.199012 | 0.04771 |
| ENSMUSG00000112023 | *Lilr4b* | 4.282861 | 1.891193 | 0.04774 |
| ENSMUSG00000075334 | *Rprm* | 17.91474 | 2.61469 | 0.047796 |
| ENSMUSG00000083623 | *Gm7224* | 9.163896 | 2.856698 | 0.047816 |
| ENSMUSG00000018293 | *Pfn1* | 6412.469 | 2.219244 | 0.047839 |
| ENSMUSG00000006423 | *C330007P06Rik* | 3612.468 | 2.434751 | 0.047894 |
| ENSMUSG00000074283 | *Zfp109* | 6.794914 | 2.053609 | 0.047957 |
| ENSMUSG00000040435 | *Ppp1r15a* | 5144.921 | 2.713845 | 0.04798 |
| ENSMUSG00000038304 | *Cd160* | 5.05983 | 1.867885 | 0.048053 |
| ENSMUSG00000068290 | *Ddrgk1* | 2854.488 | 3.569957 | 0.048084 |
| ENSMUSG00000023892 | *Zfp51* | 11.13109 | 2.440289 | 0.048095 |
| ENSMUSG00000027238 | *Frmd5* | 17090.16 | 2.049046 | 0.048258 |
| ENSMUSG00000035329 | *Fbxo33* | 172.6484 | 3.009445 | 0.048363 |
| ENSMUSG00000022808 | *Snx4* | 1103.113 | 3.017946 | 0.048394 |
| ENSMUSG00000026181 | *Ppm1f* | 782.8207 | 3.357728 | 0.048398 |
| ENSMUSG00000034994 | *Eef2* | 136057.2 | 1.91962 | 0.048456 |
| ENSMUSG00000036718 | *Micall2* | 47.65195 | 3.195953 | 0.048463 |
| ENSMUSG00000024976 | *Shoc2* | 204.8536 | 3.020746 | 0.048545 |
| ENSMUSG00000017221 | *Psmd3* | 7001.627 | 2.068929 | 0.048571 |
| ENSMUSG00000098180 | *Gm5430* | 6.382107 | 2.191888 | 0.048668 |
| ENSMUSG00000043668 | *Tox3* | 3.968968 | 1.892635 | 0.048672 |
| ENSMUSG00000054737 | *Zfp182* | 22.09155 | 2.821642 | 0.048848 |
| ENSMUSG00000022400 | *Rbx1* | 1093.414 | 3.375336 | 0.049001 |
| ENSMUSG00000035585 | *Tsen34* | 1358.169 | 3.572908 | 0.049088 |
| ENSMUSG00000006215 | *Zbtb17* | 3648.244 | 2.914159 | 0.049106 |
| ENSMUSG00000027401 | *Tgm3* | 9.250194 | 2.454454 | 0.049111 |
| ENSMUSG00000058587 | *Tmod3* | 1186.33 | 2.796348 | 0.049189 |
| ENSMUSG00000055762 | *Eef1d* | 5835.407 | 2.257174 | 0.049271 |
| ENSMUSG00000042185 | *Nfrkb* | 14071.89 | 3.346296 | 0.049304 |
| ENSMUSG00000060467 | *Gm10080* | 4.933571 | 1.907876 | 0.049309 |
| ENSMUSG00000001270 | *Ckb* | 3574.633 | 3.87737 | 0.049397 |
| ENSMUSG00000061589 | *Dot1l* | 7600.818 | 2.090695 | 0.049402 |
| ENSMUSG00000041359 | *Tcl1* | 761.5472 | 3.612596 | 0.049506 |
| ENSMUSG00000020781 | *Tsen54* | 152.5441 | 3.012096 | 0.049534 |
| ENSMUSG00000029580 | *Actb* | 137125.7 | 1.765408 | 0.049635 |
| ENSMUSG00000026917 | *Wdr5* | 745.9891 | 2.811526 | 0.049659 |
| ENSMUSG00000044367 | *Slc16a13* | 294.7317 | 3.22004 | 0.049662 |
| ENSMUSG00000002897 | *Il17ra* | 3197.572 | 2.482293 | 0.049719 |
| ENSMUSG00000034832 | *Tet3* | 8208.824 | 1.679088 | 0.049877 |
| ENSMUSG00000029714 | *Gigyf1* | 7269.952 | 2.330226 | 0.049884 |
| ENSMUSG00000053436 | *Mapk14* | 2310.752 | 3.354725 | 0.049894 |
